# Climate Change and Photochemical Ozone Creation Potential Impact Indicators of Cow Milk: A Comparison of Different Scenarios for a Diet Assessment

**DOI:** 10.3390/ani14121725

**Published:** 2024-06-07

**Authors:** Federico Froldi, Lucrezia Lamastra, Marco Trevisan, Maurizio Moschini

**Affiliations:** 1 Department of Animal Science, Food and Nutrition (DiANA), Università Cattolica del Sacro Cuore, 29122 Piacenza, Italy; federico.froldi@unicatt.it; 2 Department for Sustainable Food Process (DiSTAS), Università Cattolica del Sacro Cuore, 29122 Piacenza, Italy; lucrezia.lamastra@unicatt.it (L.L.); marco.trevisan@unicatt.it (M.T.)

**Keywords:** life cycle assessment, climate change, photochemical ozone creation potential, dairy farms, feeding models

## Abstract

**Simple Summary:**

Milk is one of the most globally produced foods with a growing trend, so its environmental performance needs to be studied. This research assessed the environmental impact of cow milk according to herds’ performance through climate change and photochemical ozone creation potential indicators. The quality of feeds was also addressed. Under our conditions, knowing the nutritional characteristics of diets fed to animals as an alternative to tabulated values reduces the estimated impacts of considered indicators.

**Abstract:**

An estimate of the environmental impact of dairy farms in Northern Italy producing milk for hard cheese (protected designation of origin) has been obtained through a comprehensive life cycle assessment. The estimate focused on climate change (CC) and photochemical ozone creation potential (POCP) indicators, which were evaluated according to the Intergovernmental Panel on Climate Change (IPCC) guidelines and interpreted with the aid of the feeds’ composition evaluated using near-infrared reflectance spectroscopy (Foss NIR-System 5000) as well as with a diet evaluation according to the NRC (National Research Council) or the CNCPS (Cornell Net Carbohydrate and Protein System) nutrient requirement modeling. Herds were classified into high-, mid-, and low-performing based on the daily milk yield per cow. A lower impact on indicators was observed as herd performance increased. The high-performing herds had a lower contribution from enteric fermentation (6.30 × 10^−1^ kgCO_2_-eq), and the more milk that they produced allowed for a differentiation of CC from land use and transformation (2.39 × 10^−1^ kgCO_2_-eq), compared to low-performing herds (3.66 × 10^−1^ kgCO_2_-eq). Compared to the IPCC approach, the CC and POCP indicator estimates were reduced when addressing the feed’s quality, particularly in mid- and high-performing herds. The results could be helpful in the dairy sector as they provide an insight into how diet quality affects the environmental impact of milk.

## 1. Introduction

Milk is one of the world’s most produced and valuable animal products [1], whose production is increasing significantly in developing countries, which is relevant to its environmental impact [2]. The use of natural resources (land, water) by livestock farming can lead to high environmental pressure [3], which is expressed in indicators such as climate change, eutrophication, acidification, and water and soil use [4]. The life cycle assessment (LCA) is recognized as one of the most comprehensive and strategic methodological frameworks to study the environmental impact of a product in terms of the use of resources [5,6,7]. The use of the LCA approach, based on ISO 14040 [8] and ISO 14044 [9], in the livestock sector allows for an understanding of critical issues [10], development of environmental policies [11,12], and specific mitigation actions [13,14,15]. Since 2013, the Product Environmental Footprint (PEF) has been used as a standardized methodology to harmonize LCA studies and assess the environmental impact of products [16]. This method provides specific methodological guidelines for the dairy sector, the Product Environmental Footprint Category Rules (PEFCR), which better understand its complexity [17].

The primary environmental impact indicator associated with milk production is climate change, i.e., the representation of GHGs such as carbon dioxide (CO_2_), methane (CH_4_), and nitrous oxide (N_2_O) [18]. Among GHGs, N_2_O and CH_4_ originate from biological processes in soils, manure, livestock digestive systems, and animal diets [19]. They cannot be calculated with commercial LCA software (SimaPro^®^ v. 7.0) but require specific knowledge of the LCA practitioners. Methane eructation by the animal represents a loss of ingested gross energy (GE), and improved energy efficiency in dairy cows can be achieved with lower CH_4_ production relative to the energy intake or milk production [20]. According to the PEFCR methodology, the assessment of CH_4_ from enteric fermentation and manure management must use the Tier 2 approach suggested by the Intergovernmental Panel on Climate Change (IPCC). Thus, methane production can be estimated by simple regression equations where CH_4_ is linked to GE and by selecting a CH_4_ conversion factor (Y_m_), indicating the proportion of GE converted to enteric CH_4_ [21].

This approach is widely used for national GHG emission inventories. However, the Y_m_-based models generally perform poorly in representing the nutrient content of diets contributing, along with the level of feed intake, to the CH_4_ emission [22]. Therefore, more complex mechanistic and stoichiometric models requiring feed composition and characteristics of provided diets should be used instead. Feed digestibility and dry matter intake (DMI) are the primary determinants of CH_4_ production [23]. Understanding the quality and digestibility of forages fed to dairy cows is essential for estimating enteric CH_4_, as several studies suggest possible mitigation with improved nutrient quality and digestibility [18,23], leading to an increase in feed intake and productivity [24,25]. Near-infrared reflectance spectroscopy (NIRS) analysis can be used to evaluate the nutrient contents of feeds, which can be used for direct or indirect estimates of the animal performance when feeding on a total mixed ration (TMR). Thus, rumen-degradable protein (RDP), rumen-undegradable protein (RUP), and RUP digestibility can be predicted. Also, remarkable similarities were obtained using the in situ-derived protein fractions adopted to estimate RDP, RUP, and RUP digestibility in the most recent Dairy National Research Council model (NRC) and the in vitro, chemically determined protein fractions in the Cornell Net Carbohydrate and Protein System (CNCPS) [26,27]. The NRC provides guidelines for modeling the nutrient requirements to improve the feed efficiency of lactating dairy cows [28], a nutritional system adopted in several countries [27]. The CNCPS is a mathematical and evolving model [29] that estimates cattle requirements and nutrient supply based on animal, environmental, and feed compositional information, which can be adapted to different production situations [29]. Because of the contribution of the livestock sector to GHG emissions, the adoption of mitigation strategies to reduce emissions may be beneficial in tackling climate change.

The excessive presence of ozone (more than 50 µg/m^3^) in the lower troposphere threatens human and animal health as prolonged exposure causes damage to the respiratory tract [30]. Manure handling in dairy farms also contributes to the release of CO_2_, carbon monoxide (CO), sulfur dioxide (SO_2_), nitrogen oxides (NO_x_s), and volatile organic compounds (VOCs), with an average of 3.11 × 10^−7^ kg of CO_2_ per kg of fat- and protein-corrected milk (FPCM) and 9.05 × 10^−8^, 2.71 × 10^−7^, 8.10 × 10^−7^, and 1.92 × 10^−9^ kg of non-methane volatile organic compounds (NMVOC) per kg of FPCM being produced [31], respectively. All these substances affect the relative proportion of the precursors in photochemical ozone creation potentials (POCPs), according to their concentration in the atmosphere. Indeed, a relationship between CH_4_ emissions and POCPs has been reported [32,33,34,35].

The current study aimed to estimate the relationship between diet digestibility, milk yield, and their environmental impact on CC and POCP indicators. A comparison between different estimates of digestible energy (DE) was proposed, and the IPCC guidelines were used to obtain reference values. DE was proposed within a range for each animal category, and GE was determined as a sum of the animals’ energy requirements. The animal’s GE intake and DE of the diet were based on the quality of feeds used and were evaluated with NIR analysis. The diets provided to each animal category were evaluated through two feeding formulation models: NRC and CNCPS. The results on impact indicators were analyzed among herds’ performance and compared against the estimates obtained according to the IPCC guidelines.

## 2. Materials and Methods

### 2.1. Life Cycle Inventory (LCI)

Fifty-five dairy farms rearing mainly Holstein Friesian cattle were investigated (Table 1), as reported in Froldi et al. [36]. Farms were specialized in producing milk for PDO cheese making, and silages (mainly from corn) were used when preparing diets. Primary data were collected using a planned questionnaire completed on the farm, which was carried out according to the European Dairy Association [37]. The collected activity data for the LCI were related to the supplies used in farm activities for producing the in-farm feeds (such as chemical fertilizers, pesticides, energy, and water) for purchasing feeds and breeding activity (Table 1). The milk and co-product (meat) amounts were also included in the LCI. Secondary data integrated missing information with primary data.

### 2.2. Functional Units

One kg FPCM at the farm gate was considered a functional unit (FU). The FU did not consider milk processing (i.e., transport, heating, and cooking). FPCM was calculated according to the International Dairy Federation [38], as follows:

(1) $$\begin{array}{ll} FPCM\left(\frac{kg}{y}\right) & =raw\;milk\left(\frac{kg}{y}\right)\\ & \;\times \left(0.1226\times fat\;content\;\%+0.0776\times true\;protein\;\%+0.2534\right) \end{array}$$

Equation (1) applied primary data for raw milk yield and milk quality records for fat and true protein contents.

### 2.3. System Boundary

The system boundary included all farm activities related to milk production, crop production, and feed purchase on the market. The inputs and outputs considered the PEF methodology. Primary activity data regarding inputs (deliveries and consumables) and outputs (main product, co-products, emissions, and manure) were collected and organized in a specific life cycle inventory (LCI) (Table 1). Dairy farms were classified according to the average milk yield into three groups via a quartile distribution, as explained later. The emission modeling of enteric fermentation and manure management followed the PEFCR methodology and alternative models (Table 2).

### 2.4. Allocation

The allocation followed a bio-physical approach, considering milk as the main product and meat as a co-product [17,38]. The manure leaving the farm without an economic allocation was not considered a co-product [38]. In addition, feeds produced and sold were also excluded from the environmental assessment, subtracting the necessary inputs for their production from the LCI (i.e., diesel, pesticides, and chemicals).

### 2.5. Impact Assessment

The environmental impact of the FU was expressed for the CC [46] and POCP [47] indicators. The estimates were obtained using SimaPro^®^ software v9.0.0.35 [48], following the PEF method version 2.0. The considered emissions and reference methods for the analysis of the LCI are reported in Table 2.

### 2.6. Direct CH_4_ Emissions Estimate

To consider the effect of the quality of feeds used in the formulation of a diet on DE and CH_4_ emissions from enteric fermentation and manure, all in-farm produced feeds were sampled: silage fodder (corn, sorghum, and wheat), forages (alfalfa, cereal mix, ryegrass, and meadow hay), and cereal meals (corn, wheat, and barley). In addition, samples of TMR were also collected for each of the considered animal categories: lactating cows, dry cows, heifers (from 12 months of age to first calving), young heifers (from weaning to 12 months of age), and calves (from birth until weaning). Dry matter was measured after the samples were dried in a ventilated oven at 65 °C for 48 h [49]. Then, the dried samples were ground (Fritsch Pulverisette 19 mill) with a 0.5 mm sieve and stored before being analyzed using the near-infrared reflectance technique (FOSS NIR-System 5000, Silver Spring, MD, USA). A commercial feed analysis laboratory provided the NIR system calibration curves for feed and TMR analysis (CRPA Lab, Reggio Emilia, Italy). The nutrient contents of feeds from NIR measurements were used when evaluating the diet (Razio-Best v560, Piacenza, Italy), whereas the analytical components reported on the illustrative labels collected in each dairy farm (when available) were used for concentrates as well as mineral and vitamin supplements. In addition, standard concentrate formulations (one for each category of animals) were remodeled (Razio-Best v560). The nutritional characteristics of purchased feeds were referenced to tabulated values. The following components of feeds were considered: dry matter, crude protein, fats, ash, starch, sugars, neutral detergent fiber (NDF), acid detergent fiber (ADF), and acid detergent lignin (ADL). Structural data of the animal system considered the number of animals in each category and average live weight, days in lactation, milk yield and quality (fat and protein contents), housing, feeding technique used, use of silages and forage quality, and season of reference.

#### 2.6.1. Feeding Model Approach

**Figure 1:**
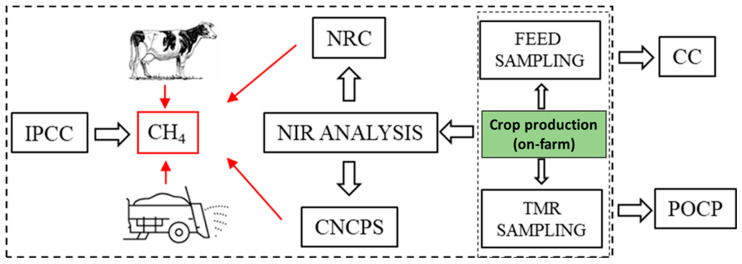
Feeding model approach scheme used for the evaluation of CH_4_ emissions.

The nutrient requirements were evaluated according to the NRC and CNCPS models (Figure 1). The approach of a diet evaluation based on evaluated feed composition instead of tabulated values allowed for tuning the estimate of expected Ge intake, DE of the diet being fed, energy in urine (UE), ash (ASH in manure), and total volatile solids (VSs) of manure. Estimates will be used in place of IPCC proposed values (2019) for a more detailed assessment of CH_4_ production (Table 2).

#### 2.6.2. Direct CH_4_ Emission Models

VS excretion was calculated according to the IPCC guidelines [39] (Equation (10.24)) and for each considered animal category (Equation (2)):

(2) $$VS=\left[GE\times \left(1-\frac{DE}{100}\right)+(UE\times GE)\right]\times \left[\left(\frac{1-ASH}{18.45}\right)\right]$$

where

VS = volatile solid excretion per day based on a dry-organic matter, kg VS day^−1^;
GE = gross energy intake, MJ day^−1^;
DE = digestible energy expressed as percent of GE;
(UE × GE) = urinary energy used as a fraction of GE;
ASH = ash content calculated as a fraction of the DM feed intake;
18.45 = conversion factor used for dietary GE per kg of DM (MJ kg^−1^).

The GE, DE, and ASH values were obtained from the formulation software when diets were evaluated according to either the NRC or the CNCPS. For the IPCC approach, an ASH value of 0.08 was used for each category of animals, whereas GE intake was calculated according to guidelines [39] (Equation (10.16)) as the sum of net energy requirements and considering the DE of the fed diet as a percentage of GE intake: 87% for calves, 63% for young heifers, 62% for heifers and dry cows, and 66% for lactating cows.

The CH_4_ emission factor [39] (Equation (10.21)) from enteric fermentation from a livestock category was obtained as follows (Equation (3)):

(3) $$EF=\frac{GE\times \left(\frac{Y_{m}}{100}\right)\times 365}{55.65}$$

where

EF = emission factor, kg CH_4_ head^−1^ yr^−1^;
GE = gross energy intake, MJ head^−1^ yr^−1^;
Y_m_ = the CH_4_ conversion factor, expressed as a percent of GE intake converted to CH_4_. The factor was 55.65 (MJ/kg CH_4_) (i.e., the energy content of CH_4_)

The CH_4_ conversion factor (Y_m_) was 0, 6.5, and 6.3, respectively, for calves, young heifers/heifers/dry cows, and lactating cows [39].

The CH_4_ emission factor [39] (Equation (10.23)) for manure management was obtained as follows (Equation (4)):

(4) $${EF}_{\left(T\right)}=({VS}_{\left(T\right)}\times 365)\times \left[B_{0\left(T\right)}\times 0.67\times \sum_{\left(S,k\right)}\frac{{MCF}_{\left(S,k\right)}}{100}\times {AWMS}_{\left(T,S,k\right)}\right]$$

where

EF_(T)_ = annual CH_4_ emission factor from manure for T, kg CH_4_ animal^−1^ yr^−1^;
VS_(T)_ = daily volatile solid excreted, referred to as T, kg dry matter animal^−1^ day^−1^;
365 = basis days for calculating annual VS production, days yr^−1^;
B_0(T)_ = maximum CH_4_ producing capacity used for manure for T, m^3^ CH_4_ kg^−1^ of VS excreted;
0.67 = conversion factor of m^3^ CH_4_ to kilograms CH_4_;
MCF_(S,k)_ = the CH_4_ conversion factors for manure management system S expressed by climate region k, percent;
AWMS_(T,S,k)_ = fraction of T’s manure handled using animal waste management (i.e., manure) system S in climate region k, dimensionless.

The emission factor from manure management considered a B_0(T)_ of 0.24 m^3^ CH_4_ kg^−1^ of VSs excreted, manure management systems, and CH_4_ conversion factors (MCF) [39] (Annex 10A3).

### 2.7. Statistical Approach

Farms were grouped based on the average milk yield/cow/d [36] and identified as high-, mid-, and low-performing herds according to the quartile distribution (>32.6; 25.4–32.6; <25.4 kg milk/cow/d, respectively). Data analysis aimed at CC and POCP impact indicator estimates between groups of herds.

A linear additive model was used to analyze the data (Equation (5)):

(5) $$Y_{ij}=\mu +\alpha_{i}+\varepsilon_{ij}$$

where Y_ij_ is the experimental data, μ is the overall mean, α_i_ is the fixed effect of the group (i = 1–2 and 3), and ε_ij_ is the random error.

Data were tested for normality with the Shapiro–Wilk test before statistical analysis. Tukey’s honestly significant difference test was used for comparing the means of normally distributed data. When the assumption for the normality of the data distribution was violated, the Steel–Dwass all-pairs test was performed for a non-parametric analysis of pairwise rankings in the presence of unequal sample sizes [50]. A paired statistical analysis was used to evaluate the effect of implementing different approaches for estimating DE [39]. All statistical analyses were conducted using JMP^®^ Pro 17.0.0 [51], and means were considered different at *p* < 0.05.

## 3. Results

### 3.1. Characterization Results of IPPC Approach

The environmental impacts for the CC and POCP indicators were expressed based on 1 kg of FPCM and are reported in Table 3. Impacts were modeled according to IPCC [39] and reported per group of herds either as a total impact or itemized into eight main categories of emissions: feed purchase, in-farm feeds, in-farm water use, energy, enteric fermentation, barn management, bedding materials, and manure handling. Groups of herds were found to have differences in both considered impact indicators. High-performing herds had a lower (*p* < 0.05) CC impact (1.63 kg CO_2_-eq) than mid- (1.93 kg CO_2_-eq) or low-performing herds (2.17 kg CO_2_-eq). The pattern of the differences was maintained (*p* < 0.05) for enteric fermentation, the major contributor to CC, with values of 6.3 × 10^−1^, 7.11 × 10^−1^, and 8.14 × 10^−1^ kg CO_2_-eq, respectively, for high-, mid-, and low-performing herds as well as and for in-farm feeds, energy, and in-farm water use categories of emissions. High-performing herds reported lower results (*p* < 0.05) with regard to CC (8.26 × 10^−3^ kg CO_2_-eq) when referring to the bedding materials category than low-performing herds (2.84 × 10^−2^ kg CO_2_-eq). No differences among groups were observed for the second (feed purchase) and third (manure handling) contributors to CC, averaging 5.76 × 10^−1^ and 4.27 × 10^−1^ kg CO_2_-eq, respectively. Regarding their contribution, the following factors were listed in order of importance and how much of an impact they had on CC: biogenic sources (CC-biogenic), fossils (CC-fossil), and land and use transformations (CC-LTU). Despite having a higher CC-biogenic share, the high-performing herds had a 16.7% lower impact of the indicator (9.71 × 10^−1^ kgCO_2_-eq; *p* < 0.05) compared with mid- and low-performing herds (on average 1.17 kgCO_2_-eq). The main contribution was from enteric fermentation with lower (*p* < 0.05) values for high- (6.3 × 10^−1^ kg CO_2_-eq) compared with mid- (7.11 × 10^−1^ kg CO_2_-eq) and low-performing herds (8.14 × 10^−1^ kg CO_2_-eq), whereas the remainder was almost all from manure handling, with similar values among herds (3.66 × 10^−1^ kg CO_2_-eq). The high-performing herds were also lower (*p* < 0.05) in CC-fossil (4.20 × 10^−1^ kg CO_2_-eq) compared with mid- and low-performing herds (on average 5.61 × 10^−1^ kg CO_2_-eq). The main emission category was feed purchase (52.3%), with similar values among herds (2.64 × 10^−1^ kg CO_2_-eq), followed by in-farm feeds, energy, and manure handling with lower (*p* < 0.05) values for high- compared with low-performing herds. CC-LTU was lower (*p* < 0.05) in high- and mid-performing herds, averaging 2.64 × 10^−1^ kg CO_2_-eq), compared with low-performing herds (3.66 × 10^−1^ kg CO_2_-eq). The impact on the POCP indicator was different (*p* < 0.05) among the groups of herds, being higher in low-performing herds, with barn management, energy, and feed purchase categories accounting for 75.2%, 13.5%, and 6.8% of the emissions (1.12 × 10^−2^ kg CO_2_-eq), respectively. As we move toward less impactful herds on POCP indicators, mid- (8.65 × 10^−3^ kg CO_2_-eq) and high- (6.88 × 10^−3^ kg CO_2_-eq) performing herds, when the barn management category’s contribution is equal, the contribution of energy becomes reduced while feed purchase increases.

### 3.2. Characterization Results of NRC and CNCPS Approach

Table 4 and Table 5 show how different approaches in estimating diet digestibility affect the CC and POCP impact indicators. Results are reported from a paired analysis using the IPCC approach as the reference method and considering the affected emission categories: enteric fermentation and manure handling. The IPCC overestimates (*p* < 0.05) the impacts on indicators in mid- and high-performing herds, independently of the alternative approach used for DE attribution. The pattern of results was confirmed for the enteric fermentation category, whereas in the manure handling category, low-performing herds were also overestimated using the IPCC approach (Table 5).

## 4. Discussion

The average CC value for producing 1 kg of FPCM was higher (from 9 to 32%) than the previously reported studies conducted in the same geographical area of Northern Italy [52,53,54,55,56,57]. Despite a similar ratio of dairy cows and growing animals for a replacement within herds, the higher CC value of low- versus high-performing herds resulted from lower milk yield. The difference in CC estimates could be even higher considering the lower allocation of milk for the low-performing herds, a low allocation that could result from a higher culling rate, either for low milk yield or health-related problems.

The land available for dairy cows decreased when increasing the herd’s performance. Indeed, high-performing herds were more demanding in the market for DM fed to animals, whereas low-performing herds seemed to rely more on self-produced crops. When properly managed, the latter should allow for higher-quality feeds. However, low-performing herds were also characterized by a higher share of less digestible feeds, like polyphyte hay, and proteic feed, like soya, with an increased CC impact. On the other hand, high-performing herds accessed the market instead for alfalfa, a more valuable roughage than polyphyte hay, and for compound feeds to support and meet the nutritional requirements of animals, resulting in a higher milk yield and quality, leading to a lower CC impact per unit of milk produced. The low purchase of compound feeds in low-performing herds was due to a higher tendency to use raw materials in animal feeding, as also noted by Dewhurst et al. [58]. Low-performing herds were also characterized by a higher use of a chemical fertilizer, water, and diesel to support in-farm feeds. There may be several reasons for a low efficiency in utilizing livestock resources. Dependence on a nitrogen chemical fertilizer is increased due to the lower capacity of valorizing livestock manure [59]. According to a study by Thompson et al. [60], sprinkling and flooding irrigation methods consume more water than drip irrigation. The lack of technologically advanced irrigation systems in low-performing herds did not allow for reduced water usage in comparison to the high- and mid-performing herds, where access to credits could allow for more precise economic investments [61]. Low-performing herds double the purchase of bedding materials, mainly cereal straw, when compared with high-performing herds, even though they comprise about 70% of the herds’ size; the reasons could be addressed by employing a higher presence of simpler housing systems involving the use of litter. Nevertheless, while simpler animal housings were present in low-performing herds, the management system of such solutions is more demanding in terms of cleaning and labor activities [62].

Mid-performing herds were typically larger and relied heavily on feed purchases, of which 46% were compound feeds, with a lower share of soy. This particular feed flow entering the farm could penalize the nutrient quality supporting milk yield. Nonetheless, a considerable amount of available land was used for grass growth, which suggests a more considerable use of chemical fertilizers in these farms. These herds also used higher amounts of water for irrigation and diesel for crop cultivation, similar to the low-performing herds. As a result, data suggest that in front of a larger size of available land supporting a larger herd’s size, the flow of feeds was probably lacking in terms of nutrient quality, leading to a lower milk yield and, therefore, to a larger CC impact compared with high-performing herds.

Indeed, low- and mid-performing herds were ranked higher for their CC impact from feed purchase, enteric fermentation, and manure handling, which are all related to the quality of feeds used in the diets provided to the animals.

The approaches used in modeling primary data for estimating CH_4_ from enteric fermentation and manure handling implemented the guidelines proposed by the 2019 IPCC update [39]. Thus, the MCF was obtained using detailed information about the number of times manure storages were emptied per year and the monthly temperature profiles for each dairy farm, as opposed to the default MCF values from previous IPCC guidelines [42].

The recent Global Methane Pledge of the European Union aims for a 30% reduction in anthropogenic CH_4_ by 2030 compared to the 2020 levels [63]. Thus, an accurate estimate of CH_4_ production from enteric fermentation and manure handling is necessary [64].

The approach used to evaluate the animal’s nutrient requirements affects the formulation of the diet and, combined with the nutrient contents of feeds entering the diet, yields different estimates of GE intake and DE supplied with the diet. An accurate estimation of GE and DE is crucial for reducing methane emissions from undigested feeds in manure. According to Rotz et al. [65] and Hristov et al. [23], methane emissions are influenced by feed composition, energy content, and intake. Therefore, properly balanced diet formulations can reduce CH_4_ emissions. In this context, Dourmad et al. [66] and Yan et al. [20] suggested that increasing the feed energy value could reduce CH_4_ emissions and impact on CC and POCP indicators. In this regard, Ravishankara et al. [63] found a decrease in CH_4_ emissions due to increased DE in grazing animals, resulting in a 2–6% reduction in POCPs. Although photochemical ozone formation is of concern, the livestock sector has a low impact compared to other anthropogenic activities [67], and its estimation results are complex [44]. As previously reported [68], in addition to CH_4_, the POCPs are VOC emissions that may originate from animal manure and fermented feeds (i.e., silage feed). Indeed, a direct measurement would be necessary to identify the source(s) of the emissions [58]. In the beef supply chain, Putman et al. [69] stated that about 10–15% of NMVOC emissions come from silage feeding [70] and 85–90% from diesel and energy sources. However, these emissions could be controlled or reduced through proper forage production, silage operations, and feeding and storage management [71]. This could explain the results obtained in this research: a better quality of in-farm feeds, including the silage estimated using NIR analysis, leads to higher feed digestibility (estimated with NRC or CNCPS), resulting in lower CC and POCP impacts in high- and mid-performing herds. Therefore, in similar LCA studies, higher feed quality could lead to an increase in milk yields [72], with the latter leading to a decrease in the severity of the impact per kg of milk being produced [73,74,75]. The estimate of GE intake also relies on the knowledge of BW of different categories of reared animals [28,29]. The latter represents a weakness of this study if not properly addressed. Only a few commercial farms feature scales for monitoring animal BW, and they are usually located at the exit of the milking parlor and, therefore, are limited to lactating animals. If a direct measurement is not possible, animal BW can be obtained through on-farm surveys or by relying on the data collected from the breeder association’s monthly milk quality check. Of the farms where BW was measured in lactating dairy cows (24% of surveyed farms), the BW reading was about 3% higher than the estimated value. No direct measurements of BW in animal categories other than lactating dairy cows are usually available on-farm. Further research is needed to achieve a direct measurement of BW in animals.

## 5. Conclusions

This study aimed to investigate the effect of herds’ performance and how GE and DE estimates might influence the environmental impact of milk estimated using CC and POCP indicators. The CC and POCP impact indicators were affected by the herds’ performance. As herd performance increased, a lower impact was observed on these indicators. High-performing herds had lower contributions to CC from enteric fermentation and CC-LTU.

Implementing the IPCC guidelines without considering the quality of feeds and, therefore, an estimate of GE and DE could lead to overestimating the CC and POCP indicators, particularly in mid- and high-performing herds.

Results are of paramount relevance since feed quality and diet digestibility are peculiar to each farm and might deviate too much from the default values suggested by the IPCC guidelines.

Additional efforts should be made to model the CH_4_ produced through enteric fermentation. Currently, no algorithms consider the relationship between changes in diets or feed compositions, changes in digestible energy, and the estimation of enteric CH_4_ emissions. Therefore, developing models that can accurately estimate CH_4_ emissions from enteric fermentation in livestock is crucial.

## Figures and Tables

**Table 1:** Life cycle inventory of the analysis groups of dairy farms.

				Dairy Farm Performance			
		High ^1^		Mid ^1^		Low ^1^	
Inventory	Units	Mean	±SD	Mean	±SD	Mean	±SD
Dairy farm data							
Farms	n.	14		27		14	
Dairy cows	cows farm^−1^ year^−1^	142	79	177	192	103	71
Dry cows	dry cows farm^−1^ year^−1^	23	15	26	28	15	11
Heifers	heifers farm^−1^ year^−1^	66	36	88	98	49	42
Other animals ^2^	n. animals^−1^ year^−1^	70	36	89	89	51	40
Land use	ha	49.8	26	69.3	110	49.9	39
Average milk production	t FPCM farm^−1^ year^−1^	1959.1	952.4	1913.6	2116.6	827.9	642.8
Average meat production	t meat farm^−1^ year^−1^	33.9	20.3	39.3	46.1	18.4	12.8
Raw milk allocation	%	90.0	3	88.0	3	85.0	5
In-farm inputs							
Electricity	kWh year^−1^	180,783	198,901	259,297	233,112	126,998	99,272
Diesel	lt year^−1^	18,000	16,048	28,018	32,181	19,021	19,752
Water used on farm	m^3^ year^−1^	6401	6230	9827	10,462	5761	4283
Irrigation water	m^3^ year^−1^	99,469	96,861	180,927	285,856	107,967	110,925
Chemical fertilizers	kg N year^−1^	2422	2842	5872	10,295	3217	5637
Pesticides ^3^	kg year^−1^	154	158	202	315	157	173
Bedding materials ^4^	kg DM year^−1^	71,510	77,508	52,077	49,670	107,461	176,025
Off-farm feed							
Alfalfa	kg DM year^−1^	62,824	100,231	49,168	49,683	13,876	20,952
Polyphyte hay	kg DM year^−1^	14,040	27,185	36,487	46,232	38,602	106,253
Corn ^5^	kg DM year^−1^	141,917	176,661	213,130	193,104	83,396	89,279
Cereals ^6^	kg DM year^−1^	28,899	74,387	12,619	37,093	325	1215
Soya	kg DM year^−1^	67,553	123,936	55,714	109,121	84,150	87,294
Sunflower	kg DM year^−1^	-	-	13,165	45,541	9051	14,488
Cotton	kg DM year^−1^	6962	19,222	9458	29,200	-	-
Compound feed	kg DM year^−1^	132,478	192,934	333,670	634,575	65,112	38,910

^1^ High-, mid-, and low-performing herds: >32.6; 25.4–32.6; <25.4 kg milk/cow/d; respectively. ^2^ Young heifers from weaning to 12 months of age; calves from birth until weaning. ^3^ Fungicides, herbicides, and insecticides. ^4^ Cereal straw, lime, calcium carbonate, corn stalks, coconut fiber, sawdust, and woodchips. ^5^ Corn meal, corn silage, corn flakes, wholemeal corn mash, and corn grain. ^6^ Wheat silage, wheat meal, barley meal, and sorghum silage.

**Table 2:** Emissions estimated and methods used for CC and POCP impact categories.

Emissions	Methodology Used
CH_4_—enteric fermentation	[39]
CH_4_—storage and pre-treatment	[39,40,41]
N_2_O—direct: manure storage and field fertilization	It includes the excretion of liquid and solid manure on pasture [39,40,41].
N_2_O—direct and indirect: mineral fertilizers application	[42]
N_2_O—indirect: manure spreading	It includes the manure spreading on the fields and the pasture (considered the emissions from N leaching) [39].
N_2_O—indirect: manure and field fertilization	It includes the excretion of liquid and solid manure on pasture; it is considered N volatilization (NH_3_ and NO_x_) [39,40,41].
PM_2.5_—animal housing	[40,41,43]
NMVOC—animal housing, manure storage, and animal grazing	[40,41,44]
NMVOC—manure spreading on field	[44]
NMVOC—silage storage and usage	[44]
CO_2_—urea fertilization	[45]

**Table 3:** Characterization results for 1 kg of FPCM produced by farms for considered impact indicators modelled according to the IPCC approach (please refer to Table S1 for detailed statistical analysis).

						Category of Emission				
Impact Indicator	Herds ^1^	Total	Feed Purchase	In-Farm Feeds	In-Farm Water Use	Energy	Enteric Fermentation	Barn Management	Bedding Materials	Manure Handling
CC kgCO_2_-eq.	High-performing	1.63 ^c^	4.97 × 10^−1 a^	5.91 × 10^−2 c^	1.30 × 10^−3 c^	5.82 × 10^−2 c^	6.30 × 10^−1 c^	0	8.26 × 10^−3 b^	3.77 × 10^−1 a^
	Mid-performing	1.93 ^b^	5.92 × 10^−1 a^	8.74 × 10^−2 b^	1.67 × 10^−3 b^	7.96 × 10^−2 b^	7.11 × 10^−1 b^	0	1.22 × 10^−2 ab^	4.46 × 10^−1 a^
	Low-Performing	2.17 ^a^	6.40 × 10^−1 a^	1.16 × 10^−1 a^	2.17 × 10^−3 a^	1.16 × 10^−1 a^	8.14 × 10^−1 a^	0	2.84 × 10^−2 a^	4.58 × 10^−1 a^
	SE	6.10 × 10^−2^	4.61 × 10^−2^	8.89 × 10^−3^	6.16 × 10^−5^	6.34 × 10^−3^	1.60 × 10^−2^	-	3.94 × 10^−3^	3.23 × 10^−2^
CC-biogenic kgCO_2_-eq.	High-performing	9.71 × 10^−1 b^	9.63 × 10^−3 a^	3.37 × 10^−6 b^	4.40 × 10^−6 c^	7.14 × 10^−5 c^	6.30 × 10^−1 c^	0	1.64 × 10^−6 b^	3.31 × 10^−1 a^
	Mid-performing	1.11 ^a^	1.07 × 10^−2 a^	7.54 × 10^−6 b^	5.65 × 10^−6 b^	1.03 × 10^−4 b^	7.11 × 10^−1 b^	0	2.64 × 10^−6 ab^	3.80 × 10^−1 a^
	Low-Performing	1.22 ^a^	2.34 × 10^−2 a^	1.09 × 10^−5 a^	7.35 × 10^−6 a^	1.62 × 10^−4 a^	8.14 × 10^−1 a^	0	6.76 × 10^−6 a^	3.87 × 10^−1 a^
	SE	3.90 × 10^−2^	4.83 × 10^−3^	2.08 × 10^−6^	2.08 × 10^−7^	1.03 × 10^−5^	1.60 × 10^−2^	-	1.06 × 10^−6^	3.21 × 10^−2^
CC-fossil kgCO_2_-eq.	High-performing	4.20 × 10^−1 b^	2.48 × 10^−1 a^	5.91 × 10^−2 c^	1.30 × 10^−3 c^	5.79 × 10^−2 c^	0	0	8.15 × 10^−3 b^	4.54 × 10^−2 c^
	Mid-performing	5.31 × 10^−1 a^	2.92 × 10^−1 a^	8.73 × 10^−2 b^	1.66 × 10^−3 b^	7.91 × 10^−2 b^	0	0	1.21 × 10^−2 ab^	5.87 × 10^−2 b^
	Low-Performing	5.90 × 10^−1 a^	2.52 × 10^−1 a^	1.16 × 10^−1 a^	2.16 × 10^−3 a^	1.15 × 10^−1 a^	0	0	2.75 × 10^−2 a^	7.75 × 10^−2 a^
	SE	2.96 × 10^−2^	3.05 × 10^−2^	8.88 × 10^−3^	6.13 × 10^−5^	6.30 × 10^−3^	-	-	3.77 × 10^−3^	2.06 × 10^−3^
CC-LTU kgCO_2_-eq.	High-performing	2.39 × 10^−1 b^	2.39 × 10^−1 b^	1.89 × 10^−5 b^	1.00 × 10^−6 c^	2.76 × 10^−4 c^	0	0	1.02 × 10^−4 a^	0
	Mid-performing	2.89 × 10^−1 ab^	2.89 × 10^−1 ab^	2.71 × 10^−5 b^	1.28 × 10^−6 b^	4.04 × 10^−4 b^	0	0	2.63 × 10^−5 a^	0
	Low-Performing	3.66 × 10^−1 a^	3.64 × 10^−1 a^	4.80 × 10^−5 a^	1.67 × 10^−6 a^	6.40 × 10^−4 a^	0	0	8.73 × 10^−4 a^	0
	SE	2.66 × 10^−2^	2.66 × 10^−2^	5.99 × 10^−6^	4.74 × 10^−8^	4.18 × 10^−5^	-	-	2.73 × 10^−4^	-
POCP kgNMVOC-eq.	High-performing	6.88 × 10^−3 c^	7.27 × 10^−4 a^	1.07 × 10^−5 b^	3.56 × 10^−6 c^	6.58 × 10^−4 c^	1.87 × 10^−4 c^	5.18 × 10^−3 c^	2.20 × 10^−5 b^	9.85 × 10^−5 a^
	Mid-performing	8.65 × 10^−3 b^	8.63 × 10^−4 a^	2.74 × 10^−5 b^	4.56 × 10^−6 b^	9.57 × 10^−4 b^	2.11 × 10^−4 b^	6.43 × 10^−3 b^	3.38 × 10^−5 ab^	1.15 × 10^−4 a^
	Low-Performing	1.12 × 10^−2 a^	7.63 × 10^−4 a^	3.85 × 10^−5 a^	5.94 × 10^−6 a^	1.51 × 10^−3 a^	2.42 × 10^−4 a^	8.42 × 10^−3 a^	7.54 × 10^−5 a^	1.13 × 10^−4 a^
	SE	2.99 × 10^−4^	8.98 × 10^−5^	8.59 × 10^−6^	1.68 × 10^−7^	9.69 × 10^−5^	4.75 × 10^−6^	2.64 × 10^−4^	1.04 × 10^−5^	9.53 × 10^−6^

^1^ High-performing herds, mid-performing herds, and low-performing herds (>32.6; 25.4–32.6; <25.4 kg milk/cow/d, respectively). ^abc^ Means without a common superscript within a column and impact indicator differ (*p* < 0.05).

**Table 4:** Characterization results for 1 kg of FPCM produced by farms for considered impact indicators: difference between NRC and IPCC approaches (please refer to Table S2 for detailed statistical analysis).

			Category of Emission	
Impact Indicator	Herds ^1^	Total	Manure Handling	Enteric Fermentation
CC kgCO_2_-eq.	High-performing	−1.42 × 10^−1^ *	−4.14 × 10^−2^ *	−1.00 × 10^−1^ *
	Mid-performing	−6.18 × 10^−2^ *	−1.83 × 10^−2^ *	−4.35 × 10^−2^ *
	Low-performing	2.39 × 10^−2^	9.17 × 10^−3^	1.47 × 10^−2^
POCP, kg NMVOC-eq.	High-performing	−4.21 × 10^−5^ *	−1.23 × 10^−5^ *	−2.98 × 10^−5^ *
	Mid-performing	−1.84 × 10^−5^ *	−5.40 × 10^−6^ *	−1.29 × 10^−5^ *
	Low-performing	7.10 × 10^−6^	2.70 × 10^−6^	4.40 × 10^−6^

^1^ High-performing herds, mid-performing herds, and low-performing herds (>32.6; 25.4–32.6; <25.4 kg milk/cow/d, respectively). * Different from zero (*p* < 0.05).

**Table 5:** Characterization results for 1 kg of FPCM produced by farms for considered impact indicators: difference between CNCPS and IPCC approaches (please refer to Table S3 for detailed statistical analysis).

			Category of Emission	
Impact Indicator	Herds ^1^	Total	Manure Handling	Enteric Fermentation
CCC, kgCO_2_-eq.	High-performing	−1.81 × 10^−1^ *	−8.08 × 10^−2^ *	−1.00 × 10^−1^ *
	Mid-performing	−1.19 × 10^−1^ *	−7.58 × 10^−2^ *	−4.35 × 10^−2^ *
	Low-performing	−2.39 × 10^−2^	−3.87 × 10^−2^ *	1.47 × 10^−2^
POCP, kg NMVOC-eq.	High-performing	−5.38 × 10^−5^ *	−2.40 × 10^−5^ *	−2.98 × 10^−5^ *
	Mid-performing	−3.54 × 10^−5^ *	−2.25 × 10^−5^ *	−1.29 × 10^−5^ *
	Low-performing	−7.10 × 10^−6^	−1.15 × 10^−5^ *	4.40 × 10^−6^

^1^ High-performing herds, mid-performing herds, and low-performing herds (>32.6; 25.4–32.6; <25.4 kg milk/cow/d, respectively). * Different from zero (*p* < 0.05).

## Data Availability

Data are contained within this article.

## Supplementary Materials

The following supporting information can be downloaded at: https://www.mdpi.com/article/10.3390/ani14121725/s1, Table S1: supplementary material for Table 3; Table S2: supplementary material for Table 4; Table S3: supplementary material for Table 5.

## References

[1] Üçtuğ F.G. (2019). The environmental life cycle assessment of dairy products. Food Eng. Rev..

[2] Pulina G., Lunesu M.F., Pirlo G., Ellies-Oury M.P., Chriki S., Hocquette J.F. (2022). Sustainable production and consumption of animal products. Curr. Opin. Environ. Sci. Health.

[3] Bava L., Sandrucci A., Zucali M., Guerci M., Tamburini A. (2014). How can farming intensification affect the environmental impact of milk production?. J. Dairy Sci..

[4] Rial-Lovera K., Davies P., Cannon N.D. (2017). Implications of climate change predictions for UK cropping and prospects for possible mitigation: A review of challenges and potential responses. J. Sci. Food Agric..

[5] Arrigoni A., Marveggio D., Allievi F., Dotelli G., Scaccabarozzi G. (2023). Environmental and health-related external costs of meat consumption in Italy: Estimations and recommendations through life cycle assessment. Sci. Total Environ..

[6] Notarnicola B., Sala S., Anton A., McLaren S.J., Saouter E., Sonesson U. (2017). The role of life cycle assessment in supporting sustainable agri-food systems: A review of the challenges. J. Clean. Prod..

[7] FAO (2016). Environmental performance of large ruminant supply chains: Guidelines for assessment. Livestock Environmental Assessment and Performance Partnership.

[8] (2006). Environmental Management—Life Cycle Assessment—Principles and Framework.

[9] (2006). Environmental Management—Life Cycle Assessment—Requirements and Guidelines.

[10] Rotz A., Stout R., Leytem A., Feyereisen G., Waldrip H., Thoma G., Holly M., Bjorneberg D., Baker J., Vadas P. (2021). Environmental assessment of United States dairy farms. J. Clean. Prod..

[11] Sala S., Martino A., Antoine A., Fulvio B. (2021). The evolution of life cycle assessment in European policies over three decades. Int. J. Life Cycle Assess..

[12] Visentin C., da Trentin A.W.S., Braun A.B., Thomé A. (2020). Life cycle sustainability assessment: A systematic literature review through the application perspective, indicators, and methodologies. J. Clean. Prod..

[13] Lu Y., Ma L., Ma W., Shao L. (2024). Strategies to mitigate the environmental footprints of meat, egg and milk production in northern China. J. Clean. Prod..

[14] Rencricca G., Froldi F., Moschini M., Trevisan M., Lamastra L. (2023). Mitigation Actions Scenarios Applied to the Dairy Farm Management Systems. Foods.

[15] Tullo E., Finzi A., Guarino M. (2019). Review: Environmental Impact of Livestock Farming and Precision Livestock Farming as a Mitigation Strategy. Sci. Total Environ..

[16] Frasnetti E., Ravaglia D’Ammaro P.D., Capri E., Lamastra L. (2023). Can Italian wines outperform European benchmarks in environmental impact? An examination through the product environmental footprint method. Sci. Total Environ..

[17] The European Dairy Association (EDA) (2018). Product Environmental Footprint Category Rules for Dairy Products. https://ec.europa.eu/environment/eussd/smgp/PEFCR_OEFSR_en.html.

[18] Van Soest P.J. (1994). Nutritional Ecology of the Ruminant.

[19] Henriksson M., Cederberg C., Swensson C. (2014). Carbon footprint and land requirement for dairy herd rations: Impacts of feed production practices and regional climate variations. Animal.

[20] Yan T., Agnew R., Gordon F., Porter M. (2000). Prediction of methane energy output in dairy and beef cattle offered grass silage-based diets. Livest. Prod. Sci..

[21] Patel M., Wredle E., Börjesson G., Danielsson R., Iwaasa A.D., Spörndly E., Bertilsson J. (2011). Enteric methane emissions from dairy cows fed different proportions of highly digestible grass silage, Acta Agriculturae Scandinavica, Section A. Anim. Sci..

[22] Ellis J.L., Bannink A., France J., Kebreab E., Dijkstra J. (2010). Evaluation of enteric methane prediction equations for dairy cows used in whole farm models: Methane Prediction In Vivo Farm Models. Glob. Change Biol..

[23] Hristov A.N., Oh J., Lee C., Meinen R., Montes F., Ott T., Firkins J., Rotz A., Dell C., Adesogan A., Gerber P.J., Henderson B., Makkar H.P.S. (2013). Mitigation of Greenhouse Gas Emissions in Livestock Production—A Review of Technical Options for Non-CO_2_ Emissions.

[24] Allen M.S. (2000). Effects of diet on short-term regulation of feed intake by lactating dairy cattle. J. Dairy Sci..

[25] Mertens D.R., Fahey G.C., Collins M., Mertens D.R. (1994). Regulation of forage intake. Forage Quality, Evaluation, and Utilization.

[26] Niu M., Kebreab E., Hristov A.N., Oh J., Arndt C., Bannink A., Bayat A.R., Brito A.F., Boland T., Casper D. (2018). Prediction of enteric methane production, yield, and intensity in dairy cattle using an intercontinental database. Glob. Change Biol..

[27] Schwab C.G., Tylutki T.P., Ordway R.S., Sheaffer C., Stern M.D. (2003). Characterization of Proteins in Feeds. J. Dairy Sci..

[28] NRC (2001). Nutrient Requirements of Dairy Cattle.

[29] Fox D.G., Tedeschi L.O., Tylutki T.P., Russell J.B., Van Amburgh M.E., Chase L.E., Pell A.N., Overton T.R. (2004). The Cornell Net Carbohydrate and Protein System model for evaluating herd nutrition and nutrient excretion. Anim. Feed Sci. Technol..

[30] Anderson H.R., Spix C., Medina S., Schouten J.P., Castellsague J., Rossi G., Zmirou D., Touloumi G., Wojtyniak B., Ponka A. (1997). Air pollution and daily admissions for chronic obstructive pulmonary disease in 6 European cities: Results from the APHEA project. Eur. Respir. J..

[31] Regole di Categoria di Prodotto (RCP) Formaggio Grana Padano DOP (NACE 10.51.40) Valentina Castellani, Carlo Proserpio, Pieter Ravaglia, Luca Gianelli, Lucrezia Lamastra, Federico Froldi, Maurizio Moschini, Annamaria Boldini, Angelo Stroppa (Schema nazionale volontario “Made Green in Italy”, 2021). https://www.mase.gov.it/sites/default/files/archivio/allegati/impronta_ambientale/rcp_grana_padano_v_1_0.pdf.

[32] Derwent R.G., Jenkin M.E., Saunders S.M., Pilling M.J. (1998). Photochemical ozone creation potentials for organic compounds in northwest Europe calculated with a master chemical mechanism. Atmos. Environ..

[33] Presumido P.H., Sousa F., Gonçalves A., Bosco T.C.D., Feliciano M. (2018). Environmental Impacts of the Beef Production Chain in the Northeast of Portugal Using Life Cycle Assessment. Agriculture.

[34] Phan N.T., Kim K.H., Parker D., Jeon E.C., Sa J.H., Cho C.S. (2012). Effect of beef cattle manure application rate on CH4 and CO2 emissions. Atmos. Environ..

[35] Castanheira É.G., Dias A.C., Arroja L., Amaro R. (2010). The environmental performance of milk production on a typical Portuguese dairy farm. Agric. Syst..

[36] Froldi F., Lamastra L., Trevisan M., Mambretti D., Moschini M. (2022). Environmental Impacts of Cow’s Milk in Northern Italy: Effects of Farming Performance. J. Clean. Prod..

[37] The European Dairy Association (EDA) (2016). Product Environmental Footprint Category Rules for Dairy Products. https://ec.europa.eu/environment/eussd/smgp/pdf/PEFCR-DairyProducts_2018-04-225_V1.pdf.

[38] (2015). A common carbon footprint approach for dairy. The IDF Guide to Standard Life Cycle Assessment Methodology of Dairy Sector.

[39] IPCC (2019). Chapter 10. Emissions from Livestock and Manure Management. 2019 IPCC Guidelines for National Greenhouse Gas Inventories.

[40] (2016). D.g.r. 16 maggio 2016—N. X/5171. Approvazione del Programma D’azione Regionale per la Protezione Delle Acque Dall’inquinamento Provocato dai Nitrati Provenienti da Fonti Agricole Nelle Zone Vulnerabili ai Sensi Della Direttiva Nitrati 91/676/CEE. D.g.r. Regione Lombardia, Italy. https://www.gse.it/normativa_site/GSE%20Documenti%20normativa/LOMBARDIA_DGR_nX5171__16_05_2016.pdf.

[41] D.g.r (2016). 18 luglio 2016—N. X/5418 Linee Guida per la Protezione Delle Acque Dall’inquinamento Provocato dai Nitrati Provenienti da Fonti Agricole Nelle Zone Non Vulnerabili ai Sensi Della Direttiva Nitrati 91/676/CEE. D.g.r. Regione Lombardia, Italy. http://fodaflombardia.conaf.it/sites/fodaflombardia.conaf.it/files/d.g.r._5418_2016.pdf.

[42] IPCC (2006). Chapter 10. Emissions from Livestock and Manure Management. 2006 IPCC Guidelines for National Greenhouse Gas Inventories.

[43] EEA (2013). EMEP/EEA Air Pollutant Emission Inventory Guidebook 2013—Update July 2015.

[44] EEA (2016). EMEP/EEA Air Pollutant Emission Inventory Guidebook 2016.

[45] IPCC (2006). Chapter 11. N_2_O Emissions from managed soils, and CO_2_ emissions from lime and urea application. 2006 IPCC Guidelines for National Greenhouse Gas Inventories.

[46] Stocker T.F., Qin D., Plattner G.-K., Tignor M.M.B., Allen S.K., Boschung J., Nauels A., Xia Y., Bex V., Midgley P.M. (2013). Climate Change 2013 the Physical Science Basis Working Group I Contribution to the Fifth Assessment Report of the Intergovernmental Panel on Climate Change.

[47] Van Zelm R., Huijbregts M.A.J., Den Hollander H.A., Van Jaarsveld H.A., Sauter F.J., Struijs J., Van Wijnen H.J., Van de Meent D. (2008). European characterization factors for human health damage of PM10 and ozone in life cycle impact assessment. Atmos. Environ..

[48] (2019). PRé Consultants bv. SimaPro 9.0.0.35, LCA Software. Amersfoort. The Netherlands. https://simapro.com/products/environmental-footprint-database/.

[49] AOAC International (2000). Official Methods of Analysis.

[50] Neuhäuser M., Bretz F. (2001). Nonparametric all-pairs multiple comparisons. Biom. J. J. Math. Methods Biosci..

[51] (2019). SAS Institute. JMP®.

[52] Battini F., Agostini A., Tabaglio V., Amaducci S. (2016). Environmental impacts of different dairy farming systems in the Po Valley. J. Clean. Prod..

[53] Baldini C., Bava L., Zucali M., Guarino M. (2018). Milk production life cycle assessment: A comparison between estimated and measured emission inventory for manure handling. Sci. Total Environ..

[54] Bava L., Bacenetti J., Gislon G., Pellegrino L., D’Incecco P., Sandrucci A., Tamburini A., Fiala M., Zucali M. (2018). Impact assessment of traditional food manufacturing: The case of Grana Padano cheese. Sci. Total Environ..

[55] Lovarelli D., Bava L., Zucali M., D’Imporzano G., Adani F., Tamburini A., Sandrucci A. (2019). Improvements to dairy farms for environmental sustainability in Grana Padano and Parmigiano Reggiano production systems. Ital. J. Anim. Sci..

[56] Famiglietti J., Guerci M., Proserpio C., Ravaglia P., Motta M. (2019). Development and testing of the Product Environmental Footprint Milk Tool: A comprehensive LCA tool for dairy products. Sci. Total Environ..

[57] Lovarelli D., Leso L., Bonfanti M., Porto S.M.C., Barbari M., Guarino M. (2023). Climate change and socio-economic assessment of PLF in dairy farms: Three case studies. Sci. Total Environ..

[58] Dewhurst R.J., Evans R.T., Mottram T.T., Spanel P., Smith D. (2001). Assessment of rumen processes by selected-ion-flow-tube mass spectrometric analysis of rumen gases. J. Dairy Sci..

[59] Pandey B., Chen L. (2021). Technologies to recover nitrogen from livestock manure—A review. Sci. Total Environ..

[60] Thompson T.L., Cheng P.H., Li Y. (2009). The potential contribution of sub-surface drip irrigation to water saving agriculture in the western, USA. Agric. Sci. China.

[61] Galdeano-Gómez E., Aznar-Sánchez J.A., Pérez-Mesa J.C. (2013). Sustainability dimensions related to agricultural-based development: The experience of 50 years of intensive farming in almería (Spain). Int. J. Agric. Sustain..

[62] Wardal W.J. (2015). Factors determining the choice of a housing system for dairy cows. Probl. Inż. Roln..

[63] Ravishankara A.R., Kulenstierna J., Michalopoulou E., Höglund-Isaksson L., Zhang Y., Seltzer K., Ru M., Castelino R., Faluvegi G., Naik V. (2021). Global Methane Assessment: Benefits and Costs of Mitigating Methane Emissions. United Nations Environment Programme. https://www.unep.org/resources/report/global-methane-assessment-benefits-and-costs-mitigating-methane-emissions.

[64] Appuhamy J.A.D.R.N., Moraes L.E., Wagner-Riddle C., Casper D.P., Kebreab E. (2018). Predicting manure volatile solid output of lactating dairy cows. J. Dairy Sci..

[65] Rotz C.A. (2018). Modeling greenhouse gas emissions from dairy farms. J. Dairy Sci..

[66] Dourmad J.-Y., Rigolot C., van de Werf H., Rowlinson P., Steele M., Nefzaoui A. (2008). Emission of greenhouse gas, developing management and animal farming systems to assist mitigation. Livestock and Global Climate Change.

[67] Howard C.J., Kumar A., Mitloehner F., Stackhouse K., Green P.G., Flocchini R.G., Kleeman M.J. (2010). Direct measurements of the ozone formation potential from livestock and poultry waste emissions. Environ. Sci. Technol..

[68] Place S.E., Mitloehner F.M., Kebreab E. (2013). Air quality in sustainability: Greenhouse gases and volatile organic compounds. Sustainable Animal Agriculture.

[69] Putman B., Rotz C.A., Thoma G. (2023). A comprehensive environmental assessment of beef production and consumption in the United States. J. Clean. Prod..

[70] Chung M.Y., Beene M., Ashkan S., Krauter C., Hason A.S. (2010). Evaluation of non-enteric sources of non-methane volatile organic compounds (NMVOC) emissions from dairies. Atmos. Environ..

[71] Bonifacio H.F., Rotz C.A., Hafner S.D., Montes F., Cohen M., Mitloehner F.M. (2017). A process-based emission model of volatile organic compounds from silage sources on farms. Atmos. Environ..

[72] Berton M., Bovolenta S., Corazzin M., Gallo L., Pinterits S., Ramanzin M., Ressi W., Spigarelli C., Zuliani A., Sturaro E. (2021). Environmental impacts of milk production and processing in the Eastern Alps: A “cradle-to-dairy gate” LCA approach. J. Clean. Prod..

[73] Pirlo G., Lolli S. (2019). Environmental impact of milk production from samples of organic and conventional farms in Lombardy (Italy). J. Clean. Prod..

[74] Gislon G., Ferrero F., Bava L., Borreani G., Dal Prà A., Pacchioli M.T., Sandrucci A., Zucali M., Tabacco E. (2020). Forage systems and sustainability of milk production: Feed efficiency, environmental impacts and soil carbon stocks. J. Clean. Prod..

[75] Biagetti E., Gislon G., Martella A., Zucali M., Bava L., Franco S., Sandrucci A. (2023). Comparison of the use of life cycle assessment and ecological footprint methods for evaluating environmental performances in dairy production. Sci. Total Environ..

