# Supplementary material for: Climate Change and Photochemical Ozone Creation Potential Impact Indicators of Cow Milk: A Comparison of Different Scenarios for a Diet Assessment
Source: Animals (Basel). 2024 Jun 7;14(12):1725. doi: 10.3390/ani14121725 (PMC11201073; doi:10.3390/ani14121725)
Supplement: Supplementary file 1 [file animals-14-01725-s001.zip › animals-3004812-supplementary/How to read the supplementary file.pdf]

How to read the supplementary files

Supplementary files report the statistical analysis output yielding Tables 3, 4, and 5.

The files are organized into three folders upon unzipping, providing outputs for Distribution and ANOVA analyses for the considered variables.

The Distribution file (folder Table 3) includes descriptive statistics and the Goodness-of-Fit Test for the Fitted Normal Distribution.

The ANOVA file (folder Table 3) includes the output for the model applied and the test used for means comparisons.

The files for paired analysis, including the test of means, are in folders Table 4 and Table 5 and are from the Distribution analysis.
