# Supplementary material for: Climate Change and Photochemical Ozone Creation Potential Impact Indicators of Cow Milk: A Comparison of Different Scenarios for a Diet Assessment
Source: Animals (Basel). 2024 Jun 7;14(12):1725. doi: 10.3390/ani14121725 (PMC11201073; doi:10.3390/ani14121725)

Distributions Herd=high-performing, Indicator=CC kgCO2eq

Total_Difference_CNCPS_IPCC

Compare Distributions

Show	Distribution		AICc ^	BIC	-2*LogLikelihood
☑	Normal	—	-22.28513	-22.09792	-27.37604

Summary Statistics

Mean	-0.181276
Std Dev	0.0943315
Std Err Mean	0.0252112
Upper 95% Mean	-0.126811
Lower 95% Mean	-0.235742
N	14
N Missing	0

Fitted Normal Distribution

Parameter		Estimate	Std Error	Lower 95%	Upper 95%
Location	μ	-0.181276	0.0252112	-0.235742	-0.126811
Dispersion	σ	0.0943315	0.0188663	0.068386	0.1519721

Measures	
-2*LogLikelihood	-27.37604
AICc	-22.28513
BIC	-22.09792

Goodness-of-Fit Test

	W	Prob<W
Shapiro-Wilk	0.9119838	0.1684

	A²	Simulated p-Value
Anderson-Darling	0.4602409	0.2244

Note: Ho = The data is from the Normal distribution. Small p-values reject Ho.

Test Mean

Hypothesized Value	0
Actual Estimate	-0.1813
DF	13
Std Dev	0.09433

	t Test	Signed-Rank
Test Statistic	-7.1903	-52.5000
Prob > |t|	<.0001*	0.0001*
Prob > t	1.0000	0.9999
Prob < t	<.0001*	<.0001*

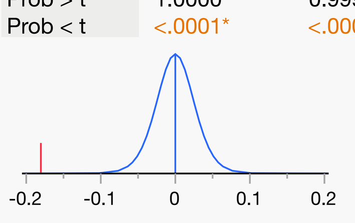

Distributions Herd=high-performing, Indicator=CC-biogenic kgCO2eq

Total_Difference_CNCPS_IPCC

Compare Distributions

Show	Distribution		AICc ^	BIC	-2*LogLikelihood
☑	Normal	—	-22.28513	-22.09792	-27.37604

Summary Statistics

Mean	-0.181276
Std Dev	0.0943315
Std Err Mean	0.0252112
Upper 95% Mean	-0.126811
Lower 95% Mean	-0.235742
N	14
N Missing	0

Fitted Normal Distribution

Parameter		Estimate	Std Error	Lower 95%	Upper 95%
Location	μ	-0.181276	0.0252112	-0.235742	-0.126811
Dispersion	σ	0.0943315	0.0188663	0.068386	0.1519721

Measures	
-2*LogLikelihood	-27.37604
AICc	-22.28513
BIC	-22.09792

Goodness-of-Fit Test

	W	Prob<W
Shapiro-Wilk	0.9119838	0.1684

	A²	Simulated p-Value
Anderson-Darling	0.4602411	0.2188

Note: Ho = The data is from the Normal distribution. Small p-values reject Ho.

Test Mean

Hypothesized Value	0
Actual Estimate	-0.1813
DF	13
Std Dev	0.09433

	t Test	Signed-Rank
Test Statistic	-7.1903	-52.5000
Prob > |t|	<.0001*	0.0001*
Prob > t	1.0000	0.9999
Prob < t	<.0001*	<.0001*

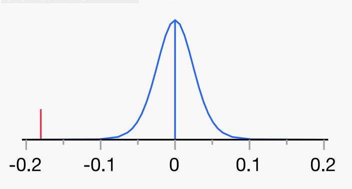

Distributions Herd=high-performing, Indicator=CC-fossil kgCO2eq

Total_Difference_CNCPS_IPCC

Compare Distributions

Show	Distribution		AICc ^	BIC	-2*LogLikelihood
☑	Normal	—	-483.9184	-483.7312	-489.0093

Summary Statistics

Mean	-5.083e-9
Std Dev	6.5236e-9
Std Err Mean	1.7435e-9
Upper 95% Mean	-1.316e-9
Lower 95% Mean	-8.849e-9
N	14
N Missing	0

Fitted Normal Distribution

Parameter		Estimate	Std Error	Lower 95%	Upper 95%
Location	μ	-5.083e-9	1.7435e-9	-8.849e-9	-1.316e-9
Dispersion	σ	6.5236e-9	1.3047e-9	4.7293e-9	1.051e-8

Measures	
-2*LogLikelihood	-489.0093
AICc	-483.9184
BIC	-483.7312

Goodness-of-Fit Test

	W	Prob<W
Shapiro-Wilk	0.7684642	0.0021*

	A²	Simulated p-Value
Anderson-Darling	1.3385186	0.0004*

Note: Ho = The data is from the Normal distribution. Small p-values reject Ho.

Test Mean

Hypothesized Value	0
Actual Estimate	-5.1e-9
DF	13
Std Dev	6.52e-9

	t Test	Signed-Rank
Test Statistic	-2.9153	-52.5000
Prob > |t|	0.0120*	0.0001*
Prob > t	0.9940	0.9999
Prob < t	0.0060*	<.0001*

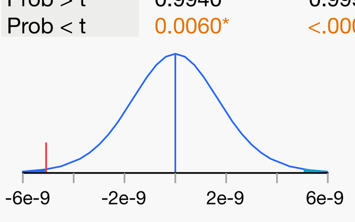

Distributions Herd=high-performing, Indicator=CC-LTU kgCO2eq

Total_Difference_CNCPS_IPCC

Summary Statistics

Mean	0
Std Dev	0
Std Err Mean	0
Upper 95% Mean	0
Lower 95% Mean	0
N	14
N Missing	0

Test Mean

Hypothesized Value	0
Actual Estimate	0
DF	13
Std Dev	0

	t Test	Signed-Rank
Test Statistic	.	0.0000
Prob > |t|	.	1.0000
Prob > t	.	0.5000
Prob < t	.	0.5000

Distributions Herd=high-performing, Indicator=POCP kgNMVOCeq

Total_Difference_CNCPS_IPCC

Compare Distributions

Show	Distribution		AICc ^	BIC	-2*LogLikelihood
☑	Normal	—	-249.6894	-249.5022	-254.7803

Summary Statistics

Mean	-5.385e-5
Std Dev	2.8022e-5
Std Err Mean	7.4892e-6
Upper 95% Mean	-3.767e-5
Lower 95% Mean	-0.00007
N	14
N Missing	0

Fitted Normal Distribution

Parameter		Estimate	Std Error	Lower 95%	Upper 95%
Location	μ	-5.385e-5	7.4892e-6	-0.00007	-3.767e-5
Dispersion	σ	2.8022e-5	5.6044e-6	2.0315e-5	4.5145e-5

Measures	
-2*LogLikelihood	-254.7803
AICc	-249.6894
BIC	-249.5022

Goodness-of-Fit Test

	W	Prob<W
Shapiro-Wilk	0.9119838	0.1684

	A²	Simulated p-Value
Anderson-Darling	0.4602408	0.2064

Note: Ho = The data is from the Normal distribution. Small p-values reject Ho.

Test Mean

Hypothesized Value	0
Actual Estimate	-0.0001
DF	13
Std Dev	2.8e-5

	t Test	Signed-Rank
Test Statistic	-7.1903	-52.5000
Prob > |t|	<.0001*	0.0001*
Prob > t	1.0000	0.9999
Prob < t	<.0001*	<.0001*

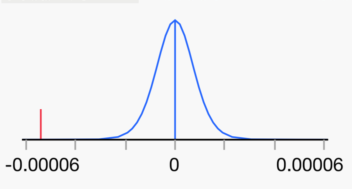

Distributions Herd=low-performing, Indicator=CC kgCO2eq

Total_Difference_CNCPS_IPCC

Compare Distributions

Show	Distribution		AICc ^	BIC	-2*LogLikelihood
☑	Normal	—	-7.894355	-7.707149	-12.98526

Summary Statistics

Mean	-0.023948
Std Dev	0.1577122
Std Err Mean	0.0421503
Upper 95% Mean	0.0671122
Lower 95% Mean	-0.115008
N	14
N Missing	0

Fitted Normal Distribution

Parameter		Estimate	Std Error	Lower 95%	Upper 95%
Location	μ	-0.023948	0.0421503	-0.115008	0.0671122
Dispersion	σ	0.1577122	0.0315424	0.1143341	0.254081

Measures	
-2*LogLikelihood	-12.98526
AICc	-7.894355
BIC	-7.707149

Goodness-of-Fit Test

	W	Prob<W
Shapiro-Wilk	0.9497899	0.5574

	A²	Simulated p-Value
Anderson-Darling	0.3552428	0.4108

Note: Ho = The data is from the Normal distribution. Small p-values reject Ho.

Test Mean

Hypothesized Value	0
Actual Estimate	-0.0239
DF	13
Std Dev	0.15771

	t Test	Signed-Rank
Test Statistic	-0.5682	-13.5000
Prob > |t|	0.5796	0.4263
Prob > t	0.7102	0.7869
Prob < t	0.2898	0.2131

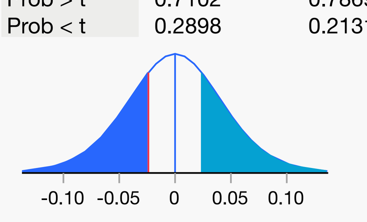

Distributions Herd=low-performing, Indicator=CC-biogenic kgCO2eq

Total_Difference_CNCPS_IPCC

Compare Distributions

Show	Distribution		AICc ^	BIC	-2*LogLikelihood
☑	Normal	—	-7.894356	-7.70715	-12.98526

Summary Statistics

Mean	-0.023948
Std Dev	0.1577122
Std Err Mean	0.0421503
Upper 95% Mean	0.0671122
Lower 95% Mean	-0.115008
N	14
N Missing	0

Fitted Normal Distribution

Parameter		Estimate	Std Error	Lower 95%	Upper 95%
Location	μ	-0.023948	0.0421503	-0.115008	0.0671122
Dispersion	σ	0.1577122	0.0315424	0.114334	0.254081

Measures	
-2*LogLikelihood	-12.98526
AICc	-7.894356
BIC	-7.70715

Goodness-of-Fit Test

	W	Prob<W
Shapiro-Wilk	0.94979	0.5574

	A²	Simulated p-Value
Anderson-Darling	0.3552426	0.4264

Note: Ho = The data is from the Normal distribution. Small p-values reject Ho.

Test Mean

Hypothesized Value	0
Actual Estimate	-0.0239
DF	13
Std Dev	0.15771

	t Test	Signed-Rank
Test Statistic	-0.5682	-13.5000
Prob > |t|	0.5796	0.4263
Prob > t	0.7102	0.7869
Prob < t	0.2898	0.2131

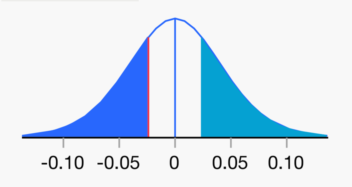

Distributions Herd=low-performing, Indicator=CC-fossil kgCO2eq

Total_Difference_CNCPS_IPCC

Compare Distributions

Show	Distribution		AICc ^	BIC	-2*LogLikelihood
☑	Normal	—	-458.9892	-458.802	-464.0801

Summary Statistics

Mean	-1.349e-8
Std Dev	1.5891e-8
Std Err Mean	4.247e-9
Upper 95% Mean	-4.318e-9
Lower 95% Mean	-2.267e-8
N	14
N Missing	0

Fitted Normal Distribution

Parameter		Estimate	Std Error	Lower 95%	Upper 95%
Location	μ	-1.349e-8	4.247e-9	-2.267e-8	-4.318e-9
Dispersion	σ	1.5891e-8	3.1782e-9	1.152e-8	2.5601e-8

Measures	
-2*LogLikelihood	-464.0801
AICc	-458.9892
BIC	-458.802

Goodness-of-Fit Test

	W	Prob<W
Shapiro-Wilk	0.7477248	0.0012*

	A²	Simulated p-Value
Anderson-Darling	1.1415193	0.0048*

Note: Ho = The data is from the Normal distribution. Small p-values reject Ho.

Test Mean

Hypothesized Value	0
Actual Estimate	-1.3e-8
DF	13
Std Dev	1.59e-8

	t Test	Signed-Rank
Test Statistic	-3.1770	-52.5000
Prob > |t|	0.0073*	0.0001*
Prob > t	0.9964	0.9999
Prob < t	0.0036*	<.0001*

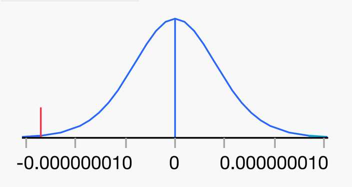

Distributions Herd=low-performing, Indicator=CC-LTU kgCO2eq

Total_Difference_CNCPS_IPCC

Summary Statistics

Mean	0
Std Dev	0
Std Err Mean	0
Upper 95% Mean	0
Lower 95% Mean	0
N	14
N Missing	0

Test Mean

Hypothesized Value	0
Actual Estimate	0
DF	13
Std Dev	0

	t Test	Signed-Rank
Test Statistic	.	0.0000
Prob > |t|	.	1.0000
Prob > t	.	0.5000
Prob < t	.	0.5000

Distributions Herd=low-performing, Indicator=POCP kgNMVOCeq

Total_Difference_CNCPS_IPCC

Compare Distributions

Show	Distribution		AICc ^	BIC	-2*LogLikelihood
☑	Normal	—	-235.2986	-235.1114	-240.3895

Summary Statistics

Mean	-7.114e-6
Std Dev	4.685e-5
Std Err Mean	1.2521e-5
Upper 95% Mean	1.9936e-5
Lower 95% Mean	-3.416e-5
N	14
N Missing	0

Fitted Normal Distribution

Parameter		Estimate	Std Error	Lower 95%	Upper 95%
Location	μ	-7.114e-6	1.2521e-5	-3.416e-5	1.9936e-5
Dispersion	σ	4.685e-5	9.37e-6	3.3964e-5	7.5477e-5

Measures	
-2*LogLikelihood	-240.3895
AICc	-235.2986
BIC	-235.1114

Goodness-of-Fit Test

	W	Prob<W
Shapiro-Wilk	0.94979	0.5574

	A²	Simulated p-Value
Anderson-Darling	0.3552427	0.4180

Note: Ho = The data is from the Normal distribution. Small p-values reject Ho.

Test Mean

Hypothesized Value	0
Actual Estimate	-7.1e-6
DF	13
Std Dev	4.68e-5

	t Test	Signed-Rank
Test Statistic	-0.5682	-13.5000
Prob > |t|	0.5796	0.4263
Prob > t	0.7102	0.7869
Prob < t	0.2898	0.2131

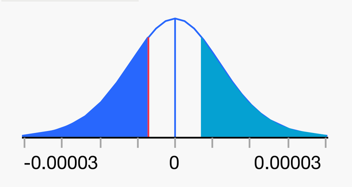

Distributions Herd=mid-performing, Indicator=CC kgCO2eq

Total_Difference_CNCPS_IPCC

Compare Distributions

Show	Distribution		AICc ^	BIC	-2*LogLikelihood
☑	Normal	—	-45.37015	-43.27848	-49.87015

Summary Statistics

Mean	-0.119319
Std Dev	0.097887
Std Err Mean	0.0188384
Upper 95% Mean	-0.080596
Lower 95% Mean	-0.158042
N	27
N Missing	0

Fitted Normal Distribution

Parameter		Estimate	Std Error	Lower 95%	Upper 95%
Location	μ	-0.119319	0.0188384	-0.158042	-0.080596
Dispersion	σ	0.097887	0.0137069	0.0770876	0.1341475

Measures	
-2*LogLikelihood	-49.87015
AICc	-45.37015
BIC	-43.27848

Goodness-of-Fit Test

	W	Prob<W
Shapiro-Wilk	0.9647619	0.4711

	A²	Simulated p-Value
Anderson-Darling	0.4332023	0.2888

Note: Ho = The data is from the Normal distribution. Small p-values reject Ho.

Test Mean

Hypothesized Value	0
Actual Estimate	-0.1193
DF	26
Std Dev	0.09789

	t Test	Signed-Rank
Test Statistic	-6.3338	-171.000
Prob > |t|	<.0001*	<.0001*
Prob > t	1.0000	1.0000
Prob < t	<.0001*	<.0001*

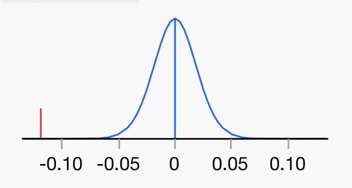

Distributions Herd=mid-performing, Indicator=CC-biogenic kgCO2eq

Total_Difference_CNCPS_IPCC

Compare Distributions

Show	Distribution		AICc ^	BIC	-2*LogLikelihood
☑	Normal	—	-45.37015	-43.27848	-49.87015

Summary Statistics

Mean	-0.119319
Std Dev	0.097887
Std Err Mean	0.0188384
Upper 95% Mean	-0.080596
Lower 95% Mean	-0.158042
N	27
N Missing	0

Fitted Normal Distribution

Parameter		Estimate	Std Error	Lower 95%	Upper 95%
Location	μ	-0.119319	0.0188384	-0.158042	-0.080596
Dispersion	σ	0.097887	0.0137069	0.0770876	0.1341475

Measures	
-2*LogLikelihood	-49.87015
AICc	-45.37015
BIC	-43.27848

Goodness-of-Fit Test

	W	Prob<W
Shapiro-Wilk	0.9647619	0.4711

	A²	Simulated p-Value
Anderson-Darling	0.4332024	0.2860

Note: Ho = The data is from the Normal distribution. Small p-values reject Ho.

Test Mean

Hypothesized Value	0
Actual Estimate	-0.1193
DF	26
Std Dev	0.09789

	t Test	Signed-Rank
Test Statistic	-6.3338	-171.000
Prob > |t|	<.0001*	<.0001*
Prob > t	1.0000	1.0000
Prob < t	<.0001*	<.0001*

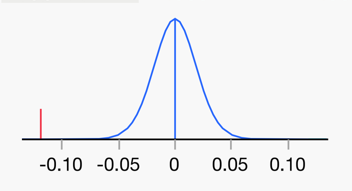

Distributions Herd=mid-performing, Indicator=CC-fossil kgCO2eq

Total_Difference_CNCPS_IPCC

Compare Distributions

Show	Distribution		AICc ^	BIC	-2*LogLikelihood
☑	Normal	—	-936.5848	-934.4931	-941.0848

Summary Statistics

Mean	-7.922e-9
Std Dev	6.6549e-9
Std Err Mean	1.2807e-9
Upper 95% Mean	-5.29e-9
Lower 95% Mean	-1.055e-8
N	27
N Missing	0

Fitted Normal Distribution

Parameter		Estimate	Std Error	Lower 95%	Upper 95%
Location	μ	-7.922e-9	1.2807e-9	-1.055e-8	-5.29e-9
Dispersion	σ	6.6549e-9	9.319e-10	5.2408e-9	9.1201e-9

Measures	
-2*LogLikelihood	-941.0848
AICc	-936.5848
BIC	-934.4931

Goodness-of-Fit Test

	W	Prob<W
Shapiro-Wilk	0.8169248	0.0003*

	A²	Simulated p-Value
Anderson-Darling	2.1840441	<.0001*

Note: Ho = The data is from the Normal distribution. Small p-values reject Ho.

Test Mean

Hypothesized Value	0
Actual Estimate	-7.9e-9
DF	26
Std Dev	6.65e-9

	t Test	Signed-Rank
Test Statistic	-6.1857	-189.000
Prob > |t|	<.0001*	<.0001*
Prob > t	1.0000	1.0000
Prob < t	<.0001*	<.0001*

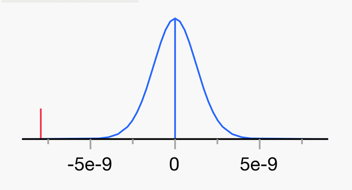

Distributions Herd=mid-performing, Indicator=CC-LTU kgCO2eq

Total_Difference_CNCPS_IPCC

Summary Statistics

Mean	0
Std Dev	0
Std Err Mean	0
Upper 95% Mean	0
Lower 95% Mean	0
N	27
N Missing	0

Test Mean

Hypothesized Value	0
Actual Estimate	0
DF	26
Std Dev	0

	t Test	Signed-Rank
Test Statistic	.	0.0000
Prob > |t|	.	1.0000
Prob > t	.	0.5000
Prob < t	.	0.5000

Distributions Herd=mid-performing, Indicator=POCP kgNMVOCeq

Total_Difference_CNCPS_IPCC

Compare Distributions

Show	Distribution		AICc ^	BIC	-2*LogLikelihood
☑	Normal	—	-483.9355	-481.8438	-488.4355

Summary Statistics

Mean	-3.544e-5
Std Dev	2.9078e-5
Std Err Mean	5.5961e-6
Upper 95% Mean	-0.000024
Lower 95% Mean	-0.000047
N	27
N Missing	0

Fitted Normal Distribution

Parameter		Estimate	Std Error	Lower 95%	Upper 95%
Location	μ	-3.544e-5	5.5961e-6	-0.000047	-0.000024
Dispersion	σ	2.9078e-5	4.0718e-6	0.0000229	3.985e-5

Measures	
-2*LogLikelihood	-488.4355
AICc	-483.9355
BIC	-481.8438

Goodness-of-Fit Test

	W	Prob<W
Shapiro-Wilk	0.9647619	0.4711

	A²	Simulated p-Value
Anderson-Darling	0.4332024	0.2952

Note: Ho = The data is from the Normal distribution. Small p-values reject Ho.

Test Mean

Hypothesized Value	0
Actual Estimate	-3.5e-5
DF	26
Std Dev	0.00003

	t Test	Signed-Rank
Test Statistic	-6.3338	-171.000
Prob > |t|	<.0001*	<.0001*
Prob > t	1.0000	1.0000
Prob < t	<.0001*	<.0001*

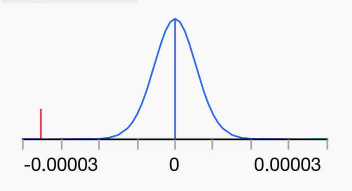

Supplement: Supplementary file 1 [file animals-14-01725-s001.zip › animals-3004812-supplementary/Tabel 5/Distribution and Test of Total_Difference_CNCPS_IPCC.pdf]
