# Supplementary material for: Climate Change and Photochemical Ozone Creation Potential Impact Indicators of Cow Milk: A Comparison of Different Scenarios for a Diet Assessment
Source: Animals (Basel). 2024 Jun 7;14(12):1725. doi: 10.3390/ani14121725 (PMC11201073; doi:10.3390/ani14121725)
Supplement: Supplementary file 1 [file animals-14-01725-s001.zip › animals-3004812-supplementary/Table 3/Anova of Barn management.pdf]

## Oneway Analysis of Barn management By Herd Indicator=CC kgCO2eq

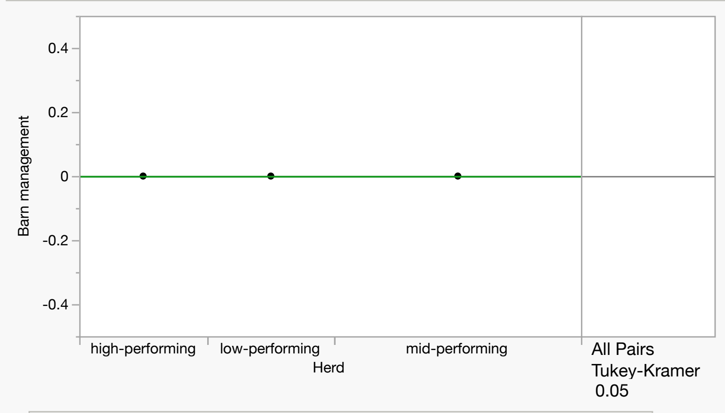


### Oneway Anova

#### Summary of Fit

| | |
|---|---|
| Rsquare | . |
| Adj Rsquare | . |
| Root Mean Square Error | 0 |
| Mean of Response | 0 |
| Observations (or Sum Wgts) | 55 |

#### Analysis of Variance

| Source | DF | Sum of Squares | Mean Square | F Ratio | Prob > F |
|---|---|---|---|---|---|
| Herd | 2 | 0 | 0 | . | . |
| Error | 52 | 0 | 0 | | |
| C. Total | 54 | 0 | | | |

#### Means for Oneway Anova

| Level | Number | Mean | Std Error | Lower 95% | Upper 95% |
|---|---|---|---|---|---|
| high-performing | 14 | 0 | 0 | 0 | 0 |
| low-performing | 14 | 0 | 0 | 0 | 0 |
| mid-performing | 27 | 0 | 0 | 0 | 0 |

Std Error uses a pooled estimate of error variance

### Means Comparisons

#### Comparisons for all pairs using Tukey-Kramer HSD

##### Confidence Quantile

| q* | Alpha |
|---|---|
| 2.41260 | 0.05 |

##### HSD Threshold Matrix

Abs(Dif)-HSD

| | high-performing | low-performing | mid-performing |
|---|---|---|---|
| high-performing | 0 | 0 | 0 |
| low-performing | 0 | 0 | 0 |
| mid-performing | 0 | 0 | 0 |

Positive values show pairs of means that are significantly different.

##### Connecting Letters Report

| Level | Mean |
|---|---|
| high-performing | 0 |
| low-performing | 0 |
| mid-performing | 0 |

Levels not connected by same letter are significantly different.

##### Ordered Differences Report

| Level | - Level | Difference | Std Err Dif | Lower CL | Upper CL | p-Value |
|---|---|---|---|---|---|---|
| low-performing | high-performing | 0 | 0 | 0 | 0 | . |
| mid-performing | high-performing | 0 | 0 | 0 | 0 | . |
| mid-performing | low-performing | 0 | 0 | 0 | 0 | . |

Excluded Rows 3

## Oneway Analysis of Barn management By Herd Indicator=CC-biogenic kgCO2eq

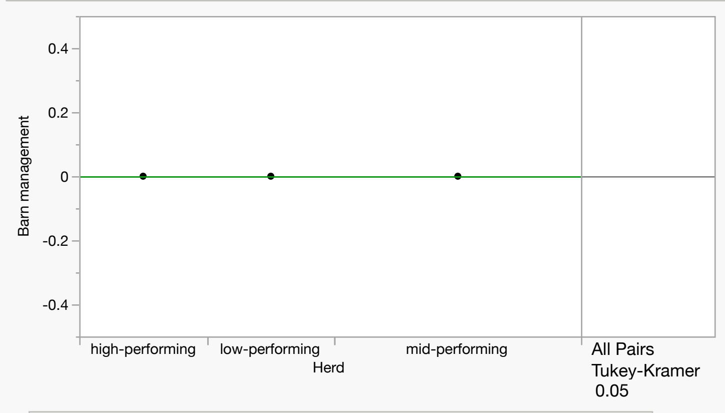


### Oneway Anova

#### Summary of Fit

| | |
|---|---|
| Rsquare | . |
| Adj Rsquare | . |
| Root Mean Square Error | 0 |
| Mean of Response | 0 |
| Observations (or Sum Wgts) | 55 |

#### Analysis of Variance

| Source | DF | Sum of Squares | Mean Square | F Ratio | Prob > F |
|---|---|---|---|---|---|
| Herd | 2 | 0 | 0 | . | . |
| Error | 52 | 0 | 0 | | |
| C. Total | 54 | 0 | | | |

#### Means for Oneway Anova

| Level | Number | Mean | Std Error | Lower 95% | Upper 95% |
|---|---|---|---|---|---|
| high-performing | 14 | 0 | 0 | 0 | 0 |
| low-performing | 14 | 0 | 0 | 0 | 0 |
| mid-performing | 27 | 0 | 0 | 0 | 0 |

Std Error uses a pooled estimate of error variance

### Means Comparisons

#### Comparisons for all pairs using Tukey-Kramer HSD

##### Confidence Quantile

| q* | Alpha |
|---|---|
| 2.41260 | 0.05 |

##### HSD Threshold Matrix

Abs(Dif)-HSD

| | high-performing | low-performing | mid-performing |
|---|---|---|---|
| high-performing | 0 | 0 | 0 |
| low-performing | 0 | 0 | 0 |
| mid-performing | 0 | 0 | 0 |

Positive values show pairs of means that are significantly different.

##### Connecting Letters Report

| Level | Mean |
|---|---|
| high-performing | 0 |
| low-performing | 0 |
| mid-performing | 0 |

Levels not connected by same letter are significantly different.

##### Ordered Differences Report

| Level | - Level | Difference | Std Err Dif | Lower CL | Upper CL | p-Value |
|---|---|---|---|---|---|---|
| low-performing | high-performing | 0 | 0 | 0 | 0 | . |
| mid-performing | high-performing | 0 | 0 | 0 | 0 | . |
| mid-performing | low-performing | 0 | 0 | 0 | 0 | . |

Excluded Rows 3

## Oneway Analysis of Barn management By Herd Indicator=CC-fossil kgCO2eq

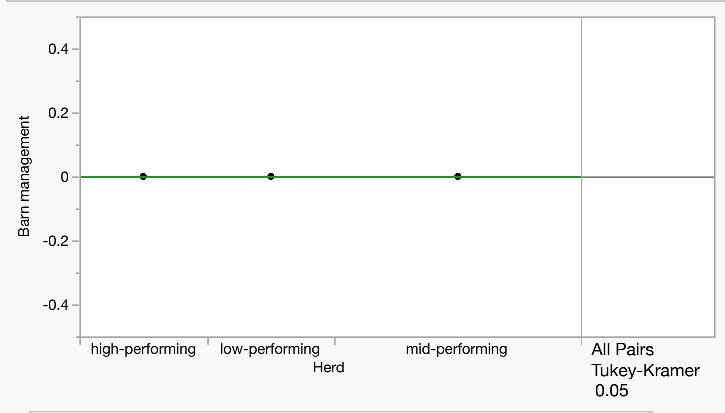


### Oneway Anova

#### Summary of Fit

| | |
|---|---|
| Rsquare | . |
| Adj Rsquare | . |
| Root Mean Square Error | 0 |
| Mean of Response | 0 |
| Observations (or Sum Wgts) | 55 |

#### Analysis of Variance

| Source | DF | Sum of Squares | Mean Square | F Ratio | Prob > F |
|---|---|---|---|---|---|
| Herd | 2 | 0 | 0 | . | . |
| Error | 52 | 0 | 0 | | |
| C. Total | 54 | 0 | | | |

#### Means for Oneway Anova

| Level | Number | Mean | Std Error | Lower 95% | Upper 95% |
|---|---|---|---|---|---|
| high-performing | 14 | 0 | 0 | 0 | 0 |
| low-performing | 14 | 0 | 0 | 0 | 0 |
| mid-performing | 27 | 0 | 0 | 0 | 0 |

Std Error uses a pooled estimate of error variance

### Means Comparisons

#### Comparisons for all pairs using Tukey-Kramer HSD

##### Confidence Quantile

| q* | Alpha |
|---|---|
| 2.41260 | 0.05 |

##### HSD Threshold Matrix

Abs(Dif)-HSD

| | high-performing | low-performing | mid-performing |
|---|---|---|---|
| high-performing | 0 | 0 | 0 |
| low-performing | 0 | 0 | 0 |
| mid-performing | 0 | 0 | 0 |

Positive values show pairs of means that are significantly different.

##### Connecting Letters Report

| Level | Mean |
|---|---|
| high-performing | 0 |
| low-performing | 0 |
| mid-performing | 0 |

Levels not connected by same letter are significantly different.

##### Ordered Differences Report

| Level | - Level | Difference | Std Err Dif | Lower CL | Upper CL | p-Value |
|---|---|---|---|---|---|---|
| low-performing | high-performing | 0 | 0 | 0 | 0 | . |
| mid-performing | high-performing | 0 | 0 | 0 | 0 | . |
| mid-performing | low-performing | 0 | 0 | 0 | 0 | . |

Excluded Rows 3

## Oneway Analysis of Barn management By Herd Indicator=CC-LTU kgCO2eq

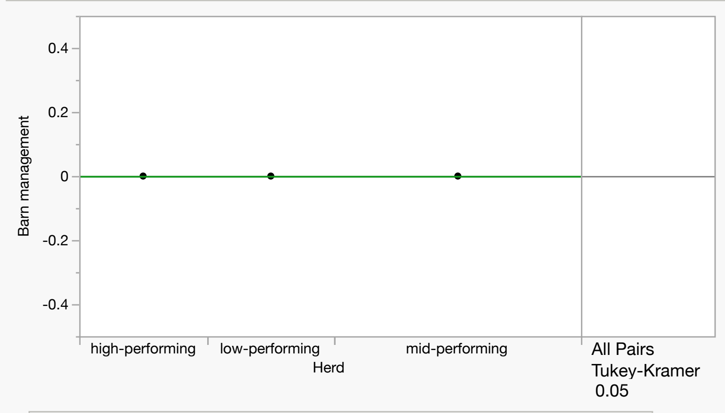


### Oneway Anova

#### Summary of Fit

| | |
|---|---|
| Rsquare | . |
| Adj Rsquare | . |
| Root Mean Square Error | 0 |
| Mean of Response | 0 |
| Observations (or Sum Wgts) | 55 |

#### Analysis of Variance

| Source | DF | Sum of Squares | Mean Square | F Ratio | Prob > F |
|---|---|---|---|---|---|
| Herd | 2 | 0 | 0 | . | . |
| Error | 52 | 0 | 0 | | |
| C. Total | 54 | 0 | | | |

#### Means for Oneway Anova

| Level | Number | Mean | Std Error | Lower 95% | Upper 95% |
|---|---|---|---|---|---|
| high-performing | 14 | 0 | 0 | 0 | 0 |
| low-performing | 14 | 0 | 0 | 0 | 0 |
| mid-performing | 27 | 0 | 0 | 0 | 0 |

Std Error uses a pooled estimate of error variance

### Means Comparisons

#### Comparisons for all pairs using Tukey-Kramer HSD

##### Confidence Quantile

| q* | Alpha |
|---|---|
| 2.41260 | 0.05 |

##### HSD Threshold Matrix

Abs(Dif)-HSD

| | high-performing | low-performing | mid-performing |
|---|---|---|---|
| high-performing | 0 | 0 | 0 |
| low-performing | 0 | 0 | 0 |
| mid-performing | 0 | 0 | 0 |

Positive values show pairs of means that are significantly different.

##### Connecting Letters Report

| Level | Mean |
|---|---|
| high-performing | 0 |
| low-performing | 0 |
| mid-performing | 0 |

Levels not connected by same letter are significantly different.

##### Ordered Differences Report

| Level | - Level | Difference | Std Err Dif | Lower CL | Upper CL | p-Value |
|---|---|---|---|---|---|---|
| low-performing | high-performing | 0 | 0 | 0 | 0 | . |
| mid-performing | high-performing | 0 | 0 | 0 | 0 | . |
| mid-performing | low-performing | 0 | 0 | 0 | 0 | . |

Excluded Rows 3

## Oneway Analysis of Barn management By Herd Indicator=POCP kgNMVOCeq

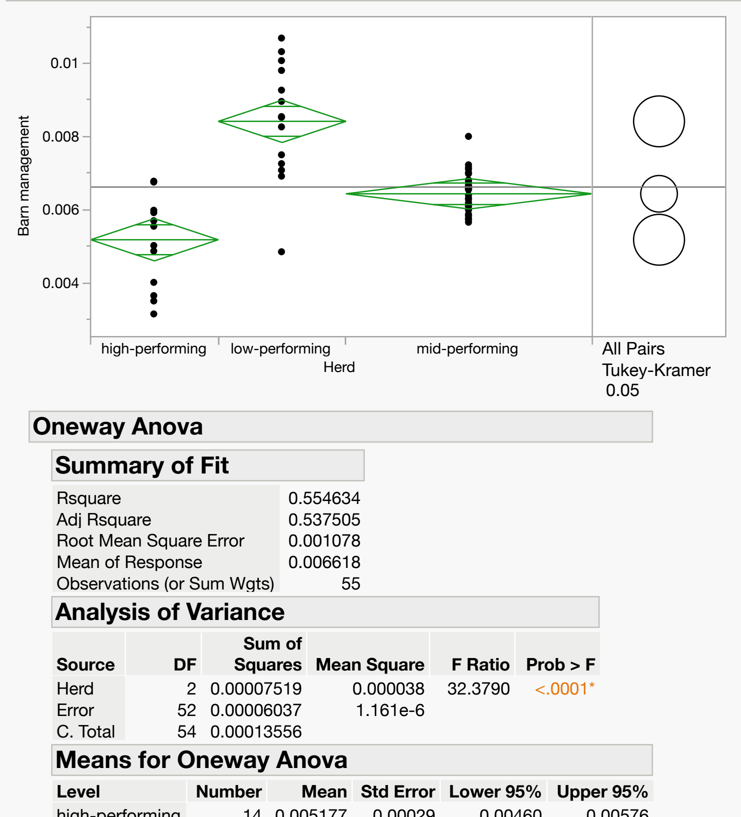


### Oneway Anova

#### Summary of Fit

| | |
|---|---|
| Rsquare | 0.554634 |
| Adj Rsquare | 0.537505 |
| Root Mean Square Error | 0.001078 |
| Mean of Response | 0.006618 |
| Observations (or Sum Wgts) | 55 |

#### Analysis of Variance

| Source | DF | Sum of Squares | Mean Square | F Ratio | Prob > F |
|---|---|---|---|---|---|
| Herd | 2 | 0.00007519 | 0.000038 | 32.3790 | <.0001* |
| Error | 52 | 0.00006037 | 1.161e-6 | | |
| C. Total | 54 | 0.00013556 | | | |

#### Means for Oneway Anova

| Level | Number | Mean | Std Error | Lower 95% | Upper 95% |
|---|---|---|---|---|---|
| high-performing | 14 | 0.005177 | 0.00029 | 0.00460 | 0.00576 |
| low-performing | 14 | 0.008415 | 0.00029 | 0.00784 | 0.00899 |
| mid-performing | 27 | 0.006434 | 0.00021 | 0.00602 | 0.00685 |

Std Error uses a pooled estimate of error variance

### Means Comparisons

#### Comparisons for all pairs using Tukey-Kramer HSD

##### Confidence Quantile

| q* | Alpha |
|---|---|
| 2.41260 | 0.05 |

##### HSD Threshold Matrix

Abs(Dif)-HSD

| | low-performing | mid-performing | high-performing |
|---|---|---|---|
| low-performing | -0.00098 | 0.00112 | 0.00226 |
| mid-performing | 0.00112 | -0.00071 | 0.00040 |
| high-performing | 0.00226 | 0.00040 | -0.00098 |

Positive values show pairs of means that are significantly different.

##### Connecting Letters Report

| Level | | | | Mean |
|---|---|---|---|---|
| low-performing | A | | | 0.00841500 |
| mid-performing | | B | | 0.00643398 |
| high-performing | | | C | 0.00517719 |

Levels not connected by same letter are significantly different.

##### Ordered Differences Report

| Level | - Level | Difference | Std Err Dif | Lower CL | Upper CL | p-Value |
|---|---|---|---|---|---|---|
| low-performing | high-performing | 0.0032378 | 0.0004073 | 0.0022552 | 0.0042204 | <.0001* |
| low-performing | mid-performing | 0.0019810 | 0.0003549 | 0.0011249 | 0.0028372 | <.0001* |
| mid-performing | high-performing | 0.0012568 | 0.0003549 | 0.0004006 | 0.0021129 | 0.0024* |

Excluded Rows 3
