# Supplementary material for: Climate Change and Photochemical Ozone Creation Potential Impact Indicators of Cow Milk: A Comparison of Different Scenarios for a Diet Assessment
Source: Animals (Basel). 2024 Jun 7;14(12):1725. doi: 10.3390/ani14121725 (PMC11201073; doi:10.3390/ani14121725)
Supplement: Supplementary file 1 [file animals-14-01725-s001.zip › animals-3004812-supplementary/Table 3/Anova of Bedding materials.pdf]

Oneway Analysis of Bedding materials By Herd Indicator=CC kgCO2eq

Oneway Anova

Summary of Fit

Rsquare	0.199692
Adj Rsquare	0.168911
Root Mean Square Error	0.016079
Mean of Response	0.01529
Observations (or Sum Wgts)	55

Analysis of Variance

Source	DF	Sum of Squares	Mean Square	F Ratio	Prob > F
Herd	2	0.00335440	0.001677	6.4875	0.0031*
Error	52	0.01344344	0.000259		
C. Total	54	0.01679784			

Means for Oneway Anova

Level	Number	Mean	Std Error	Lower 95%	Upper 95%
high-performing	14	0.008256	0.00430	-0.0004	0.01688
low-performing	14	0.028373	0.00430	0.0197	0.03700
mid-performing	27	0.012154	0.00309	0.0059	0.01836

Std Error uses a pooled estimate of error variance

Nonparametric Comparisons For All Pairs Using Steel-Dwass Method

q*	Alpha
2.34370	0.05

Level	- Level	Score Mean Difference	Std Err Dif	Z	p-Value	Hodges-Lehmann	Lower CL	Upper CL	Difference Plot
low-performing	high-performing	7.64286	3.109126	2.45820	0.0372*	0.010888	0.000433	0.0421345	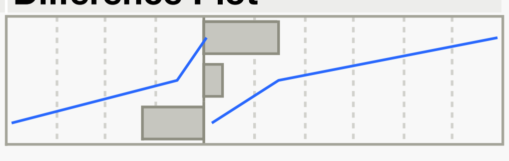
mid-performing	high-performing	4.33862	3.945055	1.09976	0.5142	0.002621	-0.003907	0.0109153	
mid-performing	low-performing	-8.83995	3.945227	-2.24067	0.0645	-0.008672	-0.027464	0.0010688	

Excluded Rows 3

Oneway Analysis of Bedding materials By Herd Indicator=CC-biogenic kgCO2eq

Oneway Anova

Summary of Fit

Rsquare	0.180837
Adj Rsquare	0.149331
Root Mean Square Error	4.34e-6
Mean of Response	3.435e-6
Observations (or Sum Wgts)	55

Analysis of Variance

Source	DF	Sum of Squares	Mean Square	F Ratio	Prob > F
Herd	2	2.1619e-10	1.081e-10	5.7397	0.0056*
Error	52	9.7928e-10	1.883e-11		
C. Total	54	1.19547e-9			

Means for Oneway Anova

Level	Number	Mean	Std Error	Lower 95%	Upper 95%
high-performing	14	1.6435e-6	1.1598e-6	-6.838e-7	3.9709e-6
low-performing	14	6.7546e-6	1.1598e-6	4.4273e-6	9.082e-6
mid-performing	27	2.6421e-6	8.3516e-7	9.6622e-7	4.318e-6

Std Error uses a pooled estimate of error variance

Nonparametric Comparisons For All Pairs Using Steel-Dwass Method

q*	Alpha
2.34370	0.05

Level	- Level	Score Mean Difference	Std Err Dif	Z	p-Value	Hodges-Lehmann	Lower CL	Upper CL	Difference Plot
low-performing	high-performing	7.78571	3.109126	2.50415	0.0329*	2.0198e-6	1.0112e-7	8.7349e-6	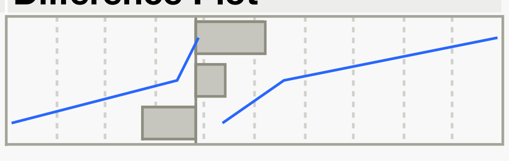
mid-performing	high-performing	5.74868	3.945055	1.45719	0.3117	8.3044e-7	-5.284e-7	2.523e-6	
mid-performing	low-performing	-7.10450	3.945227	-1.80078	0.1693	-1.511e-6	-5.341e-6	7.4359e-7	

Excluded Rows 3

Oneway Analysis of Bedding materials By Herd Indicator=CC-fossil kgCO2eq

Oneway Anova

Summary of Fit

Rsquare	0.198934
Adj Rsquare	0.168123
Root Mean Square Error	0.015405
Mean of Response	0.015026
Observations (or Sum Wgts)	55

Analysis of Variance

Source	DF	Sum of Squares	Mean Square	F Ratio	Prob > F
Herd	2	0.00306469	0.001532	6.4567	0.0031*
Error	52	0.01234090	0.000237		
C. Total	54	0.01540560			

Means for Oneway Anova

Level	Number	Mean	Std Error	Lower 95%	Upper 95%
high-performing	14	0.008153	0.00412	-0.0001	0.01641
low-performing	14	0.027494	0.00412	0.0192	0.03576
mid-performing	27	0.012125	0.00296	0.0062	0.01807

Std Error uses a pooled estimate of error variance

Nonparametric Comparisons For All Pairs Using Steel-Dwass Method

q*	Alpha
2.34370	0.05

Level	- Level	Score Mean Difference	Std Err Dif	Z	p-Value	Hodges-Lehmann	Lower CL	Upper CL	Difference Plot
low-performing	high-performing	7.78571	3.109126	2.50415	0.0329*	0.010883	0.000669	0.0397054	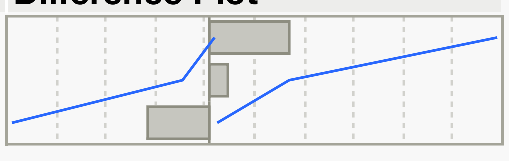
mid-performing	high-performing	4.44709	3.945055	1.12726	0.4972	0.002619	-0.003730	0.0109509	
mid-performing	low-performing	-8.83995	3.945227	-2.24067	0.0645	-0.008669	-0.027453	0.0010667	

Excluded Rows 3

Oneway Analysis of Bedding materials By Herd Indicator=CC-LTU kgCO2eq

Oneway Anova

Summary of Fit

Rsquare	0.099032
Adj Rsquare	0.064379
Root Mean Square Error	0.001113
Mean of Response	0.000261
Observations (or Sum Wgts)	55

Analysis of Variance

Source	DF	Sum of Squares	Mean Square	F Ratio	Prob > F
Herd	2	0.00000708	3.5401e-6	2.8578	0.0664
Error	52	0.00006441	1.2387e-6		
C. Total	54	0.00007150			

Means for Oneway Anova

Level	Number	Mean	Std Error	Lower 95%	Upper 95%
high-performing	14	0.000102	0.00030	-0.0005	0.00070
low-performing	14	0.000873	0.00030	0.00028	0.00147
mid-performing	27	0.000026	0.00021	-0.0004	0.00046

Std Error uses a pooled estimate of error variance

Nonparametric Comparisons For All Pairs Using Steel-Dwass Method

q*	Alpha
2.34370	0.05

Level	- Level	Score Mean Difference	Std Err Dif	Z	p-Value	Hodges-Lehmann	Lower CL	Upper CL	Difference Plot
low-performing	high-performing	7.21429	3.109126	2.32036	0.0530	2.0077e-6	-6.293e-8	0.0000126	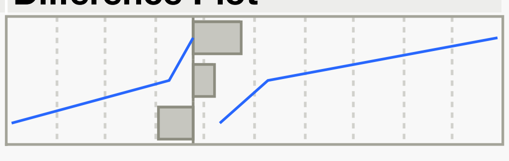
mid-performing	high-performing	5.09788	3.945055	1.29222	0.3995	8.6649e-7	-0.000001	0.0000031	
mid-performing	low-performing	-6.34524	3.945227	-1.60833	0.2421	-1.507e-6	-7.589e-6	0.0000011	

Excluded Rows 3

Oneway Analysis of Bedding materials By Herd Indicator=POCP kgNMVOCeq

Oneway Anova

Summary of Fit

Rsquare	0.195948
Adj Rsquare	0.165023
Root Mean Square Error	4.258e-5
Mean of Response	4.137e-5
Observations (or Sum Wgts)	55

Analysis of Variance

Source	DF	Sum of Squares	Mean Square	F Ratio	Prob > F
Herd	2	2.29735e-8	1.1487e-8	6.3362	0.0034*
Error	52	9.42691e-8	1.8129e-9		
C. Total	54	1.17243e-7			

Means for Oneway Anova

Level	Number	Mean	Std Error	Lower 95%	Upper 95%
high-performing	14	0.000022	1.14e-5	-8.6e-7	4.48e-5
low-performing	14	0.000075	1.14e-5	5.25e-5	0.0001
mid-performing	27	0.000034	8.19e-6	1.74e-5	0.00005

Std Error uses a pooled estimate of error variance

Nonparametric Comparisons For All Pairs Using Steel-Dwass Method

q*	Alpha
2.34370	0.05

Level	- Level	Score Mean Difference	Std Err Dif	Z	p-Value	Hodges-Lehmann	Lower CL	Upper CL	Difference Plot
low-performing	high-performing	8.50000	3.109126	2.73389	0.0172*	0.000029	3.9268e-6	0.0001056	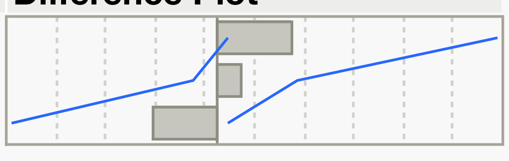
mid-performing	high-performing	4.55556	3.945055	1.15475	0.4804	8.7959e-6	-8.464e-6	0.0000306	
mid-performing	low-performing	-8.51455	3.945227	-2.15819	0.0785	-0.000023	-0.000077	0.0000041	

Excluded Rows 3
