# Supplementary material for: Climate Change and Photochemical Ozone Creation Potential Impact Indicators of Cow Milk: A Comparison of Different Scenarios for a Diet Assessment
Source: Animals (Basel). 2024 Jun 7;14(12):1725. doi: 10.3390/ani14121725 (PMC11201073; doi:10.3390/ani14121725)
Supplement: Supplementary file 1 [file animals-14-01725-s001.zip › animals-3004812-supplementary/Table 3/Anova of Enteric fermentation.pdf]

Oneway Analysis of Enteric fermentation By Herd Indicator=CC kgCO2eq

Oneway Anova

Summary of Fit

Rsquare	0.518683
Adj Rsquare	0.500171
Root Mean Square Error	0.065311
Mean of Response	0.716395
Observations (or Sum Wgts)	55

Analysis of Variance

Source	DF	Sum of Squares	Mean Square	F Ratio	Prob > F
Herd	2	0.23902947	0.119515	28.0184	<.0001*
Error	52	0.22180994	0.004266		
C. Total	54	0.46083941			

Means for Oneway Anova

Level	Number	Mean	Std Error	Lower 95%	Upper 95%
high-performing	14	0.629569	0.01746	0.59454	0.66460
low-performing	14	0.813746	0.01746	0.77872	0.84877
mid-performing	27	0.710938	0.01257	0.68572	0.73616

Std Error uses a pooled estimate of error variance

Means Comparisons

Comparisons for all pairs using Tukey-Kramer HSD

Confidence Quantile

q*	Alpha
2.41260	0.05

HSD Threshold Matrix

Abs(Dif)-HSD

	low-performing	mid-performing	high-performing
low-performing	-0.05956	0.05091	0.12462
mid-performing	0.05091	-0.04289	0.02947
high-performing	0.12462	0.02947	-0.05956

Positive values show pairs of means that are significantly different.

Connecting Letters Report

Level				Mean
low-performing	A			0.81374644
mid-performing		B		0.71093759
high-performing			C	0.62956879

Levels not connected by same letter are significantly different.

Ordered Differences Report

Level	- Level	Difference	Std Err Dif	Lower CL	Upper CL	p-Value
low-performing	high-performing	0.1841777	0.0246854	0.1246217	0.2437336	<.0001*
low-performing	mid-performing	0.1028089	0.0215097	0.0509145	0.1547032	<.0001*
mid-performing	high-performing	0.0813688	0.0215097	0.0294745	0.1332631	0.0012*

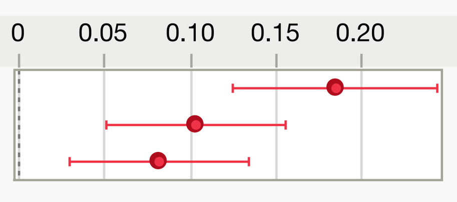

Excluded Rows 3

Oneway Analysis of Enteric fermentation By Herd Indicator=CC-biogenic kgCO2eq

Oneway Anova

Summary of Fit

Rsquare	0.518683
Adj Rsquare	0.500171
Root Mean Square Error	0.065311
Mean of Response	0.716395
Observations (or Sum Wgts)	55

Analysis of Variance

Source	DF	Sum of Squares	Mean Square	F Ratio	Prob > F
Herd	2	0.23902947	0.119515	28.0184	<.0001*
Error	52	0.22180994	0.004266		
C. Total	54	0.46083941			

Means for Oneway Anova

Level	Number	Mean	Std Error	Lower 95%	Upper 95%
high-performing	14	0.629569	0.01746	0.59454	0.66460
low-performing	14	0.813746	0.01746	0.77872	0.84877
mid-performing	27	0.710938	0.01257	0.68572	0.73616

Std Error uses a pooled estimate of error variance

Means Comparisons

Comparisons for all pairs using Tukey-Kramer HSD

Confidence Quantile

q*	Alpha
2.41260	0.05

HSD Threshold Matrix

Abs(Dif)-HSD

	low-performing	mid-performing	high-performing
low-performing	-0.05956	0.05091	0.12462
mid-performing	0.05091	-0.04289	0.02947
high-performing	0.12462	0.02947	-0.05956

Positive values show pairs of means that are significantly different.

Connecting Letters Report

Level				Mean
low-performing	A			0.81374644
mid-performing		B		0.71093759
high-performing			C	0.62956879

Levels not connected by same letter are significantly different.

Ordered Differences Report

Level	- Level	Difference	Std Err Dif	Lower CL	Upper CL	p-Value
low-performing	high-performing	0.1841777	0.0246854	0.1246217	0.2437336	<.0001*
low-performing	mid-performing	0.1028089	0.0215097	0.0509145	0.1547032	<.0001*
mid-performing	high-performing	0.0813688	0.0215097	0.0294745	0.1332631	0.0012*

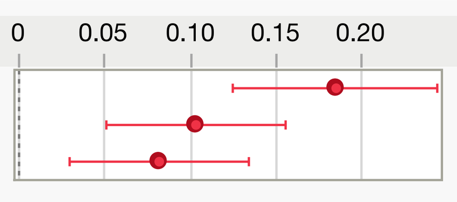

Excluded Rows 3

Oneway Analysis of Enteric fermentation By Herd Indicator=CC-fossil kgCO2eq

Oneway Anova

Summary of Fit

Rsquare	.
Adj Rsquare	.
Root Mean Square Error	0
Mean of Response	0
Observations (or Sum Wgts)	55

Analysis of Variance

Source	DF	Sum of Squares	Mean Square	F Ratio	Prob > F
Herd	2	0	0	.	.
Error	52	0	0		
C. Total	54	0			

Means for Oneway Anova

Level	Number	Mean	Std Error	Lower 95%	Upper 95%
high-performing	14	0	0	0	0
low-performing	14	0	0	0	0
mid-performing	27	0	0	0	0

Std Error uses a pooled estimate of error variance

Means Comparisons

Comparisons for all pairs using Tukey-Kramer HSD

Confidence Quantile

q*	Alpha
2.41260	0.05

HSD Threshold Matrix

Abs(Dif)-HSD

	high-performing	low-performing	mid-performing
high-performing	0	0	0
low-performing	0	0	0
mid-performing	0	0	0

Positive values show pairs of means that are significantly different.

Connecting Letters Report

Level	Mean
high-performing	0
low-performing	0
mid-performing	0

Levels not connected by same letter are significantly different.

Ordered Differences Report

Level	- Level	Difference	Std Err Dif	Lower CL	Upper CL	p-Value
low-performing	high-performing	0	0	0	0	.
mid-performing	high-performing	0	0	0	0	.
mid-performing	low-performing	0	0	0	0	.

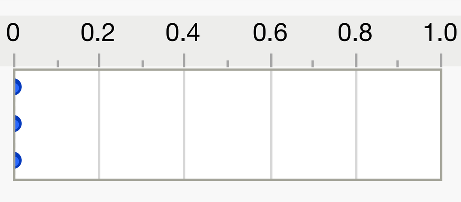

Excluded Rows 3

Oneway Analysis of Enteric fermentation By Herd Indicator=CC-LTU kgCO2eq

Oneway Anova

Summary of Fit

Rsquare	.
Adj Rsquare	.
Root Mean Square Error	0
Mean of Response	0
Observations (or Sum Wgts)	55

Analysis of Variance

Source	DF	Sum of Squares	Mean Square	F Ratio	Prob > F
Herd	2	0	0	.	.
Error	52	0	0		
C. Total	54	0			

Means for Oneway Anova

Level	Number	Mean	Std Error	Lower 95%	Upper 95%
high-performing	14	0	0	0	0
low-performing	14	0	0	0	0
mid-performing	27	0	0	0	0

Std Error uses a pooled estimate of error variance

Means Comparisons

Comparisons for all pairs using Tukey-Kramer HSD

Confidence Quantile

q*	Alpha
2.41260	0.05

HSD Threshold Matrix

Abs(Dif)-HSD

	high-performing	low-performing	mid-performing
high-performing	0	0	0
low-performing	0	0	0
mid-performing	0	0	0

Positive values show pairs of means that are significantly different.

Connecting Letters Report

Level	Mean
high-performing	0
low-performing	0
mid-performing	0

Levels not connected by same letter are significantly different.

Ordered Differences Report

Level	- Level	Difference	Std Err Dif	Lower CL	Upper CL	p-Value
low-performing	high-performing	0	0	0	0	.
mid-performing	high-performing	0	0	0	0	.
mid-performing	low-performing	0	0	0	0	.

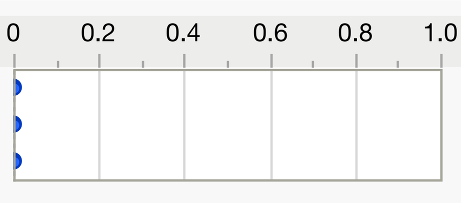

Excluded Rows 3

Oneway Analysis of Enteric fermentation By Herd Indicator=POCP kgNMVOCeq

Oneway Anova

Summary of Fit

Rsquare	0.518683
Adj Rsquare	0.500171
Root Mean Square Error	1.94e-5
Mean of Response	0.000213
Observations (or Sum Wgts)	55

Analysis of Variance

Source	DF	Sum of Squares	Mean Square	F Ratio	Prob > F
Herd	2	2.10929e-8	1.0546e-8	28.0184	<.0001*
Error	52	1.95734e-8	3.764e-10		
C. Total	54	4.06663e-8			

Means for Oneway Anova

Level	Number	Mean	Std Error	Lower 95%	Upper 95%
high-performing	14	0.000187	5.1852e-6	0.00018	0.00020
low-performing	14	0.000242	5.1852e-6	0.00023	0.00025
mid-performing	27	0.000211	3.7338e-6	0.00020	0.00022

Std Error uses a pooled estimate of error variance

Means Comparisons

Comparisons for all pairs using Tukey-Kramer HSD

Confidence Quantile

q*	Alpha
2.41260	0.05

HSD Threshold Matrix

Abs(Dif)-HSD

	low-performing	mid-performing	high-performing
low-performing	-0.00002	0.00002	0.00004
mid-performing	0.00002	-0.00001	0.00001
high-performing	0.00004	0.00001	-0.00002

Positive values show pairs of means that are significantly different.

Connecting Letters Report

Level				Mean
low-performing	A			0.00024173
mid-performing		B		0.00021119
high-performing			C	0.00018702

Levels not connected by same letter are significantly different.

Ordered Differences Report

Level	- Level	Difference	Std Err Dif	Lower CL	Upper CL	p-Value
low-performing	high-performing	0.0000547	7.333e-6	0.0000370	0.0000724	<.0001*
low-performing	mid-performing	0.0000305	6.3897e-6	0.0000151	0.0000460	<.0001*
mid-performing	high-performing	0.0000242	6.3897e-6	0.0000088	0.0000396	0.0012*

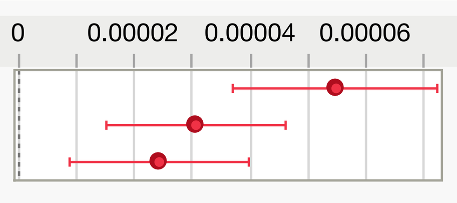

Excluded Rows 3
