# Supplementary material for: Climate Change and Photochemical Ozone Creation Potential Impact Indicators of Cow Milk: A Comparison of Different Scenarios for a Diet Assessment
Source: Animals (Basel). 2024 Jun 7;14(12):1725. doi: 10.3390/ani14121725 (PMC11201073; doi:10.3390/ani14121725)
Supplement: Supplementary file 1 [file animals-14-01725-s001.zip › animals-3004812-supplementary/Table 3/Anova of In-farm feeds.pdf]

Oneway Analysis of In-farm feeds By Herd Indicator=CC kgCO2eq

Oneway Anova

Summary of Fit

Rsquare	0.247354
Adj Rsquare	0.218407
Root Mean Square Error	0.036292
Mean of Response	0.087408
Observations (or Sum Wgts)	55

Analysis of Variance

Source	DF	Sum of Squares	Mean Square	F Ratio	Prob > F
Herd	2	0.02250921	0.011255	8.5448	0.0006*
Error	52	0.06849061	0.001317		
C. Total	54	0.09099982			

Means for Oneway Anova

Level	Number	Mean	Std Error	Lower 95%	Upper 95%
high-performing	14	0.059089	0.00970	0.03963	0.07855
low-performing	14	0.115795	0.00970	0.09633	0.13526
mid-performing	27	0.087373	0.00698	0.07336	0.10139

Std Error uses a pooled estimate of error variance

Nonparametric Comparisons For All Pairs Using Steel-Dwass Method

q*	Alpha
2.34370	0.05

Level	- Level	Score Mean Difference	Std Err Dif	Z	p-Value	Hodges-Lehmann	Lower CL	Upper CL	Difference Plot
low-performing	high-performing	12.9286	3.109126	4.15827	<.0001*	0.052752	0.032166	0.078242	
mid-performing	high-performing	10.1415	3.945227	2.57058	0.0274*	0.020714	0.000870	0.042638	
mid-performing	low-performing	-12.2024	3.945227	-3.09295	0.0056*	-0.031441	-0.053724	-0.010453	

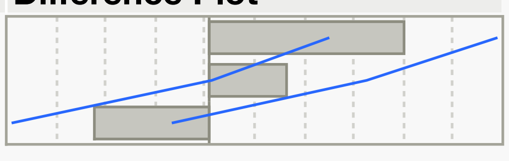

Excluded Rows 3

Oneway Analysis of In-farm feeds By Herd Indicator=CC-biogenic kgCO2eq

Oneway Anova

Summary of Fit

Rsquare	0.095632
Adj Rsquare	0.060848
Root Mean Square Error	8.504e-6
Mean of Response	7.329e-6
Observations (or Sum Wgts)	55

Analysis of Variance

Source	DF	Sum of Squares	Mean Square	F Ratio	Prob > F
Herd	2	3.9769e-10	1.988e-10	2.7493	0.0733
Error	52	3.76091e-9	7.233e-11		
C. Total	54	4.1586e-9			

Means for Oneway Anova

Level	Number	Mean	Std Error	Lower 95%	Upper 95%
high-performing	14	3.368e-6	2.2729e-6	-1.193e-6	7.93e-6
low-performing	14	0.000011	2.2729e-6	6.3217e-6	1.54e-5
mid-performing	27	7.54e-6	1.6367e-6	4.2558e-6	0.00001

Std Error uses a pooled estimate of error variance

Nonparametric Comparisons For All Pairs Using Steel-Dwass Method

q*	Alpha
2.34370	0.05

Level	- Level	Score Mean Difference	Std Err Dif	Z	p-Value	Hodges-Lehmann	Lower CL	Upper CL	Difference Plot
low-performing	high-performing	12.5000	3.108701	4.02097	0.0002*	6.8844e-6	3.5844e-6	0.000011	
mid-performing	high-performing	5.9114	3.943508	1.49901	0.2914	1.6871e-6	-8.46e-7	4.8156e-6	
mid-performing	low-performing	-12.4193	3.945055	-3.14807	0.0047*	-5.308e-6	-9.307e-6	-1.659e-6	

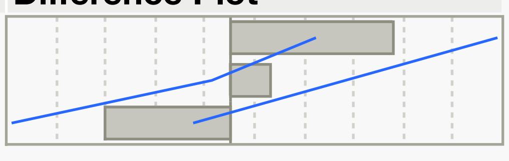

Excluded Rows 3

Oneway Analysis of In-farm feeds By Herd Indicator=CC-fossil kgCO2eq

Oneway Anova

Summary of Fit

Rsquare	0.247366
Adj Rsquare	0.218419
Root Mean Square Error	0.036268
Mean of Response	0.087371
Observations (or Sum Wgts)	55

Analysis of Variance

Source	DF	Sum of Squares	Mean Square	F Ratio	Prob > F
Herd	2	0.02248019	0.011240	8.5453	0.0006*
Error	52	0.06839803	0.001315		
C. Total	54	0.09087822			

Means for Oneway Anova

Level	Number	Mean	Std Error	Lower 95%	Upper 95%
high-performing	14	0.059067	0.00969	0.03962	0.07852
low-performing	14	0.115736	0.00969	0.09629	0.13519
mid-performing	27	0.087339	0.00698	0.07333	0.10134

Std Error uses a pooled estimate of error variance

Nonparametric Comparisons For All Pairs Using Steel-Dwass Method

q*	Alpha
2.34370	0.05

Level	- Level	Score Mean Difference	Std Err Dif	Z	p-Value	Hodges-Lehmann	Lower CL	Upper CL	Difference Plot
low-performing	high-performing	12.9286	3.109126	4.15827	<.0001*	0.052722	0.032125	0.078221	
mid-performing	high-performing	10.1415	3.945227	2.57058	0.0274*	0.020694	0.000846	0.042632	
mid-performing	low-performing	-12.0939	3.945227	-3.06546	0.0062*	-0.031409	-0.053625	-0.010411	

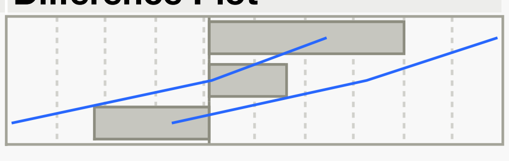

Excluded Rows 3

Oneway Analysis of In-farm feeds By Herd Indicator=CC-LTU kgCO2eq

Oneway Anova

Summary of Fit

Rsquare	0.171827
Adj Rsquare	0.139974
Root Mean Square Error	2.447e-5
Mean of Response	3.034e-5
Observations (or Sum Wgts)	55

Analysis of Variance

Source	DF	Sum of Squares	Mean Square	F Ratio	Prob > F
Herd	2	6.45762e-9	3.2288e-9	5.3944	0.0074*
Error	52	3.11244e-8	5.985e-10		
C. Total	54	3.7582e-8			

Means for Oneway Anova

Level	Number	Mean	Std Error	Lower 95%	Upper 95%
high-performing	14	0.000019	6.5386e-6	5.82e-6	3.21e-5
low-performing	14	0.000048	6.5386e-6	3.49e-5	6.11e-5
mid-performing	27	0.000027	4.7083e-6	1.77e-5	3.66e-5

Std Error uses a pooled estimate of error variance

Nonparametric Comparisons For All Pairs Using Steel-Dwass Method

q*	Alpha
2.34370	0.05

Level	- Level	Score Mean Difference	Std Err Dif	Z	p-Value	Hodges-Lehmann	Lower CL	Upper CL	Difference Plot
low-performing	high-performing	10.0714	3.108701	3.23975	0.0034*	0.000024	6.8188e-6	0.000052	
mid-performing	high-performing	3.7421	3.943508	0.94892	0.6093	5.5357e-6	-8.737e-6	0.000022	
mid-performing	low-performing	-10.0331	3.945055	-2.54320	0.0296*	-0.000020	-0.000041	-1.356e-6	

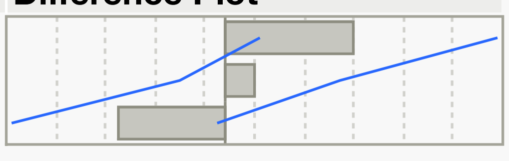

Excluded Rows 3

Oneway Analysis of In-farm feeds By Herd Indicator=POCP kgNMVOCeq

Oneway Anova

Summary of Fit

Rsquare	0.0793
Adj Rsquare	0.043888
Root Mean Square Error	0.000035
Mean of Response	0.000026
Observations (or Sum Wgts)	55

Analysis of Variance

Source	DF	Sum of Squares	Mean Square	F Ratio	Prob > F
Herd	2	5.50727e-9	2.7536e-9	2.2394	0.1167
Error	52	6.39414e-8	1.2296e-9		
C. Total	54	6.94487e-8			

Means for Oneway Anova

Level	Number	Mean	Std Error	Lower 95%	Upper 95%
high-performing	14	0.000011	9.3719e-6	-8.1e-6	0.00003
low-performing	14	0.000038	9.3719e-6	0.00002	5.73e-5
mid-performing	27	0.000027	6.7485e-6	1.38e-5	0.00004

Std Error uses a pooled estimate of error variance

Nonparametric Comparisons For All Pairs Using Steel-Dwass Method

q*	Alpha
2.34370	0.05

Level	- Level	Score Mean Difference	Std Err Dif	Z	p-Value	Hodges-Lehmann	Lower CL	Upper CL	Difference Plot
low-performing	high-performing	12.7857	3.108701	4.11288	0.0001*	0.000026	0.000012	0.000042	
mid-performing	high-performing	5.6944	3.943508	1.44400	0.3183	6.745e-6	-3.051e-6	0.000016	
mid-performing	low-performing	-12.4193	3.945055	-3.14807	0.0047*	-0.000021	-0.000034	-7.151e-6	

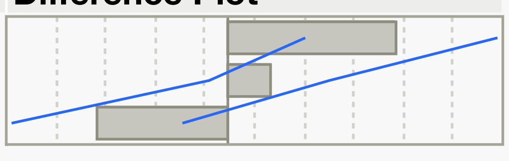

Excluded Rows 3
