# Supplementary material for: Climate Change and Photochemical Ozone Creation Potential Impact Indicators of Cow Milk: A Comparison of Different Scenarios for a Diet Assessment
Source: Animals (Basel). 2024 Jun 7;14(12):1725. doi: 10.3390/ani14121725 (PMC11201073; doi:10.3390/ani14121725)
Supplement: Supplementary file 1 [file animals-14-01725-s001.zip › animals-3004812-supplementary/Table 3/Anova of Manure handling.pdf]

Oneway Analysis of Manure handling By Herd Indicator=CC kgCO2eq

Oneway Anova

Summary of Fit

Rsquare	0.059549
Adj Rsquare	0.023378
Root Mean Square Error	0.131739
Mean of Response	0.431349
Observations (or Sum Wgts)	55

Analysis of Variance

Source	DF	Sum of Squares	Mean Square	F Ratio	Prob > F
Herd	2	0.05714451	0.028572	1.6463	0.2026
Error	52	0.90247519	0.017355		
C. Total	54	0.95961970			

Means for Oneway Anova

Level	Number	Mean	Std Error	Lower 95%	Upper 95%
high-performing	14	0.376791	0.03521	0.30614	0.44744
low-performing	14	0.457625	0.03521	0.38697	0.52828
mid-performing	27	0.446014	0.02535	0.39514	0.49689

Std Error uses a pooled estimate of error variance

Means Comparisons

Comparisons for all pairs using Tukey-Kramer HSD

Confidence Quantile

q*	Alpha
2.41260	0.05

HSD Threshold Matrix

Abs(Dif)-HSD

	low-performing	mid-performing	high-performing
low-performing	-0.12013	-0.09306	-0.03930
mid-performing	-0.09306	-0.08650	-0.03545
high-performing	-0.03930	-0.03545	-0.12013

Positive values show pairs of means that are significantly different.

Connecting Letters Report

Level		Mean
low-performing	A	0.45762513
mid-performing	A	0.44601369
high-performing	A	0.37679105

Levels not connected by same letter are significantly different.

Ordered Differences Report

Level	- Level	Difference	Std Err Dif	Lower CL	Upper CL	p-Value
low-performing	high-performing	0.0808341	0.0497928	-0.039296	0.2009642	0.2450
mid-performing	high-performing	0.0692226	0.0433872	-0.035453	0.1738986	0.2567
low-performing	mid-performing	0.0116114	0.0433872	-0.093065	0.1162874	0.9613

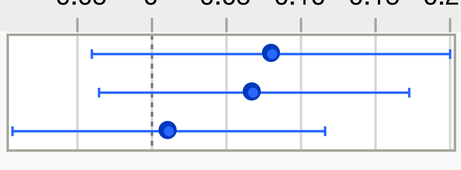

Excluded Rows 3

Oneway Analysis of Manure handling By Herd Indicator=CC-biogenic kgCO2eq

Oneway Anova

Summary of Fit

Rsquare	0.03283
Adj Rsquare	-0.00437
Root Mean Square Error	0.130998
Mean of Response	0.371273
Observations (or Sum Wgts)	55

Analysis of Variance

Source	DF	Sum of Squares	Mean Square	F Ratio	Prob > F
Herd	2	0.03028988	0.015145	0.8825	0.4198
Error	52	0.89235139	0.017161		
C. Total	54	0.92264127			

Means for Oneway Anova

Level	Number	Mean	Std Error	Lower 95%	Upper 95%
high-performing	14	0.331436	0.03501	0.26118	0.40169
low-performing	14	0.380094	0.03501	0.30984	0.45035
mid-performing	27	0.387355	0.02521	0.33677	0.43794

Std Error uses a pooled estimate of error variance

Means Comparisons

Comparisons for all pairs using Tukey-Kramer HSD

Confidence Quantile

q*	Alpha
2.41260	0.05

HSD Threshold Matrix

Abs(Dif)-HSD

	mid-performing	low-performing	high-performing
mid-performing	-0.08602	-0.09683	-0.04817
low-performing	-0.09683	-0.11945	-0.07080
high-performing	-0.04817	-0.07080	-0.11945

Positive values show pairs of means that are significantly different.

Connecting Letters Report

Level		Mean
mid-performing	A	0.38735502
low-performing	A	0.38009356
high-performing	A	0.33143619

Levels not connected by same letter are significantly different.

Ordered Differences Report

Level	- Level	Difference	Std Err Dif	Lower CL	Upper CL	p-Value
mid-performing	high-performing	0.0559188	0.0431432	-0.048168	0.1600061	0.4037
low-performing	high-performing	0.0486574	0.0495128	-0.070797	0.1681118	0.5910
mid-performing	low-performing	0.0072615	0.0431432	-0.096826	0.1113487	0.9845

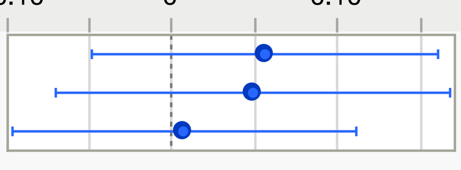

Excluded Rows 3

Oneway Analysis of Manure handling By Herd Indicator=CC-fossil kgCO2eq

Oneway Anova

Summary of Fit

Rsquare	0.664977
Adj Rsquare	0.652091
Root Mean Square Error	0.008441
Mean of Response	0.060076
Observations (or Sum Wgts)	55

Analysis of Variance

Source	DF	Sum of Squares	Mean Square	F Ratio	Prob > F
Herd	2	0.00735396	0.003677	51.6065	<.0001*
Error	52	0.00370501	0.000071		
C. Total	54	0.01105898			

Means for Oneway Anova

Level	Number	Mean	Std Error	Lower 95%	Upper 95%
high-performing	14	0.045355	0.00226	0.04083	0.04988
low-performing	14	0.077532	0.00226	0.07300	0.08206
mid-performing	27	0.058659	0.00162	0.05540	0.06192

Std Error uses a pooled estimate of error variance

Nonparametric Comparisons For All Pairs Using Steel-Dwass Method

q*	Alpha
2.34370	0.05

Level	- Level	Score Mean Difference	Std Err Dif	Z	p-Value	Hodges-Lehmann	Lower CL	Upper CL	Difference Plot
mid-performing	high-performing	18.1680	3.945227	4.60506	<.0001*	0.011632	0.005972	0.019980	
low-performing	high-performing	13.5000	3.109126	4.34206	<.0001*	0.032414	0.020338	0.040947	
mid-performing	low-performing	-17.5172	3.945227	-4.44010	<.0001*	-0.018038	-0.025703	-0.011929	

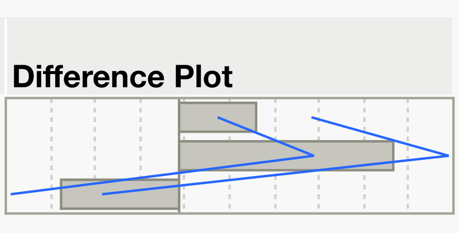

Excluded Rows 3

Oneway Analysis of Manure handling By Herd Indicator=CC-LTU kgCO2eq

Oneway Anova

Summary of Fit

Rsquare	.
Adj Rsquare	.
Root Mean Square Error	0
Mean of Response	0
Observations (or Sum Wgts)	55

Analysis of Variance

Source	DF	Sum of Squares	Mean Square	F Ratio	Prob > F
Herd	2	0	0	.	.
Error	52	0	0		
C. Total	54	0			

Means for Oneway Anova

Level	Number	Mean	Std Error	Lower 95%	Upper 95%
high-performing	14	0	0	0	0
low-performing	14	0	0	0	0
mid-performing	27	0	0	0	0

Std Error uses a pooled estimate of error variance

Excluded Rows 3

Oneway Analysis of Manure handling By Herd Indicator=POCP kgNMVOCeq

Oneway Anova

Summary of Fit

Rsquare	0.03283
Adj Rsquare	-0.00437
Root Mean Square Error	0.000039
Mean of Response	0.00011
Observations (or Sum Wgts)	55

Analysis of Variance

Source	DF	Sum of Squares	Mean Square	F Ratio	Prob > F
Herd	2	2.6729e-9	1.3364e-9	0.8825	0.4198
Error	52	7.87446e-8	1.5143e-9		
C. Total	54	8.14175e-8			

Means for Oneway Anova

Level	Number	Mean	Std Error	Lower 95%	Upper 95%
high-performing	14	0.000098	0.00001	7.76e-5	0.00012
low-performing	14	0.000113	0.00001	9.2e-5	0.00013
mid-performing	27	0.000115	7.49e-6	0.00010	0.00013

Std Error uses a pooled estimate of error variance

Means Comparisons

Comparisons for all pairs using Tukey-Kramer HSD

Confidence Quantile

q*	Alpha
2.41260	0.05

HSD Threshold Matrix

Abs(Dif)-HSD

	mid-performing	low-performing	high-performing
mid-performing	-0.00003	-0.00003	-0.00001
low-performing	-0.00003	-0.00004	-0.00002
high-performing	-0.00001	-0.00002	-0.00004

Positive values show pairs of means that are significantly different.

Connecting Letters Report

Level		Mean
mid-performing	A	0.00011507
low-performing	A	0.00011291
high-performing	A	0.00009846

Levels not connected by same letter are significantly different.

Ordered Differences Report

Level	- Level	Difference	Std Err Dif	Lower CL	Upper CL	p-Value
mid-performing	high-performing	0.0000166	0.0000128	-0.000014	0.0000475	0.4037
low-performing	high-performing	0.0000145	0.0000147	-0.000021	0.0000499	0.5910
mid-performing	low-performing	0.0000022	0.0000128	-0.000029	0.0000331	0.9845

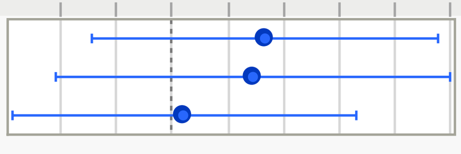

Excluded Rows 3
