# Supplementary material for: Climate Change and Photochemical Ozone Creation Potential Impact Indicators of Cow Milk: A Comparison of Different Scenarios for a Diet Assessment
Source: Animals (Basel). 2024 Jun 7;14(12):1725. doi: 10.3390/ani14121725 (PMC11201073; doi:10.3390/ani14121725)
Supplement: Supplementary file 1 [file animals-14-01725-s001.zip › animals-3004812-supplementary/Table 3/Anova of Total.pdf]

Oneway Analysis of Total By Herd Indicator=CC kgCO2eq

Oneway Anova

Summary of Fit

Rsquare	0.39182
Adj Rsquare	0.368429
Root Mean Square Error	0.249006
Mean of Response	1.915548
Observations (or Sum Wgts)	55

Analysis of Variance

Source	DF	Sum of Squares	Mean Square	F Ratio	Prob > F
Herd	2	2.0771969	1.03860	16.7505	<.0001*
Error	52	3.2242030	0.06200		
C. Total	54	5.3014000			

Means for Oneway Anova

Level	Number	Mean	Std Error	Lower 95%	Upper 95%
high-performing	14	1.63013	0.06655	1.4966	1.7637
low-performing	14	2.17346	0.06655	2.0399	2.3070
mid-performing	27	1.92981	0.04792	1.8336	2.0260

Std Error uses a pooled estimate of error variance

Means Comparisons

Comparisons for all pairs using Tukey-Kramer HSD

Confidence Quantile

q*	Alpha
2.41260	0.05

HSD Threshold Matrix

Abs(Dif)-HSD

	low-performing	mid-performing	high-performing
low-performing	-0.22706	0.04580	0.31626
mid-performing	0.04580	-0.16350	0.10182
high-performing	0.31626	0.10182	-0.22706

Positive values show pairs of means that are significantly different.

Connecting Letters Report

Level				Mean
low-performing	A			2.1734597
mid-performing		B		1.9298081
high-performing			C	1.6301349

Levels not connected by same letter are significantly different.

Ordered Differences Report

Level	- Level	Difference	Std Err Dif	Lower CL	Upper CL	p-Value
low-performing	high-performing	0.5433247	0.0941154	0.3162621	0.7703873	<.0001*
mid-performing	high-performing	0.2996732	0.0820079	0.1018211	0.4975253	0.0017*
low-performing	mid-performing	0.2436515	0.0820079	0.0457994	0.4415036	0.0123*

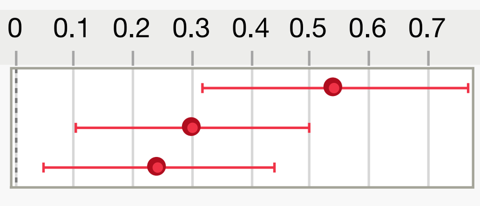

Excluded Rows 3

Oneway Analysis of Total By Herd Indicator=CC-biogenic kgCO2eq

Oneway Anova

Summary of Fit

Rsquare	0.245587
Adj Rsquare	0.216571
Root Mean Square Error	0.159237
Mean of Response	1.101451
Observations (or Sum Wgts)	55

Analysis of Variance

Source	DF	Sum of Squares	Mean Square	F Ratio	Prob > F
Herd	2	0.4292295	0.214615	8.4639	0.0007*
Error	52	1.3185407	0.025357		
C. Total	54	1.7477703			

Means for Oneway Anova

Level	Number	Mean	Std Error	Lower 95%	Upper 95%
high-performing	14	0.97072	0.04256	0.8853	1.0561
low-performing	14	1.21745	0.04256	1.1320	1.3028
mid-performing	27	1.10909	0.03065	1.0476	1.1706

Std Error uses a pooled estimate of error variance

Means Comparisons

Comparisons for all pairs using Tukey-Kramer HSD

Confidence Quantile

q*	Alpha
2.41260	0.05

HSD Threshold Matrix

Abs(Dif)-HSD

	low-performing	mid-performing	high-performing
low-performing	-0.14520	-0.01817	0.10153
mid-performing	-0.01817	-0.10456	0.01185
high-performing	0.10153	0.01185	-0.14520

Positive values show pairs of means that are significantly different.

Connecting Letters Report

Level			Mean
low-performing	A		1.2174477
mid-performing	A		1.1090927
high-performing		B	0.9707169

Levels not connected by same letter are significantly different.

Ordered Differences Report

Level	- Level	Difference	Std Err Dif	Lower CL	Upper CL	p-Value
low-performing	high-performing	0.2467308	0.0601861	0.101526	0.3919356	0.0004*
mid-performing	high-performing	0.1383757	0.0524434	0.011851	0.2649007	0.0290*
low-performing	mid-performing	0.1083550	0.0524434	-0.018170	0.2348800	0.1069

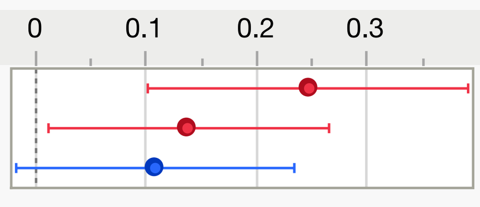

Excluded Rows 3

Oneway Analysis of Total By Herd Indicator=CC-fossil kgCO2eq

Oneway Anova

Summary of Fit

Rsquare	0.218808
Adj Rsquare	0.188762
Root Mean Square Error	0.120747
Mean of Response	0.517967
Observations (or Sum Wgts)	55

Analysis of Variance

Source	DF	Sum of Squares	Mean Square	F Ratio	Prob > F
Herd	2	0.21235587	0.106178	7.2825	0.0016*
Error	52	0.75815634	0.014580		
C. Total	54	0.97051221			

Means for Oneway Anova

Level	Number	Mean	Std Error	Lower 95%	Upper 95%
high-performing	14	0.419991	0.03227	0.35523	0.48475
low-performing	14	0.590266	0.03227	0.52551	0.65502
mid-performing	27	0.531281	0.02324	0.48465	0.57791

Std Error uses a pooled estimate of error variance

Nonparametric Comparisons For All Pairs Using Steel-Dwass Method

q*	Alpha
2.34370	0.05

Level	- Level	Score Mean Difference	Std Err Dif	Z	p-Value	Hodges-Lehmann	Lower CL	Upper CL	Difference Plot
low-performing	high-performing	10.0714	3.109126	3.23931	0.0034*	0.172414	0.049017	0.2811023	
mid-performing	high-performing	9.8161	3.945227	2.48810	0.0343*	0.107899	0.011203	0.1945404	
mid-performing	low-performing	-6.8876	3.945227	-1.74580	0.1883	-0.068349	-0.151374	0.0192919	

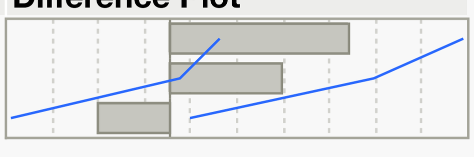

Excluded Rows 3

Oneway Analysis of Total By Herd Indicator=CC-LTU kgCO2eq

Oneway Anova

Summary of Fit

Rsquare	0.156572
Adj Rsquare	0.124133
Root Mean Square Error	0.108707
Mean of Response	0.29613
Observations (or Sum Wgts)	55

Analysis of Variance

Source	DF	Sum of Squares	Mean Square	F Ratio	Prob > F
Herd	2	0.11407344	0.057037	4.8266	0.0119*
Error	52	0.61449467	0.011817		
C. Total	54	0.72856811			

Means for Oneway Anova

Level	Number	Mean	Std Error	Lower 95%	Upper 95%
high-performing	14	0.239427	0.02905	0.18113	0.29773
low-performing	14	0.365746	0.02905	0.30745	0.42405
mid-performing	27	0.289434	0.02092	0.24745	0.33141

Std Error uses a pooled estimate of error variance

Nonparametric Comparisons For All Pairs Using Steel-Dwass Method

q*	Alpha
2.34370	0.05

Level	- Level	Score Mean Difference	Std Err Dif	Z	p-Value	Hodges-Lehmann	Lower CL	Upper CL	Difference Plot
low-performing	high-performing	8.64286	3.109126	2.77983	0.0150*	0.107928	0.020132	0.2306966	
mid-performing	high-performing	8.08069	3.945227	2.04822	0.1009	0.055024	-0.016180	0.1181849	
mid-performing	low-performing	-7.32143	3.945227	-1.85577	0.1518	-0.062138	-0.154597	0.0232711	

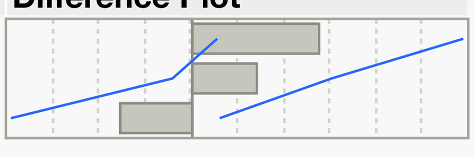

Excluded Rows 3

Oneway Analysis of Total By Herd Indicator=POCP kgNMVOCeq

Oneway Anova

Summary of Fit

Rsquare	0.626843
Adj Rsquare	0.612491
Root Mean Square Error	0.001219
Mean of Response	0.008837
Observations (or Sum Wgts)	55

Analysis of Variance

Source	DF	Sum of Squares	Mean Square	F Ratio	Prob > F
Herd	2	0.00012985	0.000065	43.6758	<.0001*
Error	52	0.00007730	1.487e-6		
C. Total	54	0.00020716			

Means for Oneway Anova

Level	Number	Mean	Std Error	Lower 95%	Upper 95%
high-performing	14	0.006884	0.00033	0.00623	0.00754
low-performing	14	0.011159	0.00033	0.01051	0.01181
mid-performing	27	0.008646	0.00023	0.00817	0.00912

Std Error uses a pooled estimate of error variance

Nonparametric Comparisons For All Pairs Using Steel-Dwass Method

q*	Alpha
2.34370	0.05

Level	- Level	Score Mean Difference	Std Err Dif	Z	p-Value	Hodges-Lehmann	Lower CL	Upper CL	Difference Plot
mid-performing	high-performing	15.6733	3.945227	3.97272	0.0002*	0.001466	0.000593	0.002803	
low-performing	high-performing	13.5000	3.109126	4.34206	<.0001*	0.004361	0.002644	0.005817	
mid-performing	low-performing	-16.8664	3.945227	-4.27514	<.0001*	-0.002558	-0.003793	-0.001430	

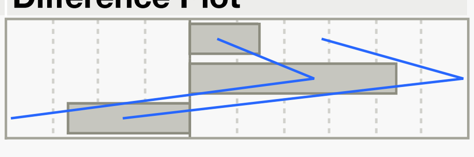

Excluded Rows 3
