# Supplementary material for: Climate Change and Photochemical Ozone Creation Potential Impact Indicators of Cow Milk: A Comparison of Different Scenarios for a Diet Assessment
Source: Animals (Basel). 2024 Jun 7;14(12):1725. doi: 10.3390/ani14121725 (PMC11201073; doi:10.3390/ani14121725)
Supplement: Supplementary file 1 [file animals-14-01725-s001.zip › animals-3004812-supplementary/Table 3/Distribution of Bedding materials.pdf]

Distributions Herd=high-performing, Indicator=CC kgCO2eq

Bedding materials

Compare Distributions

Show	Distribution		AICc ^	BIC	-2*LogLikelihood
☑	Normal		-88.84269	-88.65549	-93.9336

Summary Statistics

Mean	0.0082564
Std Dev	0.0087562
Std Err Mean	0.0023402
Upper 95% Mean	0.013312
Lower 95% Mean	0.0032007
N	14
N Missing	0

Fitted Normal Distribution

Parameter		Estimate	Std Error	Lower 95%	Upper 95%
Location	μ	0.0082564	0.0023402	0.0032007	0.013312
Dispersion	σ	0.0087562	0.0017512	0.0063478	0.0141066

Measures	
-2*LogLikelihood	-93.9336
AICc	-88.84269
BIC	-88.65549

Goodness-of-Fit Test

	W	Prob<W
Shapiro-Wilk	0.8385048	0.0156*

	A^2	Simulated p-Value
Anderson-Darling	0.7350427	0.0404*

Note: Ho = The data is from the Normal distribution. Small p-values reject Ho.

Distributions Herd=high-performing, Indicator=CC-biogenic kgCO2eq

Bedding materials

Compare Distributions

Show	Distribution		AICc ^	BIC	-2*LogLikelihood
☑	Normal		-327.7226	-327.5354	-332.8135

Summary Statistics

Mean	1.6435e-6
Std Dev	1.7265e-6
Std Err Mean	4.6142e-7
Upper 95% Mean	2.6404e-6
Lower 95% Mean	6.4668e-7
N	14
N Missing	0

Fitted Normal Distribution

Parameter		Estimate	Std Error	Lower 95%	Upper 95%
Location	μ	1.6435e-6	4.6142e-7	6.4668e-7	2.6404e-6
Dispersion	σ	1.7265e-6	3.453e-7	1.2516e-6	2.7814e-6

Measures	
-2*LogLikelihood	-332.8135
AICc	-327.7226
BIC	-327.5354

Goodness-of-Fit Test

	W	Prob<W
Shapiro-Wilk	0.8550904	0.0261*

	A^2	Simulated p-Value
Anderson-Darling	0.6542407	0.0704

Note: Ho = The data is from the Normal distribution. Small p-values reject Ho.

Distributions Herd=high-performing, Indicator=CC-fossil kgCO2eq

Bedding materials

Compare Distributions

Show	Distribution		AICc ^	BIC	-2*LogLikelihood
☑	Normal		-88.9624	-88.7752	-94.05331

Summary Statistics

Mean	0.0081529
Std Dev	0.0087188
Std Err Mean	0.0023302
Upper 95% Mean	0.013187
Lower 95% Mean	0.0031188
N	14
N Missing	0

Fitted Normal Distribution

Parameter		Estimate	Std Error	Lower 95%	Upper 95%
Location	μ	0.0081529	0.0023302	0.0031188	0.013187
Dispersion	σ	0.0087188	0.0017438	0.0063207	0.0140464

Measures	
-2*LogLikelihood	-94.05331
AICc	-88.9624
BIC	-88.7752

Goodness-of-Fit Test

	W	Prob<W
Shapiro-Wilk	0.8309954	0.0124*

	A^2	Simulated p-Value
Anderson-Darling	0.7785313	0.0336*

Note: Ho = The data is from the Normal distribution. Small p-values reject Ho.

Distributions Herd=high-performing, Indicator=CC-LTU kgCO2eq

Bedding materials

Compare Distributions

Show	Distribution		AICc ^	BIC	-2*LogLikelihood
☑	Normal		-177.0445	-176.8573	-182.1354

Summary Statistics

Mean	0.0001018
Std Dev	0.0003752
Std Err Mean	0.0001003
Upper 95% Mean	0.0003185
Lower 95% Mean	-0.000115
N	14
N Missing	0

Fitted Normal Distribution

Parameter		Estimate	Std Error	Lower 95%	Upper 95%
Location	μ	0.0001018	0.0001003	-0.000115	0.0003185
Dispersion	σ	0.0003752	7.5039e-5	0.000272	0.0006045

Measures	
-2*LogLikelihood	-182.1354
AICc	-177.0445
BIC	-176.8573

Goodness-of-Fit Test

	W	Prob<W
Shapiro-Wilk	0.3005323	<.0001*

	A^2	Simulated p-Value
Anderson-Darling	4.759732	<.0001*

Note: Ho = The data is from the Normal distribution. Small p-values reject Ho.

Distributions Herd=high-performing, Indicator=POCP kgNMVOCeq

Bedding materials

Compare Distributions

Show	Distribution		AICc ^	BIC	-2*LogLikelihood
☑	Normal		-253.4926	-253.3054	-258.5835

Summary Statistics

Mean	2.1971e-5
Std Dev	2.4463e-5
Std Err Mean	6.538e-6
Upper 95% Mean	0.0000361
Lower 95% Mean	7.8468e-6
N	14
N Missing	0

Fitted Normal Distribution

Parameter		Estimate	Std Error	Lower 95%	Upper 95%
Location	μ	2.1971e-5	6.538e-6	7.8468e-6	0.0000361
Dispersion	σ	2.4463e-5	4.8926e-6	1.7735e-5	3.9411e-5

Measures	
-2*LogLikelihood	-258.5835
AICc	-253.4926
BIC	-253.3054

Goodness-of-Fit Test

	W	Prob<W
Shapiro-Wilk	0.8150682	0.0077*

	A^2	Simulated p-Value
Anderson-Darling	0.8554661	0.0236*

Note: Ho = The data is from the Normal distribution. Small p-values reject Ho.

Distributions Herd=low-performing, Indicator=CC kgCO2eq

Bedding materials

Compare Distributions

Show	Distribution		AICc ^	BIC	-2*LogLikelihood
☑	Normal		-58.26275	-58.07554	-63.35366

Summary Statistics

Mean	0.028373
Std Dev	0.0260991
Std Err Mean	0.0069753
Upper 95% Mean	0.0434422
Lower 95% Mean	0.0133038
N	14
N Missing	0

Fitted Normal Distribution

Parameter		Estimate	Std Error	Lower 95%	Upper 95%
Location	μ	0.028373	0.0069753	0.0133038	0.0434422
Dispersion	σ	0.0260991	0.0052198	0.0189206	0.0420467

Measures	
-2*LogLikelihood	-63.35366
AICc	-58.26275
BIC	-58.07554

Goodness-of-Fit Test

	W	Prob<W
Shapiro-Wilk	0.8350403	0.0140*

	A^2	Simulated p-Value
Anderson-Darling	0.9516249	0.0108*

Note: Ho = The data is from the Normal distribution. Small p-values reject Ho.

Distributions Herd=low-performing, Indicator=CC-biogenic kgCO2eq

Bedding materials

Compare Distributions

Show	Distribution		AICc ^	BIC	-2*LogLikelihood
☑	Normal		-285.3097	-285.1225	-290.4006

Summary Statistics

Mean	6.7546e-6
Std Dev	7.8525e-6
Std Err Mean	2.0987e-6
Upper 95% Mean	1.1289e-5
Lower 95% Mean	2.2207e-6
N	14
N Missing	0

Fitted Normal Distribution

Parameter		Estimate	Std Error	Lower 95%	Upper 95%
Location	μ	6.7546e-6	2.0987e-6	2.2207e-6	1.1289e-5
Dispersion	σ	7.8525e-6	1.5705e-6	5.6927e-6	1.2651e-5

Measures	
-2*LogLikelihood	-290.4006
AICc	-285.3097
BIC	-285.1225

Goodness-of-Fit Test

	W	Prob<W
Shapiro-Wilk	0.7509725	0.0013*

	A^2	Simulated p-Value
Anderson-Darling	1.3985449	<.0001*

Note: Ho = The data is from the Normal distribution. Small p-values reject Ho.

Distributions Herd=low-performing, Indicator=CC-fossil kgCO2eq

Bedding materials

Compare Distributions

Show	Distribution		AICc ^	BIC	-2*LogLikelihood
☑	Normal		-60.11619	-59.92899	-65.2071

Summary Statistics

Mean	0.0274935
Std Dev	0.0244274
Std Err Mean	0.0065285
Upper 95% Mean	0.0415975
Lower 95% Mean	0.0133896
N	14
N Missing	0

Fitted Normal Distribution

Parameter		Estimate	Std Error	Lower 95%	Upper 95%
Location	μ	0.0274935	0.0065285	0.0133896	0.0415975
Dispersion	σ	0.0244274	0.0048855	0.0177087	0.0393536

Measures	
-2*LogLikelihood	-65.2071
AICc	-60.11619
BIC	-59.92899

Goodness-of-Fit Test

	W	Prob<W
Shapiro-Wilk	0.8460619	0.0196*

	A^2	Simulated p-Value
Anderson-Darling	0.8867012	0.0140*

Note: Ho = The data is from the Normal distribution. Small p-values reject Ho.

Distributions Herd=low-performing, Indicator=CC-LTU kgCO2eq

Bedding materials

Compare Distributions

Show	Distribution		AICc ^	BIC	-2*LogLikelihood
☑	Normal		-127.6803	-127.4931	-132.7712

Summary Statistics

Mean	0.0008727
Std Dev	0.0021874
Std Err Mean	0.0005846
Upper 95% Mean	0.0021356
Lower 95% Mean	-0.00039
N	14
N Missing	0

Fitted Normal Distribution

Parameter		Estimate	Std Error	Lower 95%	Upper 95%
Location	μ	0.0008727	0.0005846	-0.00039	0.0021356
Dispersion	σ	0.0021874	0.0004375	0.0015857	0.003524

Measures	
-2*LogLikelihood	-132.7712
AICc	-127.6803
BIC	-127.4931

Goodness-of-Fit Test

	W	Prob<W
Shapiro-Wilk	0.4660819	<.0001*

	A^2	Simulated p-Value
Anderson-Darling	3.71118	<.0001*

Note: Ho = The data is from the Normal distribution. Small p-values reject Ho.

Distributions Herd=low-performing, Indicator=POCP kgNMVOCeq

Bedding materials

Compare Distributions

Show	Distribution		AICc ^	BIC	-2*LogLikelihood
☑	Normal		-225.0506	-224.8634	-230.1415

Summary Statistics

Mean	7.535e-5
Std Dev	6.7555e-5
Std Err Mean	1.8055e-5
Upper 95% Mean	0.0001144
Lower 95% Mean	3.6345e-5
N	14
N Missing	0

Fitted Normal Distribution

Parameter		Estimate	Std Error	Lower 95%	Upper 95%
Location	μ	7.535e-5	1.8055e-5	3.6345e-5	0.0001144
Dispersion	σ	6.7555e-5	1.3511e-5	4.8974e-5	0.0001088

Measures	
-2*LogLikelihood	-230.1415
AICc	-225.0506
BIC	-224.8634

Goodness-of-Fit Test

	W	Prob<W
Shapiro-Wilk	0.8391411	0.0159*

	A^2	Simulated p-Value
Anderson-Darling	0.9340697	0.0092*

Note: Ho = The data is from the Normal distribution. Small p-values reject Ho.

Distributions Herd=mid-performing, Indicator=CC kgCO2eq

Bedding materials

Compare Distributions

Show	Distribution		AICc ^	BIC	-2*LogLikelihood
☑	Normal		-159.8331	-157.7414	-164.3331

Summary Statistics

Mean	0.0121542
Std Dev	0.0117532
Std Err Mean	0.0022619
Upper 95% Mean	0.0168036
Lower 95% Mean	0.0075048
N	27
N Missing	0

Fitted Normal Distribution

Parameter		Estimate	Std Error	Lower 95%	Upper 95%
Location	μ	0.0121542	0.0022619	0.0075048	0.0168036
Dispersion	σ	0.0117532	0.0016458	0.0092559	0.016107

Measures	
-2*LogLikelihood	-164.3331
AICc	-159.8331
BIC	-157.7414

Goodness-of-Fit Test

	W	Prob<W
Shapiro-Wilk	0.8573885	0.0016*

	A^2	Simulated p-Value
Anderson-Darling	1.031395	0.0104*

Note: Ho = The data is from the Normal distribution. Small p-values reject Ho.

Distributions Herd=mid-performing, Indicator=CC-biogenic kgCO2eq

Bedding materials

Compare Distributions

Show	Distribution		AICc ^	BIC	-2*LogLikelihood
☑	Normal		-620.667	-618.5753	-625.167

Summary Statistics

Mean	2.6421e-6
Std Dev	2.3116e-6
Std Err Mean	4.4486e-7
Upper 95% Mean	3.5565e-6
Lower 95% Mean	1.7277e-6
N	27
N Missing	0

Fitted Normal Distribution

Parameter		Estimate	Std Error	Lower 95%	Upper 95%
Location	μ	2.6421e-6	4.4486e-7	1.7277e-6	3.5565e-6
Dispersion	σ	2.3116e-6	3.2368e-7	1.8204e-6	3.1678e-6

Measures	
-2*LogLikelihood	-625.167
AICc	-620.667
BIC	-618.5753

Goodness-of-Fit Test

	W	Prob<W
Shapiro-Wilk	0.8975498	0.0118*

	A^2	Simulated p-Value
Anderson-Darling	0.703894	0.0576

Note: Ho = The data is from the Normal distribution. Small p-values reject Ho.

Distributions Herd=mid-performing, Indicator=CC-fossil kgCO2eq

Bedding materials

Compare Distributions

Show	Distribution		AICc ^	BIC	-2*LogLikelihood
☑	Normal		-159.8032	-157.7115	-164.3032

Summary Statistics

Mean	0.0121253
Std Dev	0.0117598
Std Err Mean	0.0022632
Upper 95% Mean	0.0167773
Lower 95% Mean	0.0074733
N	27
N Missing	0

Fitted Normal Distribution

Parameter		Estimate	Std Error	Lower 95%	Upper 95%
Location	μ	0.0121253	0.0022632	0.0074733	0.0167773
Dispersion	σ	0.0117598	0.0016467	0.009261	0.0161159

Measures	
-2*LogLikelihood	-164.3032
AICc	-159.8032
BIC	-157.7115

Goodness-of-Fit Test

	W	Prob<W
Shapiro-Wilk	0.856862	0.0016*

	A^2	Simulated p-Value
Anderson-Darling	1.0371833	0.0108*

Note: Ho = The data is from the Normal distribution. Small p-values reject Ho.

Distributions Herd=mid-performing, Indicator=CC-LTU kgCO2eq

Bedding materials

Compare Distributions

Show	Distribution		AICc ^	BIC	-2*LogLikelihood
☑	Normal		-406.6536	-404.5619	-411.1536

Summary Statistics

Mean	2.6285e-5
Std Dev	0.0001216
Std Err Mean	2.3411e-5
Upper 95% Mean	0.0000744
Lower 95% Mean	-2.184e-5
N	27
N Missing	0

Fitted Normal Distribution

Parameter		Estimate	Std Error	Lower 95%	Upper 95%
Location	μ	2.6285e-5	2.3411e-5	-2.184e-5	0.0000744
Dispersion	σ	0.0001216	1.7034e-5	0.0000958	0.0001667

Measures	
-2*LogLikelihood	-411.1536
AICc	-406.6536
BIC	-404.5619

Goodness-of-Fit Test

	W	Prob<W
Shapiro-Wilk	0.2084766	<.0001*

	A^2	Simulated p-Value
Anderson-Darling	9.4681894	<.0001*

Note: Ho = The data is from the Normal distribution. Small p-values reject Ho.

Distributions Herd=mid-performing, Indicator=POCP kgNMVOCeq

Bedding materials

Compare Distributions

Show	Distribution		AICc ^	BIC	-2*LogLikelihood
☑	Normal		-478.2259	-476.1343	-482.7259

Summary Statistics

Mean	3.3817e-5
Std Dev	3.2321e-5
Std Err Mean	6.2202e-6
Upper 95% Mean	0.0000466
Lower 95% Mean	2.1031e-5
N	27
N Missing	0

Fitted Normal Distribution

Parameter		Estimate	Std Error	Lower 95%	Upper 95%
Location	μ	3.3817e-5	6.2202e-6	2.1031e-5	0.0000466
Dispersion	σ	3.2321e-5	4.5259e-6	2.5453e-5	0.0000443

Measures	
-2*LogLikelihood	-482.7259
AICc	-478.2259
BIC	-476.1343

Goodness-of-Fit Test

	W	Prob<W
Shapiro-Wilk	0.8602538	0.0019*

	A^2	Simulated p-Value
Anderson-Darling	0.9992967	0.0108*

Note: Ho = The data is from the Normal distribution. Small p-values reject Ho.
