# Supplementary material for: Climate Change and Photochemical Ozone Creation Potential Impact Indicators of Cow Milk: A Comparison of Different Scenarios for a Diet Assessment
Source: Animals (Basel). 2024 Jun 7;14(12):1725. doi: 10.3390/ani14121725 (PMC11201073; doi:10.3390/ani14121725)
Supplement: Supplementary file 1 [file animals-14-01725-s001.zip › animals-3004812-supplementary/Table 3/Distribution of Energy.pdf]

Distributions Herd=high-performing, Indicator=CC kgCO2eq

Energy

Compare Distributions

Show	Distribution		AICc ^	BIC	-2*LogLikelihood
☑	Normal		-66.44922	-66.26202	-71.54013

Summary Statistics

Mean	0.0581993
Std Dev	0.0194827
Std Err Mean	0.005207
Upper 95% Mean	0.0694483
Lower 95% Mean	0.0469503
N	14
N Missing	0

Fitted Normal Distribution

Parameter		Estimate	Std Error	Lower 95%	Upper 95%
Location	μ	0.0581993	0.005207	0.0469503	0.0694483
Dispersion	σ	0.0194827	0.0038965	0.0141241	0.0313874

Measures	
-2*LogLikelihood	-71.54013
AICc	-66.44922
BIC	-66.26202

Goodness-of-Fit Test

	W	Prob<W
Shapiro-Wilk	0.9837322	0.9909

	A^2	Simulated p-Value
Anderson-Darling	0.1290055	0.9924

Note: Ho = The data is from the Normal distribution. Small p-values reject Ho.

Distributions Herd=high-performing, Indicator=CC-biogenic kgCO2eq

Energy

Compare Distributions

Show	Distribution		AICc ^	BIC	-2*LogLikelihood
☑	Normal		-254.4391	-254.2519	-259.53

Summary Statistics

Mean	7.1438e-5
Std Dev	2.365e-5
Std Err Mean	6.3207e-6
Upper 95% Mean	0.0000851
Lower 95% Mean	5.7783e-5
N	14
N Missing	0

Fitted Normal Distribution

Parameter		Estimate	Std Error	Lower 95%	Upper 95%
Location	μ	7.1438e-5	6.3207e-6	5.7783e-5	0.0000851
Dispersion	σ	2.365e-5	4.73e-6	1.7145e-5	0.0000381

Measures	
-2*LogLikelihood	-259.53
AICc	-254.4391
BIC	-254.2519

Goodness-of-Fit Test

	W	Prob<W
Shapiro-Wilk	0.9290798	0.2962

	A^2	Simulated p-Value
Anderson-Darling	0.4361992	0.2800

Note: Ho = The data is from the Normal distribution. Small p-values reject Ho.

Distributions Herd=high-performing, Indicator=CC-fossil kgCO2eq

Energy

Compare Distributions

Show	Distribution		AICc ^	BIC	-2*LogLikelihood
☑	Normal		-66.59468	-66.40747	-71.68559

Summary Statistics

Mean	0.0578514
Std Dev	0.0193817
Std Err Mean	0.00518
Upper 95% Mean	0.0690421
Lower 95% Mean	0.0466607
N	14
N Missing	0

Fitted Normal Distribution

Parameter		Estimate	Std Error	Lower 95%	Upper 95%
Location	μ	0.0578514	0.00518	0.0466607	0.0690421
Dispersion	σ	0.0193817	0.0038763	0.0140509	0.0312248

Measures	
-2*LogLikelihood	-71.68559
AICc	-66.59468
BIC	-66.40747

Goodness-of-Fit Test

	W	Prob<W
Shapiro-Wilk	0.9840368	0.9918

	A^2	Simulated p-Value
Anderson-Darling	0.1275518	0.9908

Note: Ho = The data is from the Normal distribution. Small p-values reject Ho.

Distributions Herd=high-performing, Indicator=CC-LTU kgCO2eq

Energy

Compare Distributions

Show	Distribution		AICc ^	BIC	-2*LogLikelihood
☑	Normal		-215.9888	-215.8016	-221.0797

Summary Statistics

Mean	0.0002764
Std Dev	9.3371e-5
Std Err Mean	2.4954e-5
Upper 95% Mean	0.0003304
Lower 95% Mean	0.0002225
N	14
N Missing	0

Fitted Normal Distribution

Parameter		Estimate	Std Error	Lower 95%	Upper 95%
Location	μ	0.0002764	2.4954e-5	0.0002225	0.0003304
Dispersion	σ	9.3371e-5	1.8674e-5	6.769e-5	0.0001504

Measures	
-2*LogLikelihood	-221.0797
AICc	-215.9888
BIC	-215.8016

Goodness-of-Fit Test

	W	Prob<W
Shapiro-Wilk	0.9217728	0.2332

	A^2	Simulated p-Value
Anderson-Darling	0.4895048	0.1952

Note: Ho = The data is from the Normal distribution. Small p-values reject Ho.

Distributions Herd=high-performing, Indicator=POCP kgNMVOCeq

Energy

Compare Distributions

Show	Distribution		AICc ^	BIC	-2*LogLikelihood
☑	Normal		-192.0376	-191.8504	-197.1285

Summary Statistics

Mean	0.0006584
Std Dev	0.0002196
Std Err Mean	0.0000587
Upper 95% Mean	0.0007852
Lower 95% Mean	0.0005315
N	14
N Missing	0

Fitted Normal Distribution

Parameter		Estimate	Std Error	Lower 95%	Upper 95%
Location	μ	0.0006584	0.0000587	0.0005315	0.0007852
Dispersion	σ	0.0002196	4.3928e-5	0.0001592	0.0003538

Measures	
-2*LogLikelihood	-197.1285
AICc	-192.0376
BIC	-191.8504

Goodness-of-Fit Test

	W	Prob<W
Shapiro-Wilk	0.92526	0.2615

	A^2	Simulated p-Value
Anderson-Darling	0.4654033	0.2204

Note: Ho = The data is from the Normal distribution. Small p-values reject Ho.

Distributions Herd=low-performing, Indicator=CC kgCO2eq

Energy

Compare Distributions

Show	Distribution		AICc ^	BIC	-2*LogLikelihood
☑	Normal		-54.00769	-53.82048	-59.09859

Summary Statistics

Mean	0.1159467
Std Dev	0.0303825
Std Err Mean	0.0081201
Upper 95% Mean	0.1334891
Lower 95% Mean	0.0984044
N	14
N Missing	0

Fitted Normal Distribution

Parameter		Estimate	Std Error	Lower 95%	Upper 95%
Location	μ	0.1159467	0.0081201	0.0984044	0.1334891
Dispersion	σ	0.0303825	0.0060765	0.0220259	0.0489475

Measures	
-2*LogLikelihood	-59.09859
AICc	-54.00769
BIC	-53.82048

Goodness-of-Fit Test

	W	Prob<W
Shapiro-Wilk	0.9417745	0.4415

	A^2	Simulated p-Value
Anderson-Darling	0.3272089	0.5028

Note: Ho = The data is from the Normal distribution. Small p-values reject Ho.

Distributions Herd=low-performing, Indicator=CC-biogenic kgCO2eq

Energy

Compare Distributions

Show	Distribution		AICc ^	BIC	-2*LogLikelihood
☑	Normal		-234.8346	-234.6474	-239.9256

Summary Statistics

Mean	0.0001618
Std Dev	4.7633e-5
Std Err Mean	1.273e-5
Upper 95% Mean	0.0001893
Lower 95% Mean	0.0001343
N	14
N Missing	0

Fitted Normal Distribution

Parameter		Estimate	Std Error	Lower 95%	Upper 95%
Location	μ	0.0001618	1.273e-5	0.0001343	0.0001893
Dispersion	σ	4.7633e-5	9.5265e-6	3.4531e-5	7.6738e-5

Measures	
-2*LogLikelihood	-239.9256
AICc	-234.8346
BIC	-234.6474

Goodness-of-Fit Test

	W	Prob<W
Shapiro-Wilk	0.938556	0.4001

	A^2	Simulated p-Value
Anderson-Darling	0.3066662	0.5512

Note: Ho = The data is from the Normal distribution. Small p-values reject Ho.

Distributions Herd=low-performing, Indicator=CC-fossil kgCO2eq

Energy

Compare Distributions

Show	Distribution		AICc ^	BIC	-2*LogLikelihood
☑	Normal		-54.21937	-54.03217	-59.31028

Summary Statistics

Mean	0.1151448
Std Dev	0.0301537
Std Err Mean	0.0080589
Upper 95% Mean	0.132555
Lower 95% Mean	0.0977346
N	14
N Missing	0

Fitted Normal Distribution

Parameter		Estimate	Std Error	Lower 95%	Upper 95%
Location	μ	0.1151448	0.0080589	0.0977346	0.132555
Dispersion	σ	0.0301537	0.0060307	0.02186	0.0485788

Measures	
-2*LogLikelihood	-59.31028
AICc	-54.21937
BIC	-54.03217

Goodness-of-Fit Test

	W	Prob<W
Shapiro-Wilk	0.9415909	0.4391

	A^2	Simulated p-Value
Anderson-Darling	0.3294871	0.5000

Note: Ho = The data is from the Normal distribution. Small p-values reject Ho.

Distributions Herd=low-performing, Indicator=CC-LTU kgCO2eq

Energy

Compare Distributions

Show	Distribution		AICc ^	BIC	-2*LogLikelihood
☑	Normal		-195.6814	-195.4942	-200.7723

Summary Statistics

Mean	0.0006402
Std Dev	0.0001928
Std Err Mean	5.1538e-5
Upper 95% Mean	0.0007515
Lower 95% Mean	0.0005288
N	14
N Missing	0

Fitted Normal Distribution

Parameter		Estimate	Std Error	Lower 95%	Upper 95%
Location	μ	0.0006402	5.1538e-5	0.0005288	0.0007515
Dispersion	σ	0.0001928	3.8567e-5	0.0001398	0.0003107

Measures	
-2*LogLikelihood	-200.7723
AICc	-195.6814
BIC	-195.4942

Goodness-of-Fit Test

	W	Prob<W
Shapiro-Wilk	0.9394101	0.4108

	A^2	Simulated p-Value
Anderson-Darling	0.3090067	0.5444

Note: Ho = The data is from the Normal distribution. Small p-values reject Ho.

Distributions Herd=low-performing, Indicator=POCP kgNMVOCeq

Energy

Compare Distributions

Show	Distribution		AICc ^	BIC	-2*LogLikelihood
☑	Normal		-172.0698	-171.8826	-177.1607

Summary Statistics

Mean	0.0015071
Std Dev	0.0004481
Std Err Mean	0.0001198
Upper 95% Mean	0.0017658
Lower 95% Mean	0.0012483
N	14
N Missing	0

Fitted Normal Distribution

Parameter		Estimate	Std Error	Lower 95%	Upper 95%
Location	μ	0.0015071	0.0001198	0.0012483	0.0017658
Dispersion	σ	0.0004481	8.9629e-5	0.0003249	0.000722

Measures	
-2*LogLikelihood	-177.1607
AICc	-172.0698
BIC	-171.8826

Goodness-of-Fit Test

	W	Prob<W
Shapiro-Wilk	0.93772	0.3899

	A^2	Simulated p-Value
Anderson-Darling	0.309301	0.5480

Note: Ho = The data is from the Normal distribution. Small p-values reject Ho.

Distributions Herd=mid-performing, Indicator=CC kgCO2eq

Energy

Compare Distributions

Show	Distribution		AICc ^	BIC	-2*LogLikelihood
☑	Normal		-116.386	-114.2943	-120.886

Summary Statistics

Mean	0.0796123
Std Dev	0.0262773
Std Err Mean	0.0050571
Upper 95% Mean	0.0900073
Lower 95% Mean	0.0692174
N	27
N Missing	0

Fitted Normal Distribution

Parameter		Estimate	Std Error	Lower 95%	Upper 95%
Location	μ	0.0796123	0.0050571	0.0692174	0.0900073
Dispersion	σ	0.0262773	0.0036796	0.0206938	0.0360113

Measures	
-2*LogLikelihood	-120.886
AICc	-116.386
BIC	-114.2943

Goodness-of-Fit Test

	W	Prob<W
Shapiro-Wilk	0.826196	0.0004*

	A^2	Simulated p-Value
Anderson-Darling	1.2347045	0.0016*

Note: Ho = The data is from the Normal distribution. Small p-values reject Ho.

Distributions Herd=mid-performing, Indicator=CC-biogenic kgCO2eq

Energy

Compare Distributions

Show	Distribution		AICc ^	BIC	-2*LogLikelihood
☑	Normal		-459.3015	-457.2098	-463.8015

Summary Statistics

Mean	0.0001033
Std Dev	4.5887e-5
Std Err Mean	8.8309e-6
Upper 95% Mean	0.0001215
Lower 95% Mean	8.5177e-5
N	27
N Missing	0

Fitted Normal Distribution

Parameter		Estimate	Std Error	Lower 95%	Upper 95%
Location	μ	0.0001033	8.8309e-6	8.5177e-5	0.0001215
Dispersion	σ	4.5887e-5	6.4254e-6	3.6137e-5	6.2885e-5

Measures	
-2*LogLikelihood	-463.8015
AICc	-459.3015
BIC	-457.2098

Goodness-of-Fit Test

	W	Prob<W
Shapiro-Wilk	0.7945641	0.0001*

	A^2	Simulated p-Value
Anderson-Darling	1.3809282	0.0008*

Note: Ho = The data is from the Normal distribution. Small p-values reject Ho.

Distributions Herd=mid-performing, Indicator=CC-fossil kgCO2eq

Energy

Compare Distributions

Show	Distribution		AICc ^	BIC	-2*LogLikelihood
☑	Normal		-116.8542	-114.7625	-121.3542

Summary Statistics

Mean	0.079105
Std Dev	0.0260505
Std Err Mean	0.0050134
Upper 95% Mean	0.0894103
Lower 95% Mean	0.0687998
N	27
N Missing	0

Fitted Normal Distribution

Parameter		Estimate	Std Error	Lower 95%	Upper 95%
Location	μ	0.079105	0.0050134	0.0687998	0.0894103
Dispersion	σ	0.0260505	0.0036478	0.0205152	0.0357004

Measures	
-2*LogLikelihood	-121.3542
AICc	-116.8542
BIC	-114.7625

Goodness-of-Fit Test

	W	Prob<W
Shapiro-Wilk	0.826662	0.0004*

	A^2	Simulated p-Value
Anderson-Darling	1.2321079	0.0032*

Note: Ho = The data is from the Normal distribution. Small p-values reject Ho.

Distributions Herd=mid-performing, Indicator=CC-LTU kgCO2eq

Energy

Compare Distributions

Show	Distribution		AICc ^	BIC	-2*LogLikelihood
☑	Normal		-383.1844	-381.0928	-387.6844

Summary Statistics

Mean	0.0004039
Std Dev	0.0001879
Std Err Mean	3.6156e-5
Upper 95% Mean	0.0004783
Lower 95% Mean	0.0003296
N	27
N Missing	0

Fitted Normal Distribution

Parameter		Estimate	Std Error	Lower 95%	Upper 95%
Location	μ	0.0004039	3.6156e-5	0.0003296	0.0004783
Dispersion	σ	0.0001879	0.0000263	0.000148	0.0002575

Measures	
-2*LogLikelihood	-387.6844
AICc	-383.1844
BIC	-381.0928

Goodness-of-Fit Test

	W	Prob<W
Shapiro-Wilk	0.7939033	0.0001*

	A^2	Simulated p-Value
Anderson-Darling	1.3859117	0.0012*

Note: Ho = The data is from the Normal distribution. Small p-values reject Ho.

Distributions Herd=mid-performing, Indicator=POCP kgNMVOCeq

Energy

Compare Distributions

Show	Distribution		AICc ^	BIC	-2*LogLikelihood
☑	Normal		-337.9583	-335.8666	-342.4583

Summary Statistics

Mean	0.0009572
Std Dev	0.0004341
Std Err Mean	8.3542e-5
Upper 95% Mean	0.0011289
Lower 95% Mean	0.0007854
N	27
N Missing	0

Fitted Normal Distribution

Parameter		Estimate	Std Error	Lower 95%	Upper 95%
Location	μ	0.0009572	8.3542e-5	0.0007854	0.0011289
Dispersion	σ	0.0004341	6.0786e-5	0.0003419	0.0005949

Measures	
-2*LogLikelihood	-342.4583
AICc	-337.9583
BIC	-335.8666

Goodness-of-Fit Test

	W	Prob<W
Shapiro-Wilk	0.7943774	0.0001*

	A^2	Simulated p-Value
Anderson-Darling	1.3812607	0.0008*

Note: Ho = The data is from the Normal distribution. Small p-values reject Ho.
