# Supplementary material for: Climate Change and Photochemical Ozone Creation Potential Impact Indicators of Cow Milk: A Comparison of Different Scenarios for a Diet Assessment
Source: Animals (Basel). 2024 Jun 7;14(12):1725. doi: 10.3390/ani14121725 (PMC11201073; doi:10.3390/ani14121725)
Supplement: Supplementary file 1 [file animals-14-01725-s001.zip › animals-3004812-supplementary/Table 3/Distribution of Enteric fermentation.pdf]

Distributions Herd=high-performing, Indicator=CC kgCO2eq

Enteric fermentation

Compare Distributions

Show	Distribution		AICc ^	BIC	-2*LogLikelihood
☑	Normal	—	-33.26302	-33.07582	-38.35393

Summary Statistics

Mean	0.6295688
Std Dev	0.0637359
Std Err Mean	0.0170341
Upper 95% Mean	0.6663688
Lower 95% Mean	0.5927688
N	14
N Missing	0

Fitted Normal Distribution

Parameter		Estimate	Std Error	Lower 95%	Upper 95%
Location	μ	0.6295688	0.0170341	0.5927688	0.6663688
Dispersion	σ	0.0637359	0.0127472	0.0462056	0.1026812

Measures	
-2*LogLikelihood	-38.35393
AICc	-33.26302
BIC	-33.07582

Goodness-of-Fit Test

	W	Prob<W
Shapiro-Wilk	0.9609226	0.7383

	A²	Simulated p-Value
Anderson-Darling	0.2217817	0.8088

Note: Ho = The data is from the Normal distribution. Small p-values reject Ho.

Distributions Herd=high-performing, Indicator=CC-biogenic kgCO2eq

Enteric fermentation

Compare Distributions

Show	Distribution		AICc ^	BIC	-2*LogLikelihood
☑	Normal	—	-33.26302	-33.07582	-38.35393

Summary Statistics

Mean	0.6295688
Std Dev	0.0637359
Std Err Mean	0.0170341
Upper 95% Mean	0.6663688
Lower 95% Mean	0.5927688
N	14
N Missing	0

Fitted Normal Distribution

Parameter		Estimate	Std Error	Lower 95%	Upper 95%
Location	μ	0.6295688	0.0170341	0.5927688	0.6663688
Dispersion	σ	0.0637359	0.0127472	0.0462056	0.1026812

Measures	
-2*LogLikelihood	-38.35393
AICc	-33.26302
BIC	-33.07582

Goodness-of-Fit Test

	W	Prob<W
Shapiro-Wilk	0.9609226	0.7383

	A²	Simulated p-Value
Anderson-Darling	0.2217817	0.8224

Note: Ho = The data is from the Normal distribution. Small p-values reject Ho.

Distributions Herd=high-performing, Indicator=CC-fossil kgCO2eq

Enteric fermentation

Summary Statistics

Mean	0
Std Dev	0
Std Err Mean	0
Upper 95% Mean	0
Lower 95% Mean	0
N	14
N Missing	0

Distributions Herd=high-performing, Indicator=CC-LTU kgCO2eq

Enteric fermentation

Summary Statistics

Mean	0
Std Dev	0
Std Err Mean	0
Upper 95% Mean	0
Lower 95% Mean	0
N	14
N Missing	0

Distributions Herd=high-performing, Indicator=POCP kgNMVOCeq

Enteric fermentation

Compare Distributions

Show	Distribution		AICc ^	BIC	-2*LogLikelihood
☑	Normal	—	-260.6673	-260.4801	-265.7582

Summary Statistics

Mean	0.000187
Std Dev	1.8933e-5
Std Err Mean	5.0601e-6
Upper 95% Mean	0.000198
Lower 95% Mean	0.0001761
N	14
N Missing	0

Fitted Normal Distribution

Parameter		Estimate	Std Error	Lower 95%	Upper 95%
Location	μ	0.000187	5.0601e-6	0.0001761	0.000198
Dispersion	σ	1.8933e-5	3.7867e-6	1.3726e-5	0.0000305

Measures	
-2*LogLikelihood	-265.7582
AICc	-260.6673
BIC	-260.4801

Goodness-of-Fit Test

	W	Prob<W
Shapiro-Wilk	0.9609226	0.7383

	A²	Simulated p-Value
Anderson-Darling	0.2217816	0.8128

Note: Ho = The data is from the Normal distribution. Small p-values reject Ho.

Distributions Herd=low-performing, Indicator=CC kgCO2eq

Enteric fermentation

Compare Distributions

Show	Distribution		AICc ^	BIC	-2*LogLikelihood
☑	Normal	—	-21.73527	-21.54806	-26.82618

Summary Statistics

Mean	0.8137464
Std Dev	0.0962023
Std Err Mean	0.0257111
Upper 95% Mean	0.869292
Lower 95% Mean	0.7582009
N	14
N Missing	0

Fitted Normal Distribution

Parameter		Estimate	Std Error	Lower 95%	Upper 95%
Location	μ	0.8137464	0.0257111	0.7582009	0.869292
Dispersion	σ	0.0962023	0.0192405	0.0697422	0.154986

Measures	
-2*LogLikelihood	-26.82618
AICc	-21.73527
BIC	-21.54806

Goodness-of-Fit Test

	W	Prob<W
Shapiro-Wilk	0.9586009	0.7000

	A²	Simulated p-Value
Anderson-Darling	0.3365424	0.4652

Note: Ho = The data is from the Normal distribution. Small p-values reject Ho.

Distributions Herd=low-performing, Indicator=CC-biogenic kgCO2eq

Enteric fermentation

Compare Distributions

Show	Distribution		AICc ^	BIC	-2*LogLikelihood
☑	Normal	—	-21.73527	-21.54806	-26.82618

Summary Statistics

Mean	0.8137464
Std Dev	0.0962023
Std Err Mean	0.0257111
Upper 95% Mean	0.869292
Lower 95% Mean	0.7582009
N	14
N Missing	0

Fitted Normal Distribution

Parameter		Estimate	Std Error	Lower 95%	Upper 95%
Location	μ	0.8137464	0.0257111	0.7582009	0.869292
Dispersion	σ	0.0962023	0.0192405	0.0697422	0.154986

Measures	
-2*LogLikelihood	-26.82618
AICc	-21.73527
BIC	-21.54806

Goodness-of-Fit Test

	W	Prob<W
Shapiro-Wilk	0.9586009	0.7000

	A²	Simulated p-Value
Anderson-Darling	0.3365424	0.4800

Note: Ho = The data is from the Normal distribution. Small p-values reject Ho.

Distributions Herd=low-performing, Indicator=CC-fossil kgCO2eq

Enteric fermentation

Summary Statistics

Mean	0
Std Dev	0
Std Err Mean	0
Upper 95% Mean	0
Lower 95% Mean	0
N	14
N Missing	0

Distributions Herd=low-performing, Indicator=CC-LTU kgCO2eq

Enteric fermentation

Summary Statistics

Mean	0
Std Dev	0
Std Err Mean	0
Upper 95% Mean	0
Lower 95% Mean	0
N	14
N Missing	0

Distributions Herd=low-performing, Indicator=POCP kgNMVOCeq

Enteric fermentation

Compare Distributions

Show	Distribution		AICc ^	BIC	-2*LogLikelihood
☑	Normal	—	-249.1395	-248.9523	-254.2304

Summary Statistics

Mean	0.0002417
Std Dev	2.8578e-5
Std Err Mean	7.6377e-6
Upper 95% Mean	0.0002582
Lower 95% Mean	0.0002252
N	14
N Missing	0

Fitted Normal Distribution

Parameter		Estimate	Std Error	Lower 95%	Upper 95%
Location	μ	0.0002417	7.6377e-6	0.0002252	0.0002582
Dispersion	σ	2.8578e-5	5.7155e-6	2.0718e-5	4.604e-5

Measures	
-2*LogLikelihood	-254.2304
AICc	-249.1395
BIC	-248.9523

Goodness-of-Fit Test

	W	Prob<W
Shapiro-Wilk	0.9586009	0.7000

	A²	Simulated p-Value
Anderson-Darling	0.3365425	0.4620

Note: Ho = The data is from the Normal distribution. Small p-values reject Ho.

Distributions Herd=mid-performing, Indicator=CC kgCO2eq

Enteric fermentation

Compare Distributions

Show	Distribution		AICc ^	BIC	-2*LogLikelihood
☑	Normal	—	-89.44917	-87.3575	-93.94917

Summary Statistics

Mean	0.7109376
Std Dev	0.0432733
Std Err Mean	0.008328
Upper 95% Mean	0.7280559
Lower 95% Mean	0.6938192
N	27
N Missing	0

Fitted Normal Distribution

Parameter		Estimate	Std Error	Lower 95%	Upper 95%
Location	μ	0.7109376	0.008328	0.6938192	0.7280559
Dispersion	σ	0.0432733	0.0060595	0.0340784	0.0593031

Measures	
-2*LogLikelihood	-93.94917
AICc	-89.44917
BIC	-87.3575

Goodness-of-Fit Test

	W	Prob<W
Shapiro-Wilk	0.9340942	0.0871

	A²	Simulated p-Value
Anderson-Darling	0.6807313	0.0632

Note: Ho = The data is from the Normal distribution. Small p-values reject Ho.

Distributions Herd=mid-performing, Indicator=CC-biogenic kgCO2eq

Enteric fermentation

Compare Distributions

Show	Distribution		AICc ^	BIC	-2*LogLikelihood
☑	Normal	—	-89.44917	-87.3575	-93.94917

Summary Statistics

Mean	0.7109376
Std Dev	0.0432733
Std Err Mean	0.008328
Upper 95% Mean	0.7280559
Lower 95% Mean	0.6938192
N	27
N Missing	0

Fitted Normal Distribution

Parameter		Estimate	Std Error	Lower 95%	Upper 95%
Location	μ	0.7109376	0.008328	0.6938192	0.7280559
Dispersion	σ	0.0432733	0.0060595	0.0340784	0.0593031

Measures	
-2*LogLikelihood	-93.94917
AICc	-89.44917
BIC	-87.3575

Goodness-of-Fit Test

	W	Prob<W
Shapiro-Wilk	0.9340942	0.0871

	A²	Simulated p-Value
Anderson-Darling	0.6807313	0.0688

Note: Ho = The data is from the Normal distribution. Small p-values reject Ho.

Distributions Herd=mid-performing, Indicator=CC-fossil kgCO2eq

Enteric fermentation

Summary Statistics

Mean	0
Std Dev	0
Std Err Mean	0
Upper 95% Mean	0
Lower 95% Mean	0
N	27
N Missing	0

Distributions Herd=mid-performing, Indicator=CC-LTU kgCO2eq

Enteric fermentation

Summary Statistics

Mean	0
Std Dev	0
Std Err Mean	0
Upper 95% Mean	0
Lower 95% Mean	0
N	27
N Missing	0

Distributions Herd=mid-performing, Indicator=POCP kgNMVOCeq

Enteric fermentation

Compare Distributions

Show	Distribution		AICc ^	BIC	-2*LogLikelihood
☑	Normal	—	-528.0145	-525.9228	-532.5145

Summary Statistics

Mean	0.0002112
Std Dev	1.2855e-5
Std Err Mean	2.4739e-6
Upper 95% Mean	0.0002163
Lower 95% Mean	0.0002061
N	27
N Missing	0

Fitted Normal Distribution

Parameter		Estimate	Std Error	Lower 95%	Upper 95%
Location	μ	0.0002112	2.4739e-6	0.0002061	0.0002163
Dispersion	σ	1.2855e-5	0.0000018	1.0123e-5	1.7617e-5

Measures	
-2*LogLikelihood	-532.5145
AICc	-528.0145
BIC	-525.9228

Goodness-of-Fit Test

	W	Prob<W
Shapiro-Wilk	0.9340942	0.0871

	A²	Simulated p-Value
Anderson-Darling	0.6807311	0.0608

Note: Ho = The data is from the Normal distribution. Small p-values reject Ho.
