# Supplementary material for: Climate Change and Photochemical Ozone Creation Potential Impact Indicators of Cow Milk: A Comparison of Different Scenarios for a Diet Assessment
Source: Animals (Basel). 2024 Jun 7;14(12):1725. doi: 10.3390/ani14121725 (PMC11201073; doi:10.3390/ani14121725)
Supplement: Supplementary file 1 [file animals-14-01725-s001.zip › animals-3004812-supplementary/Table 3/Distribution of Feeds purchase.pdf]

Distributions Herd=high-performing, Indicator=CC kgCO2eq

Feeds purchase

Compare Distributions

Show	Distribution		AICc ^	BIC	-2*LogLikelihood
☑	Normal		-8.960773	-8.773568	-14.05168

Summary Statistics

Mean	0.4969291
Std Dev	0.1518184
Std Err Mean	0.0405752
Upper 95% Mean	0.5845864
Lower 95% Mean	0.4092717
N	14
N Missing	0

Fitted Normal Distribution

Parameter		Estimate	Std Error	Lower 95%	Upper 95%
Location	μ	0.4969291	0.0405752	0.4092717	0.5845864
Dispersion	σ	0.1518184	0.0303637	0.1100614	0.2445859

Measures	
-2*LogLikelihood	-14.05168
AICc	-8.960773
BIC	-8.773568

Goodness-of-Fit Test

	W	Prob<W
Shapiro-Wilk	0.9603005	0.7280

	A²	Simulated p-Value
Anderson-Darling	0.2328415	0.7760

Note: Ho = The data is from the Normal distribution. Small p-values reject Ho.

Distributions Herd=high-performing, Indicator=CC-biogenic kgCO2eq

Feeds purchase

Compare Distributions

Show	Distribution		AICc ^	BIC	-2*LogLikelihood
☑	Normal		-97.58626	-97.39905	-102.6772

Summary Statistics

Mean	0.0096311
Std Dev	0.0064076
Std Err Mean	0.0017125
Upper 95% Mean	0.0133308
Lower 95% Mean	0.0059315
N	14
N Missing	0

Fitted Normal Distribution

Parameter		Estimate	Std Error	Lower 95%	Upper 95%
Location	μ	0.0096311	0.0017125	0.0059315	0.0133308
Dispersion	σ	0.0064076	0.0012815	0.0046452	0.010323

Measures	
-2*LogLikelihood	-102.6772
AICc	-97.58626
BIC	-97.39905

Goodness-of-Fit Test

	W	Prob<W
Shapiro-Wilk	0.9330036	0.3360

	A²	Simulated p-Value
Anderson-Darling	0.371371	0.3940

Note: Ho = The data is from the Normal distribution. Small p-values reject Ho.

Distributions Herd=high-performing, Indicator=CC-fossil kgCO2eq

Feeds purchase

Compare Distributions

Show	Distribution		AICc ^	BIC	-2*LogLikelihood
☑	Normal		-20.86025	-20.67304	-25.95115

Summary Statistics

Mean	0.2482695
Std Dev	0.0992562
Std Err Mean	0.0265273
Upper 95% Mean	0.3055783
Lower 95% Mean	0.1909607
N	14
N Missing	0

Fitted Normal Distribution

Parameter		Estimate	Std Error	Lower 95%	Upper 95%
Location	μ	0.2482695	0.0265273	0.1909607	0.3055783
Dispersion	σ	0.0992562	0.0198512	0.0719561	0.1599059

Measures	
-2*LogLikelihood	-25.95115
AICc	-20.86025
BIC	-20.67304

Goodness-of-Fit Test

	W	Prob<W
Shapiro-Wilk	0.9617481	0.7518

	A²	Simulated p-Value
Anderson-Darling	0.3022145	0.5568

Note: Ho = The data is from the Normal distribution. Small p-values reject Ho.

Distributions Herd=high-performing, Indicator=CC-LTU kgCO2eq

Feeds purchase

Compare Distributions

Show	Distribution		AICc ^	BIC	-2*LogLikelihood
☑	Normal		-20.58982	-20.40261	-25.68073

Summary Statistics

Mean	0.2390284
Std Dev	0.1002194
Std Err Mean	0.0267848
Upper 95% Mean	0.2968934
Lower 95% Mean	0.1811634
N	14
N Missing	0

Fitted Normal Distribution

Parameter		Estimate	Std Error	Lower 95%	Upper 95%
Location	μ	0.2390284	0.0267848	0.1811634	0.2968934
Dispersion	σ	0.1002194	0.0200439	0.0726545	0.1614578

Measures	
-2*LogLikelihood	-25.68073
AICc	-20.58982
BIC	-20.40261

Goodness-of-Fit Test

	W	Prob<W
Shapiro-Wilk	0.8675595	0.0388*

	A²	Simulated p-Value
Anderson-Darling	0.6081038	0.0936

Note: Ho = The data is from the Normal distribution. Small p-values reject Ho.

Distributions Herd=high-performing, Indicator=POCP kgNMVOCeq

Feeds purchase

Compare Distributions

Show	Distribution		AICc ^	BIC	-2*LogLikelihood
☑	Normal		-186.8045	-186.6173	-191.8955

Summary Statistics

Mean	0.0007271
Std Dev	0.0002648
Std Err Mean	7.0764e-5
Upper 95% Mean	0.00088
Lower 95% Mean	0.0005742
N	14
N Missing	0

Fitted Normal Distribution

Parameter		Estimate	Std Error	Lower 95%	Upper 95%
Location	μ	0.0007271	7.0764e-5	0.0005742	0.00088
Dispersion	σ	0.0002648	5.2955e-5	0.0001919	0.0004266

Measures	
-2*LogLikelihood	-191.8955
AICc	-186.8045
BIC	-186.6173

Goodness-of-Fit Test

	W	Prob<W
Shapiro-Wilk	0.9268659	0.2756

	A²	Simulated p-Value
Anderson-Darling	0.4244689	0.2668

Note: Ho = The data is from the Normal distribution. Small p-values reject Ho.

Distributions Herd=low-performing, Indicator=CC kgCO2eq

Feeds purchase

Compare Distributions

Show	Distribution		AICc ^	BIC	-2*LogLikelihood
☑	Normal		1.9905616	2.1777672	-3.100348

Summary Statistics

Mean	0.6397994
Std Dev	0.224484
Std Err Mean	0.0599959
Upper 95% Mean	0.7694126
Lower 95% Mean	0.5101862
N	14
N Missing	0

Fitted Normal Distribution

Parameter		Estimate	Std Error	Lower 95%	Upper 95%
Location	μ	0.6397994	0.0599959	0.5101862	0.7694126
Dispersion	σ	0.224484	0.0448968	0.1627405	0.3616532

Measures	
-2*LogLikelihood	-3.100348
AICc	1.9905616
BIC	2.1777672

Goodness-of-Fit Test

	W	Prob<W
Shapiro-Wilk	0.9275854	0.2822

	A²	Simulated p-Value
Anderson-Darling	0.3710074	0.3760

Note: Ho = The data is from the Normal distribution. Small p-values reject Ho.

Distributions Herd=low-performing, Indicator=CC-biogenic kgCO2eq

Feeds purchase

Compare Distributions

Show	Distribution		AICc ^	BIC	-2*LogLikelihood
☑	Normal		-48.32258	-48.13538	-53.41349

Summary Statistics

Mean	0.0234209
Std Dev	0.0372222
Std Err Mean	0.0099481
Upper 95% Mean	0.0449124
Lower 95% Mean	0.0019294
N	14
N Missing	0

Fitted Normal Distribution

Parameter		Estimate	Std Error	Lower 95%	Upper 95%
Location	μ	0.0234209	0.0099481	0.0019294	0.0449124
Dispersion	σ	0.0372222	0.0074444	0.0269844	0.0599666

Measures	
-2*LogLikelihood	-53.41349
AICc	-48.32258
BIC	-48.13538

Goodness-of-Fit Test

	W	Prob<W
Shapiro-Wilk	0.6172154	<.0001*

	A²	Simulated p-Value
Anderson-Darling	1.9694622	<.0001*

Note: Ho = The data is from the Normal distribution. Small p-values reject Ho.

Distributions Herd=low-performing, Indicator=CC-fossil kgCO2eq

Feeds purchase

Compare Distributions

Show	Distribution		AICc ^	BIC	-2*LogLikelihood
☑	Normal		-16.91992	-16.73272	-22.01083

Summary Statistics

Mean	0.2521952
Std Dev	0.1142547
Std Err Mean	0.0305358
Upper 95% Mean	0.3181639
Lower 95% Mean	0.1862265
N	14
N Missing	0

Fitted Normal Distribution

Parameter		Estimate	Std Error	Lower 95%	Upper 95%
Location	μ	0.2521952	0.0305358	0.1862265	0.3181639
Dispersion	σ	0.1142547	0.0228509	0.0828294	0.1840691

Measures	
-2*LogLikelihood	-22.01083
AICc	-16.91992
BIC	-16.73272

Goodness-of-Fit Test

	W	Prob<W
Shapiro-Wilk	0.9203897	0.2228

	A²	Simulated p-Value
Anderson-Darling	0.4311617	0.2716

Note: Ho = The data is from the Normal distribution. Small p-values reject Ho.

Distributions Herd=low-performing, Indicator=CC-LTU kgCO2eq

Feeds purchase

Compare Distributions

Show	Distribution		AICc ^	BIC	-2*LogLikelihood
☑	Normal		-13.25231	-13.0651	-18.34322

Summary Statistics

Mean	0.3641833
Std Dev	0.1302448
Std Err Mean	0.0348094
Upper 95% Mean	0.4393845
Lower 95% Mean	0.2889822
N	14
N Missing	0

Fitted Normal Distribution

Parameter		Estimate	Std Error	Lower 95%	Upper 95%
Location	μ	0.3641833	0.0348094	0.2889822	0.4393845
Dispersion	σ	0.1302448	0.026049	0.0944215	0.20983

Measures	
-2*LogLikelihood	-18.34322
AICc	-13.25231
BIC	-13.0651

Goodness-of-Fit Test

	W	Prob<W
Shapiro-Wilk	0.9188884	0.2119

	A²	Simulated p-Value
Anderson-Darling	0.4171941	0.3000

Note: Ho = The data is from the Normal distribution. Small p-values reject Ho.

Distributions Herd=low-performing, Indicator=POCP kgNMVOCeq

Feeds purchase

Compare Distributions

Show	Distribution		AICc ^	BIC	-2*LogLikelihood
☑	Normal		-185.339	-185.1518	-190.4299

Summary Statistics

Mean	0.0007626
Std Dev	0.000279
Std Err Mean	7.4566e-5
Upper 95% Mean	0.0009237
Lower 95% Mean	0.0006015
N	14
N Missing	0

Fitted Normal Distribution

Parameter		Estimate	Std Error	Lower 95%	Upper 95%
Location	μ	0.0007626	7.4566e-5	0.0006015	0.0009237
Dispersion	σ	0.000279	0.0000558	0.0002023	0.0004495

Measures	
-2*LogLikelihood	-190.4299
AICc	-185.339
BIC	-185.1518

Goodness-of-Fit Test

	W	Prob<W
Shapiro-Wilk	0.9142148	0.1814

	A²	Simulated p-Value
Anderson-Darling	0.4651244	0.2204

Note: Ho = The data is from the Normal distribution. Small p-values reject Ho.

Distributions Herd=mid-performing, Indicator=CC kgCO2eq

Feeds purchase

Compare Distributions

Show	Distribution		AICc ^	BIC	-2*LogLikelihood
☑	Normal		-10.97643	-8.884759	-15.47643

Summary Statistics

Mean	0.5920495
Std Dev	0.1850701
Std Err Mean	0.0356168
Upper 95% Mean	0.6652608
Lower 95% Mean	0.5188382
N	27
N Missing	0

Fitted Normal Distribution

Parameter		Estimate	Std Error	Lower 95%	Upper 95%
Location	μ	0.5920495	0.0356168	0.5188382	0.6652608
Dispersion	σ	0.1850701	0.025915	0.1457457	0.2536259

Measures	
-2*LogLikelihood	-15.47643
AICc	-10.97643
BIC	-8.884759

Goodness-of-Fit Test

	W	Prob<W
Shapiro-Wilk	0.9500628	0.2151

	A²	Simulated p-Value
Anderson-Darling	0.4929529	0.2044

Note: Ho = The data is from the Normal distribution. Small p-values reject Ho.

Distributions Herd=mid-performing, Indicator=CC-biogenic kgCO2eq

Feeds purchase

Compare Distributions

Show	Distribution		AICc ^	BIC	-2*LogLikelihood
☑	Normal		-180.0662	-177.9745	-184.5662

Summary Statistics

Mean	0.0106809
Std Dev	0.0080804
Std Err Mean	0.0015551
Upper 95% Mean	0.0138774
Lower 95% Mean	0.0074844
N	27
N Missing	0

Fitted Normal Distribution

Parameter		Estimate	Std Error	Lower 95%	Upper 95%
Location	μ	0.0106809	0.0015551	0.0074844	0.0138774
Dispersion	σ	0.0080804	0.0011315	0.0063635	0.0110737

Measures	
-2*LogLikelihood	-184.5662
AICc	-180.0662
BIC	-177.9745

Goodness-of-Fit Test

	W	Prob<W
Shapiro-Wilk	0.8941934	0.0099*

	A²	Simulated p-Value
Anderson-Darling	1.0533585	0.0060*

Note: Ho = The data is from the Normal distribution. Small p-values reject Ho.

Distributions Herd=mid-performing, Indicator=CC-fossil kgCO2eq

Feeds purchase

Compare Distributions

Show	Distribution		AICc ^	BIC	-2*LogLikelihood
☑	Normal		-26.02542	-23.93375	-30.52542

Summary Statistics

Mean	0.2923932
Std Dev	0.140057
Std Err Mean	0.026954
Upper 95% Mean	0.347798
Lower 95% Mean	0.2369885
N	27
N Missing	0

Fitted Normal Distribution

Parameter		Estimate	Std Error	Lower 95%	Upper 95%
Location	μ	0.2923932	0.026954	0.2369885	0.347798
Dispersion	σ	0.140057	0.0196119	0.1102972	0.1919386

Measures	
-2*LogLikelihood	-30.52542
AICc	-26.02542
BIC	-23.93375

Goodness-of-Fit Test

	W	Prob<W
Shapiro-Wilk	0.8474367	0.0010*

	A²	Simulated p-Value
Anderson-Darling	0.9549682	0.0124*

Note: Ho = The data is from the Normal distribution. Small p-values reject Ho.

Distributions Herd=mid-performing, Indicator=CC-LTU kgCO2eq

Feeds purchase

Compare Distributions

Show	Distribution		AICc ^	BIC	-2*LogLikelihood
☑	Normal		-43.86639	-41.77472	-48.36639

Summary Statistics

Mean	0.2889754
Std Dev	0.1006512
Std Err Mean	0.0193703
Upper 95% Mean	0.3287917
Lower 95% Mean	0.2491591
N	27
N Missing	0

Fitted Normal Distribution

Parameter		Estimate	Std Error	Lower 95%	Upper 95%
Location	μ	0.2889754	0.0193703	0.2491591	0.3287917
Dispersion	σ	0.1006512	0.014094	0.0792645	0.1379356

Measures	
-2*LogLikelihood	-48.36639
AICc	-43.86639
BIC	-41.77472

Goodness-of-Fit Test

	W	Prob<W
Shapiro-Wilk	0.9031212	0.0158*

	A²	Simulated p-Value
Anderson-Darling	0.9809383	0.0156*

Note: Ho = The data is from the Normal distribution. Small p-values reject Ho.

Distributions Herd=mid-performing, Indicator=POCP kgNMVOCeq

Feeds purchase

Compare Distributions

Show	Distribution		AICc ^	BIC	-2*LogLikelihood
☑	Normal		-337.0567	-334.965	-341.5567

Summary Statistics

Mean	0.0008625
Std Dev	0.0004414
Std Err Mean	8.4949e-5
Upper 95% Mean	0.0010371
Lower 95% Mean	0.0006879
N	27
N Missing	0

Fitted Normal Distribution

Parameter		Estimate	Std Error	Lower 95%	Upper 95%
Location	μ	0.0008625	8.4949e-5	0.0006879	0.0010371
Dispersion	σ	0.0004414	0.0000618	0.0003476	0.0006049

Measures	
-2*LogLikelihood	-341.5567
AICc	-337.0567
BIC	-334.965

Goodness-of-Fit Test

	W	Prob<W
Shapiro-Wilk	0.7980053	0.0001*

	A²	Simulated p-Value
Anderson-Darling	1.3335555	0.0012*

Note: Ho = The data is from the Normal distribution. Small p-values reject Ho.
