# Supplementary material for: Climate Change and Photochemical Ozone Creation Potential Impact Indicators of Cow Milk: A Comparison of Different Scenarios for a Diet Assessment
Source: Animals (Basel). 2024 Jun 7;14(12):1725. doi: 10.3390/ani14121725 (PMC11201073; doi:10.3390/ani14121725)
Supplement: Supplementary file 1 [file animals-14-01725-s001.zip › animals-3004812-supplementary/Table 3/Distribution of In farm water use.pdf]

Distributions Herd=high-performing, Indicator=CC kgCO2eq

In farm water use

Compare Distributions

Show	Distribution		AICc ^	BIC	-2*LogLikelihood
☑	Normal	—	-179.5866	-179.3994	-184.6775

Summary Statistics

Mean	0.0013012
Std Dev	0.0003426
Std Err Mean	9.1573e-5
Upper 95% Mean	0.001499
Lower 95% Mean	0.0011034
N	14
N Missing	0

Fitted Normal Distribution

Parameter		Estimate	Std Error	Lower 95%	Upper 95%
Location	μ	0.0013012	9.1573e-5	0.0011034	0.001499
Dispersion	σ	0.0003426	6.8527e-5	0.0002484	0.000552

Measures	
-2*LogLikelihood	-184.6775
AICc	-179.5866
BIC	-179.3994

Goodness-of-Fit Test

	W	Prob<W
Shapiro-Wilk	0.8662123	0.0371*

	A²	Simulated p-Value
Anderson-Darling	0.8989268	0.0168*

Note: Ho = The data is from the Normal distribution. Small p-values reject Ho.

Distributions Herd=high-performing, Indicator=CC-biogenic kgCO2eq

In farm water use

Compare Distributions

Show	Distribution		AICc ^	BIC	-2*LogLikelihood
☑	Normal	—	-338.8951	-338.7079	-343.986

Summary Statistics

Mean	0.0000044
Std Dev	1.1584e-6
Std Err Mean	3.0961e-7
Upper 95% Mean	5.0682e-6
Lower 95% Mean	3.7305e-6
N	14
N Missing	0

Fitted Normal Distribution

Parameter		Estimate	Std Error	Lower 95%	Upper 95%
Location	μ	0.0000044	3.0961e-7	3.7305e-6	5.0682e-6
Dispersion	σ	1.1584e-6	2.3169e-7	8.3981e-7	1.8663e-6

Measures	
-2*LogLikelihood	-343.986
AICc	-338.8951
BIC	-338.7079

Goodness-of-Fit Test

	W	Prob<W
Shapiro-Wilk	0.8662122	0.0371*

	A²	Simulated p-Value
Anderson-Darling	0.898927	0.0160*

Note: Ho = The data is from the Normal distribution. Small p-values reject Ho.

Distributions Herd=high-performing, Indicator=CC-fossil kgCO2eq

In farm water use

Compare Distributions

Show	Distribution		AICc ^	BIC	-2*LogLikelihood
☑	Normal	—	-179.7031	-179.5159	-184.794

Summary Statistics

Mean	0.0012958
Std Dev	0.0003412
Std Err Mean	0.0000912
Upper 95% Mean	0.0014928
Lower 95% Mean	0.0010988
N	14
N Missing	0

Fitted Normal Distribution

Parameter		Estimate	Std Error	Lower 95%	Upper 95%
Location	μ	0.0012958	0.0000912	0.0010988	0.0014928
Dispersion	σ	0.0003412	6.8242e-5	0.0002474	0.0005497

Measures	
-2*LogLikelihood	-184.794
AICc	-179.7031
BIC	-179.5159

Goodness-of-Fit Test

	W	Prob<W
Shapiro-Wilk	0.8662122	0.0371*

	A²	Simulated p-Value
Anderson-Darling	0.898927	0.0120*

Note: Ho = The data is from the Normal distribution. Small p-values reject Ho.

Distributions Herd=high-performing, Indicator=CC-LTU kgCO2eq

In farm water use

Compare Distributions

Show	Distribution		AICc ^	BIC	-2*LogLikelihood
☑	Normal	—	-380.2989	-380.1117	-385.3898

Summary Statistics

Mean	1.0027e-6
Std Dev	2.6404e-7
Std Err Mean	7.0569e-8
Upper 95% Mean	1.1552e-6
Lower 95% Mean	8.5029e-7
N	14
N Missing	0

Fitted Normal Distribution

Parameter		Estimate	Std Error	Lower 95%	Upper 95%
Location	μ	1.0027e-6	7.0569e-8	8.5029e-7	1.1552e-6
Dispersion	σ	2.6404e-7	5.2809e-8	1.9142e-7	4.2539e-7

Measures	
-2*LogLikelihood	-385.3898
AICc	-380.2989
BIC	-380.1117

Goodness-of-Fit Test

	W	Prob<W
Shapiro-Wilk	0.8662122	0.0371*

	A²	Simulated p-Value
Anderson-Darling	0.898927	0.0140*

Note: Ho = The data is from the Normal distribution. Small p-values reject Ho.

Distributions Herd=high-performing, Indicator=POCP kgNMVOCeq

In farm water use

Compare Distributions

Show	Distribution		AICc ^	BIC	-2*LogLikelihood
☑	Normal	—	-344.8478	-344.6606	-349.9388

Summary Statistics

Mean	3.5568e-6
Std Dev	9.3657e-7
Std Err Mean	2.5031e-7
Upper 95% Mean	4.0975e-6
Lower 95% Mean	3.016e-6
N	14
N Missing	0

Fitted Normal Distribution

Parameter		Estimate	Std Error	Lower 95%	Upper 95%
Location	μ	3.5568e-6	2.5031e-7	3.016e-6	4.0975e-6
Dispersion	σ	9.3657e-7	1.8731e-7	6.7897e-7	1.5089e-6

Measures	
-2*LogLikelihood	-349.9388
AICc	-344.8478
BIC	-344.6606

Goodness-of-Fit Test

	W	Prob<W
Shapiro-Wilk	0.8662122	0.0371*

	A²	Simulated p-Value
Anderson-Darling	0.898927	0.0172*

Note: Ho = The data is from the Normal distribution. Small p-values reject Ho.

Distributions Herd=low-performing, Indicator=CC kgCO2eq

In farm water use

Compare Distributions

Show	Distribution		AICc ^	BIC	-2*LogLikelihood
☑	Normal	—	-187.0588	-186.8716	-192.1497

Summary Statistics

Mean	0.0021736
Std Dev	0.0002624
Std Err Mean	7.0124e-5
Upper 95% Mean	0.0023251
Lower 95% Mean	0.0020221
N	14
N Missing	0

Fitted Normal Distribution

Parameter		Estimate	Std Error	Lower 95%	Upper 95%
Location	μ	0.0021736	7.0124e-5	0.0020221	0.0023251
Dispersion	σ	0.0002624	5.2476e-5	0.0001902	0.0004227

Measures	
-2*LogLikelihood	-192.1497
AICc	-187.0588
BIC	-186.8716

Goodness-of-Fit Test

	W	Prob<W
Shapiro-Wilk	0.9683447	0.8538

	A²	Simulated p-Value
Anderson-Darling	0.2837256	0.6120

Note: Ho = The data is from the Normal distribution. Small p-values reject Ho.

Distributions Herd=low-performing, Indicator=CC-biogenic kgCO2eq

In farm water use

Compare Distributions

Show	Distribution		AICc ^	BIC	-2*LogLikelihood
☑	Normal	—	-346.3672	-346.18	-351.4582

Summary Statistics

Mean	7.3488e-6
Std Dev	8.8711e-7
Std Err Mean	2.3709e-7
Upper 95% Mean	7.861e-6
Lower 95% Mean	6.8366e-6
N	14
N Missing	0

Fitted Normal Distribution

Parameter		Estimate	Std Error	Lower 95%	Upper 95%
Location	μ	7.3488e-6	2.3709e-7	6.8366e-6	7.861e-6
Dispersion	σ	8.8711e-7	1.7742e-7	6.4311e-7	1.4292e-6

Measures	
-2*LogLikelihood	-351.4582
AICc	-346.3672
BIC	-346.18

Goodness-of-Fit Test

	W	Prob<W
Shapiro-Wilk	0.9683447	0.8538

	A²	Simulated p-Value
Anderson-Darling	0.2837255	0.6200

Note: Ho = The data is from the Normal distribution. Small p-values reject Ho.

Distributions Herd=low-performing, Indicator=CC-fossil kgCO2eq

In farm water use

Compare Distributions

Show	Distribution		AICc ^	BIC	-2*LogLikelihood
☑	Normal	—	-187.1753	-186.9881	-192.2662

Summary Statistics

Mean	0.0021645
Std Dev	0.0002613
Std Err Mean	6.9833e-5
Upper 95% Mean	0.0023154
Lower 95% Mean	0.0020137
N	14
N Missing	0

Fitted Normal Distribution

Parameter		Estimate	Std Error	Lower 95%	Upper 95%
Location	μ	0.0021645	6.9833e-5	0.0020137	0.0023154
Dispersion	σ	0.0002613	5.2258e-5	0.0001894	0.000421

Measures	
-2*LogLikelihood	-192.2662
AICc	-187.1753
BIC	-186.9881

Goodness-of-Fit Test

	W	Prob<W
Shapiro-Wilk	0.9683447	0.8538

	A²	Simulated p-Value
Anderson-Darling	0.2837255	0.6132

Note: Ho = The data is from the Normal distribution. Small p-values reject Ho.

Distributions Herd=low-performing, Indicator=CC-LTU kgCO2eq

In farm water use

Compare Distributions

Show	Distribution		AICc ^	BIC	-2*LogLikelihood
☑	Normal	—	-387.7711	-387.5839	-392.862

Summary Statistics

Mean	1.675e-6
Std Dev	2.022e-7
Std Err Mean	5.404e-8
Upper 95% Mean	1.7918e-6
Lower 95% Mean	1.5583e-6
N	14
N Missing	0

Fitted Normal Distribution

Parameter		Estimate	Std Error	Lower 95%	Upper 95%
Location	μ	1.675e-6	5.404e-8	1.5583e-6	1.7918e-6
Dispersion	σ	2.022e-7	4.044e-8	1.4659e-7	3.2575e-7

Measures	
-2*LogLikelihood	-392.862
AICc	-387.7711
BIC	-387.5839

Goodness-of-Fit Test

	W	Prob<W
Shapiro-Wilk	0.9683447	0.8538

	A²	Simulated p-Value
Anderson-Darling	0.2837255	0.6060

Note: Ho = The data is from the Normal distribution. Small p-values reject Ho.

Distributions Herd=low-performing, Indicator=POCP kgNMVOCeq

In farm water use

Compare Distributions

Show	Distribution		AICc ^	BIC	-2*LogLikelihood
☑	Normal	—	-352.32	-352.1328	-357.4109

Summary Statistics

Mean	5.9414e-6
Std Dev	7.1721e-7
Std Err Mean	1.9168e-7
Upper 95% Mean	6.3555e-6
Lower 95% Mean	5.5273e-6
N	14
N Missing	0

Fitted Normal Distribution

Parameter		Estimate	Std Error	Lower 95%	Upper 95%
Location	μ	5.9414e-6	1.9168e-7	5.5273e-6	6.3555e-6
Dispersion	σ	7.1721e-7	1.4344e-7	5.1994e-7	1.1555e-6

Measures	
-2*LogLikelihood	-357.4109
AICc	-352.32
BIC	-352.1328

Goodness-of-Fit Test

	W	Prob<W
Shapiro-Wilk	0.9683447	0.8538

	A²	Simulated p-Value
Anderson-Darling	0.2837255	0.6072

Note: Ho = The data is from the Normal distribution. Small p-values reject Ho.

Distributions Herd=mid-performing, Indicator=CC kgCO2eq

In farm water use

Compare Distributions

Show	Distribution		AICc ^	BIC	-2*LogLikelihood
☑	Normal	—	-384.6513	-382.5597	-389.1513

Summary Statistics

Mean	0.0016674
Std Dev	0.0001828
Std Err Mean	3.5187e-5
Upper 95% Mean	0.0017397
Lower 95% Mean	0.0015951
N	27
N Missing	0

Fitted Normal Distribution

Parameter		Estimate	Std Error	Lower 95%	Upper 95%
Location	μ	0.0016674	3.5187e-5	0.0015951	0.0017397
Dispersion	σ	0.0001828	0.0000256	0.000144	0.0002506

Measures	
-2*LogLikelihood	-389.1513
AICc	-384.6513
BIC	-382.5597

Goodness-of-Fit Test

	W	Prob<W
Shapiro-Wilk	0.9718624	0.6516

	A²	Simulated p-Value
Anderson-Darling	0.2551806	0.7196

Note: Ho = The data is from the Normal distribution. Small p-values reject Ho.

Distributions Herd=mid-performing, Indicator=CC-biogenic kgCO2eq

In farm water use

Compare Distributions

Show	Distribution		AICc ^	BIC	-2*LogLikelihood
☑	Normal	—	-691.889	-689.7973	-696.389

Summary Statistics

Mean	5.6375e-6
Std Dev	6.1816e-7
Std Err Mean	1.1897e-7
Upper 95% Mean	5.882e-6
Lower 95% Mean	5.393e-6
N	27
N Missing	0

Fitted Normal Distribution

Parameter		Estimate	Std Error	Lower 95%	Upper 95%
Location	μ	5.6375e-6	1.1897e-7	5.393e-6	5.882e-6
Dispersion	σ	6.1816e-7	8.656e-8	4.8681e-7	8.4715e-7

Measures	
-2*LogLikelihood	-696.389
AICc	-691.889
BIC	-689.7973

Goodness-of-Fit Test

	W	Prob<W
Shapiro-Wilk	0.9718624	0.6516

	A²	Simulated p-Value
Anderson-Darling	0.2551806	0.7172

Note: Ho = The data is from the Normal distribution. Small p-values reject Ho.

Distributions Herd=mid-performing, Indicator=CC-fossil kgCO2eq

In farm water use

Compare Distributions

Show	Distribution		AICc ^	BIC	-2*LogLikelihood
☑	Normal	—	-384.876	-382.7843	-389.376

Summary Statistics

Mean	0.0016605
Std Dev	0.0001821
Std Err Mean	3.5041e-5
Upper 95% Mean	0.0017325
Lower 95% Mean	0.0015885
N	27
N Missing	0

Fitted Normal Distribution

Parameter		Estimate	Std Error	Lower 95%	Upper 95%
Location	μ	0.0016605	3.5041e-5	0.0015885	0.0017325
Dispersion	σ	0.0001821	0.0000255	0.0001434	0.0002495

Measures	
-2*LogLikelihood	-389.376
AICc	-384.876
BIC	-382.7843

Goodness-of-Fit Test

	W	Prob<W
Shapiro-Wilk	0.9718624	0.6516

	A²	Simulated p-Value
Anderson-Darling	0.2551807	0.7124

Note: Ho = The data is from the Normal distribution. Small p-values reject Ho.

Distributions Herd=mid-performing, Indicator=CC-LTU kgCO2eq

In farm water use

Compare Distributions

Show	Distribution		AICc ^	BIC	-2*LogLikelihood
☑	Normal	—	-771.7393	-769.6477	-776.2393

Summary Statistics

Mean	1.285e-6
Std Dev	1.409e-7
Std Err Mean	2.7116e-8
Upper 95% Mean	1.3407e-6
Lower 95% Mean	1.2292e-6
N	27
N Missing	0

Fitted Normal Distribution

Parameter		Estimate	Std Error	Lower 95%	Upper 95%
Location	μ	1.285e-6	2.7116e-8	1.2292e-6	1.3407e-6
Dispersion	σ	1.409e-7	1.973e-8	1.1096e-7	1.9309e-7

Measures	
-2*LogLikelihood	-776.2393
AICc	-771.7393
BIC	-769.6477

Goodness-of-Fit Test

	W	Prob<W
Shapiro-Wilk	0.9718624	0.6516

	A²	Simulated p-Value
Anderson-Darling	0.2551806	0.7268

Note: Ho = The data is from the Normal distribution. Small p-values reject Ho.

Distributions Herd=mid-performing, Indicator=POCP kgNMVOCeq

In farm water use

Compare Distributions

Show	Distribution		AICc ^	BIC	-2*LogLikelihood
☑	Normal	—	-703.3694	-701.2777	-707.8694

Summary Statistics

Mean	4.5578e-6
Std Dev	4.9977e-7
Std Err Mean	9.6181e-8
Upper 95% Mean	4.7555e-6
Lower 95% Mean	4.3601e-6
N	27
N Missing	0

Fitted Normal Distribution

Parameter		Estimate	Std Error	Lower 95%	Upper 95%
Location	μ	4.5578e-6	9.6181e-8	4.3601e-6	4.7555e-6
Dispersion	σ	4.9977e-7	6.9982e-8	3.9358e-7	6.849e-7

Measures	
-2*LogLikelihood	-707.8694
AICc	-703.3694
BIC	-701.2777

Goodness-of-Fit Test

	W	Prob<W
Shapiro-Wilk	0.9718624	0.6516

	A²	Simulated p-Value
Anderson-Darling	0.2551806	0.7020

Note: Ho = The data is from the Normal distribution. Small p-values reject Ho.
