# Supplementary material for: Climate Change and Photochemical Ozone Creation Potential Impact Indicators of Cow Milk: A Comparison of Different Scenarios for a Diet Assessment
Source: Animals (Basel). 2024 Jun 7;14(12):1725. doi: 10.3390/ani14121725 (PMC11201073; doi:10.3390/ani14121725)
Supplement: Supplementary file 1 [file animals-14-01725-s001.zip › animals-3004812-supplementary/Table 3/Distribution of In-farm feeds.pdf]

Distributions Herd=high-performing, Indicator=CC kgCO2eq

In-farm feeds

Compare Distributions

Show	Distribution		AICc ^	BIC	-2*LogLikelihood
☑	Normal		-64.96246	-64.77526	-70.05337

Summary Statistics

Mean	0.0590892
Std Dev	0.0205452
Std Err Mean	0.0054909
Upper 95% Mean	0.0709516
Lower 95% Mean	0.0472267
N	14
N Missing	0

Fitted Normal Distribution

Parameter		Estimate	Std Error	Lower 95%	Upper 95%
Location	μ	0.0590892	0.0054909	0.0472267	0.0709516
Dispersion	σ	0.0205452	0.004109	0.0148943	0.0330991

Measures	
-2*LogLikelihood	-70.05337
AICc	-64.96246
BIC	-64.77526

Goodness-of-Fit Test

	W	Prob<W
Shapiro-Wilk	0.9682484	0.8524

	A^2	Simulated p-Value
Anderson-Darling	0.1873231	0.8972

Note: Ho = The data is from the Normal distribution. Small p-values reject Ho.

Distributions Herd=high-performing, Indicator=CC-biogenic kgCO2eq

In-farm feeds

Compare Distributions

Show	Distribution		AICc ^	BIC	-2*LogLikelihood
☑	Normal		-316.3636	-316.1763	-321.4545

Summary Statistics

Mean	3.3676e-6
Std Dev	2.5903e-6
Std Err Mean	6.9228e-7
Upper 95% Mean	4.8632e-6
Lower 95% Mean	1.872e-6
N	14
N Missing	0

Fitted Normal Distribution

Parameter		Estimate	Std Error	Lower 95%	Upper 95%
Location	μ	3.3676e-6	6.9228e-7	1.872e-6	4.8632e-6
Dispersion	σ	2.5903e-6	5.1806e-7	1.8778e-6	4.1731e-6

Measures	
-2*LogLikelihood	-321.4545
AICc	-316.3636
BIC	-316.1763

Goodness-of-Fit Test

	W	Prob<W
Shapiro-Wilk	0.9311722	0.3169

	A^2	Simulated p-Value
Anderson-Darling	0.3554444	0.4252

Note: Ho = The data is from the Normal distribution. Small p-values reject Ho.

Distributions Herd=high-performing, Indicator=CC-fossil kgCO2eq

In-farm feeds

Compare Distributions

Show	Distribution		AICc ^	BIC	-2*LogLikelihood
☑	Normal		-64.97651	-64.78931	-70.06742

Summary Statistics

Mean	0.0590669
Std Dev	0.0205348
Std Err Mean	0.0054882
Upper 95% Mean	0.0709233
Lower 95% Mean	0.0472104
N	14
N Missing	0

Fitted Normal Distribution

Parameter		Estimate	Std Error	Lower 95%	Upper 95%
Location	μ	0.0590669	0.0054882	0.0472104	0.0709233
Dispersion	σ	0.0205348	0.004107	0.0148868	0.0330825

Measures	
-2*LogLikelihood	-70.06742
AICc	-64.97651
BIC	-64.78931

Goodness-of-Fit Test

	W	Prob<W
Shapiro-Wilk	0.9682016	0.8517

	A^2	Simulated p-Value
Anderson-Darling	0.1875143	0.9012

Note: Ho = The data is from the Normal distribution. Small p-values reject Ho.

Distributions Herd=high-performing, Indicator=CC-LTU kgCO2eq

In-farm feeds

Compare Distributions

Show	Distribution		AICc ^	BIC	-2*LogLikelihood
☑	Normal		-264.9614	-264.7741	-270.0523

Summary Statistics

Mean	1.8943e-5
Std Dev	1.6241e-5
Std Err Mean	4.3407e-6
Upper 95% Mean	2.8321e-5
Lower 95% Mean	9.5655e-6
N	14
N Missing	0

Fitted Normal Distribution

Parameter		Estimate	Std Error	Lower 95%	Upper 95%
Location	μ	1.8943e-5	4.3407e-6	9.5655e-6	2.8321e-5
Dispersion	σ	1.6241e-5	3.2483e-6	1.1774e-5	2.6166e-5

Measures	
-2*LogLikelihood	-270.0523
AICc	-264.9614
BIC	-264.7741

Goodness-of-Fit Test

	W	Prob<W
Shapiro-Wilk	0.899802	0.1120

	A^2	Simulated p-Value
Anderson-Darling	0.4300898	0.2864

Note: Ho = The data is from the Normal distribution. Small p-values reject Ho.

Distributions Herd=high-performing, Indicator=POCP kgNMVOCeq

In-farm feeds

Compare Distributions

Show	Distribution		AICc ^	BIC	-2*LogLikelihood
☑	Normal		-283.7786	-283.5914	-288.8696

Summary Statistics

Mean	0.0000107
Std Dev	8.2939e-6
Std Err Mean	2.2166e-6
Upper 95% Mean	1.5479e-5
Lower 95% Mean	5.9019e-6
N	14
N Missing	0

Fitted Normal Distribution

Parameter		Estimate	Std Error	Lower 95%	Upper 95%
Location	μ	0.0000107	2.2166e-6	5.9019e-6	1.5479e-5
Dispersion	σ	8.2939e-6	1.6588e-6	6.0127e-6	1.3362e-5

Measures	
-2*LogLikelihood	-288.8696
AICc	-283.7786
BIC	-283.5914

Goodness-of-Fit Test

	W	Prob<W
Shapiro-Wilk	0.9115274	0.1658

	A^2	Simulated p-Value
Anderson-Darling	0.4753025	0.2056

Note: Ho = The data is from the Normal distribution. Small p-values reject Ho.

Distributions Herd=low-performing, Indicator=CC kgCO2eq

In-farm feeds

Compare Distributions

Show	Distribution		AICc ^	BIC	-2*LogLikelihood
☑	Normal		-55.27837	-55.09117	-60.36928

Summary Statistics

Mean	0.1157954
Std Dev	0.0290345
Std Err Mean	0.0077598
Upper 95% Mean	0.1325594
Lower 95% Mean	0.0990313
N	14
N Missing	0

Fitted Normal Distribution

Parameter		Estimate	Std Error	Lower 95%	Upper 95%
Location	μ	0.1157954	0.0077598	0.0990313	0.1325594
Dispersion	σ	0.0290345	0.0058069	0.0210487	0.0467758

Measures	
-2*LogLikelihood	-60.36928
AICc	-55.27837
BIC	-55.09117

Goodness-of-Fit Test

	W	Prob<W
Shapiro-Wilk	0.8697974	0.0417*

	A^2	Simulated p-Value
Anderson-Darling	0.6675645	0.0552

Note: Ho = The data is from the Normal distribution. Small p-values reject Ho.

Distributions Herd=low-performing, Indicator=CC-biogenic kgCO2eq

In-farm feeds

Compare Distributions

Show	Distribution		AICc ^	BIC	-2*LogLikelihood
☑	Normal		-300.4695	-300.2822	-305.5604

Summary Statistics

Mean	1.0883e-5
Std Dev	4.5696e-6
Std Err Mean	1.2213e-6
Upper 95% Mean	1.3521e-5
Lower 95% Mean	8.2442e-6
N	14
N Missing	0

Fitted Normal Distribution

Parameter		Estimate	Std Error	Lower 95%	Upper 95%
Location	μ	1.0883e-5	1.2213e-6	8.2442e-6	1.3521e-5
Dispersion	σ	4.5696e-6	9.1391e-7	3.3127e-6	7.3617e-6

Measures	
-2*LogLikelihood	-305.5604
AICc	-300.4695
BIC	-300.2822

Goodness-of-Fit Test

	W	Prob<W
Shapiro-Wilk	0.941121	0.4329

	A^2	Simulated p-Value
Anderson-Darling	0.300679	0.5724

Note: Ho = The data is from the Normal distribution. Small p-values reject Ho.

Distributions Herd=low-performing, Indicator=CC-fossil kgCO2eq

In-farm feeds

Compare Distributions

Show	Distribution		AICc ^	BIC	-2*LogLikelihood
☑	Normal		-55.29088	-55.10367	-60.38179

Summary Statistics

Mean	0.1157365
Std Dev	0.0290215
Std Err Mean	0.0077563
Upper 95% Mean	0.132493
Lower 95% Mean	0.09898
N	14
N Missing	0

Fitted Normal Distribution

Parameter		Estimate	Std Error	Lower 95%	Upper 95%
Location	μ	0.1157365	0.0077563	0.09898	0.132493
Dispersion	σ	0.0290215	0.0058043	0.0210393	0.0467549

Measures	
-2*LogLikelihood	-60.38179
AICc	-55.29088
BIC	-55.10367

Goodness-of-Fit Test

	W	Prob<W
Shapiro-Wilk	0.869629	0.0415*

	A^2	Simulated p-Value
Anderson-Darling	0.6687508	0.0632

Note: Ho = The data is from the Normal distribution. Small p-values reject Ho.

Distributions Herd=low-performing, Indicator=CC-LTU kgCO2eq

In-farm feeds

Compare Distributions

Show	Distribution		AICc ^	BIC	-2*LogLikelihood
☑	Normal		-251.5031	-251.3159	-256.594

Summary Statistics

Mean	4.7982e-5
Std Dev	2.6264e-5
Std Err Mean	7.0195e-6
Upper 95% Mean	6.3147e-5
Lower 95% Mean	3.2817e-5
N	14
N Missing	0

Fitted Normal Distribution

Parameter		Estimate	Std Error	Lower 95%	Upper 95%
Location	μ	4.7982e-5	7.0195e-6	3.2817e-5	6.3147e-5
Dispersion	σ	2.6264e-5	5.2529e-6	1.904e-5	4.2313e-5

Measures	
-2*LogLikelihood	-256.594
AICc	-251.5031
BIC	-251.3159

Goodness-of-Fit Test

	W	Prob<W
Shapiro-Wilk	0.892356	0.0874

	A^2	Simulated p-Value
Anderson-Darling	0.623761	0.0780

Note: Ho = The data is from the Normal distribution. Small p-values reject Ho.

Distributions Herd=low-performing, Indicator=POCP kgNMVOCeq

In-farm feeds

Compare Distributions

Show	Distribution		AICc ^	BIC	-2*LogLikelihood
☑	Normal		-263.9387	-263.7515	-269.0296

Summary Statistics

Mean	3.8462e-5
Std Dev	1.6846e-5
Std Err Mean	4.5022e-6
Upper 95% Mean	4.8188e-5
Lower 95% Mean	2.8736e-5
N	14
N Missing	0

Fitted Normal Distribution

Parameter		Estimate	Std Error	Lower 95%	Upper 95%
Location	μ	3.8462e-5	4.5022e-6	2.8736e-5	4.8188e-5
Dispersion	σ	1.6846e-5	3.3691e-6	1.2212e-5	2.7139e-5

Measures	
-2*LogLikelihood	-269.0296
AICc	-263.9387
BIC	-263.7515

Goodness-of-Fit Test

	W	Prob<W
Shapiro-Wilk	0.9356494	0.3654

	A^2	Simulated p-Value
Anderson-Darling	0.3226409	0.5180

Note: Ho = The data is from the Normal distribution. Small p-values reject Ho.

Distributions Herd=mid-performing, Indicator=CC kgCO2eq

In-farm feeds

Compare Distributions

Show	Distribution		AICc ^	BIC	-2*LogLikelihood
☑	Normal		-87.64878	-85.55711	-92.14878

Summary Statistics

Mean	0.0873734
Std Dev	0.0447404
Std Err Mean	0.0086103
Upper 95% Mean	0.1050721
Lower 95% Mean	0.0696747
N	27
N Missing	0

Fitted Normal Distribution

Parameter		Estimate	Std Error	Lower 95%	Upper 95%
Location	μ	0.0873734	0.0086103	0.0696747	0.1050721
Dispersion	σ	0.0447404	0.0062649	0.0352338	0.0613136

Measures	
-2*LogLikelihood	-92.14878
AICc	-87.64878
BIC	-85.55711

Goodness-of-Fit Test

	W	Prob<W
Shapiro-Wilk	0.7258461	<.0001*

	A^2	Simulated p-Value
Anderson-Darling	1.9457952	<.0001*

Note: Ho = The data is from the Normal distribution. Small p-values reject Ho.

Distributions Herd=mid-performing, Indicator=CC-biogenic kgCO2eq

In-farm feeds

Compare Distributions

Show	Distribution		AICc ^	BIC	-2*LogLikelihood
☑	Normal		-534.3145	-532.2228	-538.8145

Summary Statistics

Mean	7.54e-6
Std Dev	1.1439e-5
Std Err Mean	2.2015e-6
Upper 95% Mean	1.2065e-5
Lower 95% Mean	3.0148e-6
N	27
N Missing	0

Fitted Normal Distribution

Parameter		Estimate	Std Error	Lower 95%	Upper 95%
Location	μ	7.54e-6	2.2015e-6	3.0148e-6	1.2065e-5
Dispersion	σ	1.1439e-5	1.6018e-6	9.0085e-6	1.5677e-5

Measures	
-2*LogLikelihood	-538.8145
AICc	-534.3145
BIC	-532.2228

Goodness-of-Fit Test

	W	Prob<W
Shapiro-Wilk	0.5334775	<.0001*

	A^2	Simulated p-Value
Anderson-Darling	4.1371531	<.0001*

Note: Ho = The data is from the Normal distribution. Small p-values reject Ho.

Distributions Herd=mid-performing, Indicator=CC-fossil kgCO2eq

In-farm feeds

Compare Distributions

Show	Distribution		AICc ^	BIC	-2*LogLikelihood
☑	Normal		-87.68891	-85.59724	-92.18891

Summary Statistics

Mean	0.0873387
Std Dev	0.0447071
Std Err Mean	0.0086039
Upper 95% Mean	0.1050243
Lower 95% Mean	0.0696532
N	27
N Missing	0

Fitted Normal Distribution

Parameter		Estimate	Std Error	Lower 95%	Upper 95%
Location	μ	0.0873387	0.0086039	0.0696532	0.1050243
Dispersion	σ	0.0447071	0.0062603	0.0352076	0.0612681

Measures	
-2*LogLikelihood	-92.18891
AICc	-87.68891
BIC	-85.59724

Goodness-of-Fit Test

	W	Prob<W
Shapiro-Wilk	0.7259367	<.0001*

	A^2	Simulated p-Value
Anderson-Darling	1.9451659	<.0001*

Note: Ho = The data is from the Normal distribution. Small p-values reject Ho.

Distributions Herd=mid-performing, Indicator=CC-LTU kgCO2eq

In-farm feeds

Compare Distributions

Show	Distribution		AICc ^	BIC	-2*LogLikelihood
☑	Normal		-488.2642	-486.1725	-492.7642

Summary Statistics

Mean	0.0000271
Std Dev	2.6838e-5
Std Err Mean	5.165e-6
Upper 95% Mean	3.7727e-5
Lower 95% Mean	0.0000165
N	27
N Missing	0

Fitted Normal Distribution

Parameter		Estimate	Std Error	Lower 95%	Upper 95%
Location	μ	0.0000271	5.165e-6	0.0000165	3.7727e-5
Dispersion	σ	2.6838e-5	3.7581e-6	2.1136e-5	3.678e-5

Measures	
-2*LogLikelihood	-492.7642
AICc	-488.2642
BIC	-486.1725

Goodness-of-Fit Test

	W	Prob<W
Shapiro-Wilk	0.7935632	0.0001*

	A^2	Simulated p-Value
Anderson-Darling	1.1219873	0.0044*

Note: Ho = The data is from the Normal distribution. Small p-values reject Ho.

Distributions Herd=mid-performing, Indicator=POCP kgNMVOCeq

In-farm feeds

Compare Distributions

Show	Distribution		AICc ^	BIC	-2*LogLikelihood
☑	Normal		-457.1172	-455.0255	-461.6172

Summary Statistics

Mean	2.7387e-5
Std Dev	4.7781e-5
Std Err Mean	9.1954e-6
Upper 95% Mean	4.6289e-5
Lower 95% Mean	8.4857e-6
N	27
N Missing	0

Fitted Normal Distribution

Parameter		Estimate	Std Error	Lower 95%	Upper 95%
Location	μ	2.7387e-5	9.1954e-6	8.4857e-6	4.6289e-5
Dispersion	σ	4.7781e-5	6.6907e-6	3.7628e-5	6.548e-5

Measures	
-2*LogLikelihood	-461.6172
AICc	-457.1172
BIC	-455.0255

Goodness-of-Fit Test

	W	Prob<W
Shapiro-Wilk	0.486145	<.0001*

	A^2	Simulated p-Value
Anderson-Darling	4.8356468	<.0001*

Note: Ho = The data is from the Normal distribution. Small p-values reject Ho.
