# Supplementary material for: Climate Change and Photochemical Ozone Creation Potential Impact Indicators of Cow Milk: A Comparison of Different Scenarios for a Diet Assessment
Source: Animals (Basel). 2024 Jun 7;14(12):1725. doi: 10.3390/ani14121725 (PMC11201073; doi:10.3390/ani14121725)
Supplement: Supplementary file 1 [file animals-14-01725-s001.zip › animals-3004812-supplementary/Table 3/Distribution of Manure handling.pdf]

Distributions Herd=high-performing, Indicator=CC kgCO2eq

Manure handling

Compare Distributions

Show	Distribution	AICc ^	BIC	-2*LogLikelihood
☑	Normal	-10.55349	-10.36628	-15.64439

Summary Statistics

Mean	0.376791
Std Dev	0.1434236
Std Err Mean	0.0383316
Upper 95% Mean	0.4596014
Lower 95% Mean	0.2939807
N	14
N Missing	0

Fitted Normal Distribution

Parameter		Estimate	Std Error	Lower 95%	Upper 95%
Location	μ	0.376791	0.0383316	0.2939807	0.4596014
Dispersion	σ	0.1434236	0.0286847	0.1039755	0.2310615

Measures	
-2*LogLikelihood	-15.64439
AICc	-10.55349
BIC	-10.36628

Goodness-of-Fit Test

	W	Prob<W
Shapiro-Wilk	0.9010496	0.1168

	A²	Simulated p-Value
Anderson-Darling	0.5497677	0.1292

Note: Ho = The data is from the Normal distribution. Small p-values reject Ho.

Distributions Herd=high-performing, Indicator=CC-biogenic kgCO2eq

Manure handling

Compare Distributions

Show	Distribution	AICc ^	BIC	-2*LogLikelihood
☑	Normal	-11.73952	-11.55231	-16.83043

Summary Statistics

Mean	0.3314362
Std Dev	0.1374753
Std Err Mean	0.0367418
Upper 95% Mean	0.4108121
Lower 95% Mean	0.2520603
N	14
N Missing	0

Fitted Normal Distribution

Parameter		Estimate	Std Error	Lower 95%	Upper 95%
Location	μ	0.3314362	0.0367418	0.2520603	0.4108121
Dispersion	σ	0.1374753	0.0274951	0.0996633	0.2214786

Measures	
-2*LogLikelihood	-16.83043
AICc	-11.73952
BIC	-11.55231

Goodness-of-Fit Test

	W	Prob<W
Shapiro-Wilk	0.8932157	0.0899

	A²	Simulated p-Value
Anderson-Darling	0.5873435	0.1092

Note: Ho = The data is from the Normal distribution. Small p-values reject Ho.

Distributions Herd=high-performing, Indicator=CC-fossil kgCO2eq

Manure handling

Compare Distributions

Show	Distribution	AICc ^	BIC	-2*LogLikelihood
☑	Normal	-89.80507	-89.61787	-94.89598

Summary Statistics

Mean	0.0453549
Std Dev	0.0084603
Std Err Mean	0.0022611
Upper 95% Mean	0.0502397
Lower 95% Mean	0.04047
N	14
N Missing	0

Fitted Normal Distribution

Parameter		Estimate	Std Error	Lower 95%	Upper 95%
Location	μ	0.0453549	0.0022611	0.04047	0.0502397
Dispersion	σ	0.0084603	0.0016921	0.0061334	0.01363

Measures	
-2*LogLikelihood	-94.89598
AICc	-89.80507
BIC	-89.61787

Goodness-of-Fit Test

	W	Prob<W
Shapiro-Wilk	0.8748283	0.0491*

	A²	Simulated p-Value
Anderson-Darling	0.736446	0.0364*

Note: Ho = The data is from the Normal distribution. Small p-values reject Ho.

Distributions Herd=high-performing, Indicator=CC-LTU kgCO2eq

Manure handling

Summary Statistics

Mean	0
Std Dev	0
Std Err Mean	0
Upper 95% Mean	0
Lower 95% Mean	0
N	14
N Missing	0

Distributions Herd=high-performing, Indicator=POCP kgNMVOCeq

Manure handling

Compare Distributions

Show	Distribution	AICc ^	BIC	-2*LogLikelihood
☑	Normal	-239.1438	-238.9566	-244.2347

Summary Statistics

Mean	9.8456e-5
Std Dev	4.0838e-5
Std Err Mean	1.0914e-5
Upper 95% Mean	0.000122
Lower 95% Mean	7.4877e-5
N	14
N Missing	0

Fitted Normal Distribution

Parameter		Estimate	Std Error	Lower 95%	Upper 95%
Location	μ	9.8456e-5	1.0914e-5	7.4877e-5	0.000122
Dispersion	σ	4.0838e-5	8.1677e-6	0.0000296	0.0000658

Measures	
-2*LogLikelihood	-244.2347
AICc	-239.1438
BIC	-238.9566

Goodness-of-Fit Test

	W	Prob<W
Shapiro-Wilk	0.8932157	0.0899

	A²	Simulated p-Value
Anderson-Darling	0.5873435	0.1064

Note: Ho = The data is from the Normal distribution. Small p-values reject Ho.

Distributions Herd=low-performing, Indicator=CC kgCO2eq

Manure handling

Compare Distributions

Show	Distribution	AICc ^	BIC	-2*LogLikelihood
☑	Normal	-13.94095	-13.75374	-19.03186

Summary Statistics

Mean	0.4576251
Std Dev	0.1270806
Std Err Mean	0.0339637
Upper 95% Mean	0.5309993
Lower 95% Mean	0.384251
N	14
N Missing	0

Fitted Normal Distribution

Parameter		Estimate	Std Error	Lower 95%	Upper 95%
Location	μ	0.4576251	0.0339637	0.384251	0.5309993
Dispersion	σ	0.1270806	0.0254161	0.0921276	0.2047323

Measures	
-2*LogLikelihood	-19.03186
AICc	-13.94095
BIC	-13.75374

Goodness-of-Fit Test

	W	Prob<W
Shapiro-Wilk	0.9408829	0.4298

	A²	Simulated p-Value
Anderson-Darling	0.3878134	0.3336

Note: Ho = The data is from the Normal distribution. Small p-values reject Ho.

Distributions Herd=low-performing, Indicator=CC-biogenic kgCO2eq

Manure handling

Compare Distributions

Show	Distribution	AICc ^	BIC	-2*LogLikelihood
☑	Normal	-13.46851	-13.2813	-18.55941

Summary Statistics

Mean	0.3800936
Std Dev	0.1292431
Std Err Mean	0.0345417
Upper 95% Mean	0.4547163
Lower 95% Mean	0.3054708
N	14
N Missing	0

Fitted Normal Distribution

Parameter		Estimate	Std Error	Lower 95%	Upper 95%
Location	μ	0.3800936	0.0345417	0.3054708	0.4547163
Dispersion	σ	0.1292431	0.0258486	0.0936953	0.208216

Measures	
-2*LogLikelihood	-18.55941
AICc	-13.46851
BIC	-13.2813

Goodness-of-Fit Test

	W	Prob<W
Shapiro-Wilk	0.9518087	0.5891

	A²	Simulated p-Value
Anderson-Darling	0.2696198	0.6704

Note: Ho = The data is from the Normal distribution. Small p-values reject Ho.

Distributions Herd=low-performing, Indicator=CC-fossil kgCO2eq

Manure handling

Compare Distributions

Show	Distribution	AICc ^	BIC	-2*LogLikelihood
☑	Normal	-77.79091	-77.6037	-82.88182

Summary Statistics

Mean	0.0775316
Std Dev	0.0129937
Std Err Mean	0.0034727
Upper 95% Mean	0.0850339
Lower 95% Mean	0.0700292
N	14
N Missing	0

Fitted Normal Distribution

Parameter		Estimate	Std Error	Lower 95%	Upper 95%
Location	μ	0.0775316	0.0034727	0.0700292	0.0850339
Dispersion	σ	0.0129937	0.0025987	0.0094198	0.0209334

Measures	
-2*LogLikelihood	-82.88182
AICc	-77.79091
BIC	-77.6037

Goodness-of-Fit Test

	W	Prob<W
Shapiro-Wilk	0.9512616	0.5805

	A²	Simulated p-Value
Anderson-Darling	0.3585166	0.4132

Note: Ho = The data is from the Normal distribution. Small p-values reject Ho.

Distributions Herd=low-performing, Indicator=CC-LTU kgCO2eq

Manure handling

Summary Statistics

Mean	0
Std Dev	0
Std Err Mean	0
Upper 95% Mean	0
Lower 95% Mean	0
N	14
N Missing	0

Distributions Herd=low-performing, Indicator=POCP kgNMVOCeq

Manure handling

Compare Distributions

Show	Distribution	AICc ^	BIC	-2*LogLikelihood
☑	Normal	-240.8728	-240.6855	-245.9637

Summary Statistics

Mean	0.0001129
Std Dev	0.0000384
Std Err Mean	1.0261e-5
Upper 95% Mean	0.0001351
Lower 95% Mean	9.0743e-5
N	14
N Missing	0

Fitted Normal Distribution

Parameter		Estimate	Std Error	Lower 95%	Upper 95%
Location	μ	0.0001129	1.0261e-5	9.0743e-5	0.0001351
Dispersion	σ	0.0000384	7.6786e-6	2.7833e-5	6.1852e-5

Measures	
-2*LogLikelihood	-245.9637
AICc	-240.8728
BIC	-240.6855

Goodness-of-Fit Test

	W	Prob<W
Shapiro-Wilk	0.9518087	0.5891

	A²	Simulated p-Value
Anderson-Darling	0.2696198	0.6580

Note: Ho = The data is from the Normal distribution. Small p-values reject Ho.

Distributions Herd=mid-performing, Indicator=CC kgCO2eq

Manure handling

Compare Distributions

Show	Distribution	AICc ^	BIC	-2*LogLikelihood
☑	Normal	-30.94145	-28.84977	-35.44145

Summary Statistics

Mean	0.4460137
Std Dev	0.1278697
Std Err Mean	0.0246085
Upper 95% Mean	0.4965973
Lower 95% Mean	0.3954301
N	27
N Missing	0

Fitted Normal Distribution

Parameter		Estimate	Std Error	Lower 95%	Upper 95%
Location	μ	0.4460137	0.0246085	0.3954301	0.4965973
Dispersion	σ	0.1278697	0.0179053	0.1006995	0.1752367

Measures	
-2*LogLikelihood	-35.44145
AICc	-30.94145
BIC	-28.84977

Goodness-of-Fit Test

	W	Prob<W
Shapiro-Wilk	0.9629842	0.4311

	A²	Simulated p-Value
Anderson-Darling	0.3513178	0.4552

Note: Ho = The data is from the Normal distribution. Small p-values reject Ho.

Distributions Herd=mid-performing, Indicator=CC-biogenic kgCO2eq

Manure handling

Compare Distributions

Show	Distribution	AICc ^	BIC	-2*LogLikelihood
☑	Normal	-30.66394	-28.57227	-35.16394

Summary Statistics

Mean	0.387355
Std Dev	0.1285286
Std Err Mean	0.0247353
Upper 95% Mean	0.4381992
Lower 95% Mean	0.3365108
N	27
N Missing	0

Fitted Normal Distribution

Parameter		Estimate	Std Error	Lower 95%	Upper 95%
Location	μ	0.387355	0.0247353	0.3365108	0.4381992
Dispersion	σ	0.1285286	0.0179976	0.1012184	0.1761396

Measures	
-2*LogLikelihood	-35.16394
AICc	-30.66394
BIC	-28.57227

Goodness-of-Fit Test

	W	Prob<W
Shapiro-Wilk	0.9637055	0.4470

	A²	Simulated p-Value
Anderson-Darling	0.3505813	0.4292

Note: Ho = The data is from the Normal distribution. Small p-values reject Ho.

Distributions Herd=mid-performing, Indicator=CC-fossil kgCO2eq

Manure handling

Compare Distributions

Show	Distribution	AICc ^	BIC	-2*LogLikelihood
☑	Normal	-209.0801	-206.9884	-213.5801

Summary Statistics

Mean	0.0586587
Std Dev	0.0047216
Std Err Mean	0.0009087
Upper 95% Mean	0.0605265
Lower 95% Mean	0.0567909
N	27
N Missing	0

Fitted Normal Distribution

Parameter		Estimate	Std Error	Lower 95%	Upper 95%
Location	μ	0.0586587	0.0009087	0.0567909	0.0605265
Dispersion	σ	0.0047216	0.0006612	0.0037183	0.0064706

Measures	
-2*LogLikelihood	-213.5801
AICc	-209.0801
BIC	-206.9884

Goodness-of-Fit Test

	W	Prob<W
Shapiro-Wilk	0.9266395	0.0572

	A²	Simulated p-Value
Anderson-Darling	0.7259687	0.0540

Note: Ho = The data is from the Normal distribution. Small p-values reject Ho.

Distributions Herd=mid-performing, Indicator=CC-LTU kgCO2eq

Manure handling

Summary Statistics

Mean	0
Std Dev	0
Std Err Mean	0
Upper 95% Mean	0
Lower 95% Mean	0
N	27
N Missing	0

Distributions Herd=mid-performing, Indicator=POCP kgNMVOCeq

Manure handling

Compare Distributions

Show	Distribution	AICc ^	BIC	-2*LogLikelihood
☑	Normal	-469.2293	-467.1376	-473.7293

Summary Statistics

Mean	0.0001151
Std Dev	3.8181e-5
Std Err Mean	7.3478e-6
Upper 95% Mean	0.0001302
Lower 95% Mean	0.0001
N	27
N Missing	0

Fitted Normal Distribution

Parameter		Estimate	Std Error	Lower 95%	Upper 95%
Location	μ	0.0001151	7.3478e-6	0.0001	0.0001302
Dispersion	σ	3.8181e-5	5.3463e-6	3.0068e-5	5.2324e-5

Measures	
-2*LogLikelihood	-473.7293
AICc	-469.2293
BIC	-467.1376

Goodness-of-Fit Test

	W	Prob<W
Shapiro-Wilk	0.9637055	0.4470

	A²	Simulated p-Value
Anderson-Darling	0.3505812	0.4452

Note: Ho = The data is from the Normal distribution. Small p-values reject Ho.
