# Supplementary material for: Climate Change and Photochemical Ozone Creation Potential Impact Indicators of Cow Milk: A Comparison of Different Scenarios for a Diet Assessment
Source: Animals (Basel). 2024 Jun 7;14(12):1725. doi: 10.3390/ani14121725 (PMC11201073; doi:10.3390/ani14121725)
Supplement: Supplementary file 1 [file animals-14-01725-s001.zip › animals-3004812-supplementary/Table 3/Distribution of Total.pdf]

Distributions Herd=high-performing, Indicator=CC kgCO2eq

Total

Compare Distributions

Show	Distribution		AICc ^	BIC	-2*LogLikelihood
☑	Normal		10.220905	10.408111	5.1299959

Summary Statistics

Mean	1.6301349
Std Dev	0.3011912
Std Err Mean	0.0804967
Upper 95% Mean	1.8040375
Lower 95% Mean	1.4562323
N	14
N Missing	0

Fitted Normal Distribution

Parameter		Estimate	Std Error	Lower 95%	Upper 95%
Location	μ	1.6301349	0.0804967	1.4562323	1.8040375
Dispersion	σ	0.3011912	0.0602382	0.2183497	0.4852317

Measures	
-2*LogLikelihood	5.1299959
AICc	10.220905
BIC	10.408111

Goodness-of-Fit Test

	W	Prob<W
Shapiro-Wilk	0.9556648	0.6515

	A²	Simulated p-Value
Anderson-Darling	0.284504	0.6144

Note: Ho = The data is from the Normal distribution. Small p-values reject Ho.

Distributions Herd=high-performing, Indicator=CC-biogenic kgCO2eq

Total

Compare Distributions

Show	Distribution		AICc ^	BIC	-2*LogLikelihood
☑	Normal		-2.663031	-2.475826	-7.75394

Summary Statistics

Mean	0.9707169
Std Dev	0.1901103
Std Err Mean	0.0508091
Upper 95% Mean	1.0804834
Lower 95% Mean	0.8609505
N	14
N Missing	0

Fitted Normal Distribution

Parameter		Estimate	Std Error	Lower 95%	Upper 95%
Location	μ	0.9707169	0.0508091	0.8609505	1.0804834
Dispersion	σ	0.1901103	0.0380221	0.1378212	0.3062758

Measures	
-2*LogLikelihood	-7.75394
AICc	-2.663031
BIC	-2.475826

Goodness-of-Fit Test

	W	Prob<W
Shapiro-Wilk	0.9455414	0.4939

	A²	Simulated p-Value
Anderson-Darling	0.2784064	0.6304

Note: Ho = The data is from the Normal distribution. Small p-values reject Ho.

Distributions Herd=high-performing, Indicator=CC-fossil kgCO2eq

Total

Compare Distributions

Show	Distribution		AICc ^	BIC	-2*LogLikelihood
☑	Normal		-17.51379	-17.32659	-22.6047

Summary Statistics

Mean	0.4199913
Std Dev	0.1118569
Std Err Mean	0.029895
Upper 95% Mean	0.4845756
Lower 95% Mean	0.3554071
N	14
N Missing	0

Fitted Normal Distribution

Parameter		Estimate	Std Error	Lower 95%	Upper 95%
Location	μ	0.4199913	0.029895	0.3554071	0.4845756
Dispersion	σ	0.1118569	0.0223714	0.0810911	0.1802062

Measures	
-2*LogLikelihood	-22.6047
AICc	-17.51379
BIC	-17.32659

Goodness-of-Fit Test

	W	Prob<W
Shapiro-Wilk	0.9535914	0.6177

	A²	Simulated p-Value
Anderson-Darling	0.2322077	0.7652

Note: Ho = The data is from the Normal distribution. Small p-values reject Ho.

Distributions Herd=high-performing, Indicator=CC-LTU kgCO2eq

Total

Compare Distributions

Show	Distribution		AICc ^	BIC	-2*LogLikelihood
☑	Normal		-20.60148	-20.41428	-25.69239

Summary Statistics

Mean	0.2394267
Std Dev	0.1001777
Std Err Mean	0.0267736
Upper 95% Mean	0.2972675
Lower 95% Mean	0.1815858
N	14
N Missing	0

Fitted Normal Distribution

Parameter		Estimate	Std Error	Lower 95%	Upper 95%
Location	μ	0.2394267	0.0267736	0.1815858	0.2972675
Dispersion	σ	0.1001777	0.0200355	0.0726242	0.1613905

Measures	
-2*LogLikelihood	-25.69239
AICc	-20.60148
BIC	-20.41428

Goodness-of-Fit Test

	W	Prob<W
Shapiro-Wilk	0.8674413	0.0386*

	A²	Simulated p-Value
Anderson-Darling	0.6099238	0.0852

Note: Ho = The data is from the Normal distribution. Small p-values reject Ho.

Distributions Herd=high-performing, Indicator=POCP kgNMVOCeq

Total

Compare Distributions

Show	Distribution		AICc ^	BIC	-2*LogLikelihood
☑	Normal		-140.178	-139.9907	-145.2689

Summary Statistics

Mean	0.0068843
Std Dev	0.0013998
Std Err Mean	0.0003741
Upper 95% Mean	0.0076926
Lower 95% Mean	0.0060761
N	14
N Missing	0

Fitted Normal Distribution

Parameter		Estimate	Std Error	Lower 95%	Upper 95%
Location	μ	0.0068843	0.0003741	0.0060761	0.0076926
Dispersion	σ	0.0013998	0.00028	0.0010148	0.0022552

Measures	
-2*LogLikelihood	-145.2689
AICc	-140.178
BIC	-139.9907

Goodness-of-Fit Test

	W	Prob<W
Shapiro-Wilk	0.8806532	0.0594

	A²	Simulated p-Value
Anderson-Darling	0.7527843	0.0388*

Note: Ho = The data is from the Normal distribution. Small p-values reject Ho.

Distributions Herd=low-performing, Indicator=CC kgCO2eq

Total

Compare Distributions

Show	Distribution		AICc ^	BIC	-2*LogLikelihood
☑	Normal		9.4489471	9.6361527	4.358038

Summary Statistics

Mean	2.1734597
Std Dev	0.2930008
Std Err Mean	0.0783078
Upper 95% Mean	2.3426333
Lower 95% Mean	2.004286
N	14
N Missing	0

Fitted Normal Distribution

Parameter		Estimate	Std Error	Lower 95%	Upper 95%
Location	μ	2.1734597	0.0783078	2.004286	2.3426333
Dispersion	σ	0.2930008	0.0586002	0.2124121	0.4720367

Measures	
-2*LogLikelihood	4.358038
AICc	9.4489471
BIC	9.6361527

Goodness-of-Fit Test

	W	Prob<W
Shapiro-Wilk	0.9844299	0.9928

	A²	Simulated p-Value
Anderson-Darling	0.1447488	0.9752

Note: Ho = The data is from the Normal distribution. Small p-values reject Ho.

Distributions Herd=low-performing, Indicator=CC-biogenic kgCO2eq

Total

Compare Distributions

Show	Distribution		AICc ^	BIC	-2*LogLikelihood
☑	Normal		-7.410732	-7.223526	-12.50164

Summary Statistics

Mean	1.2174477
Std Dev	0.1604599
Std Err Mean	0.0428847
Upper 95% Mean	1.3100945
Lower 95% Mean	1.1248009
N	14
N Missing	0

Fitted Normal Distribution

Parameter		Estimate	Std Error	Lower 95%	Upper 95%
Location	μ	1.2174477	0.0428847	1.1248009	1.3100945
Dispersion	σ	0.1604599	0.032092	0.116326	0.2585077

Measures	
-2*LogLikelihood	-12.50164
AICc	-7.410732
BIC	-7.223526

Goodness-of-Fit Test

	W	Prob<W
Shapiro-Wilk	0.9583774	0.6963

	A²	Simulated p-Value
Anderson-Darling	0.2377392	0.7452

Note: Ho = The data is from the Normal distribution. Small p-values reject Ho.

Distributions Herd=low-performing, Indicator=CC-fossil kgCO2eq

Total

Compare Distributions

Show	Distribution		AICc ^	BIC	-2*LogLikelihood
☑	Normal		-18.29261	-18.10541	-23.38352

Summary Statistics

Mean	0.5902661
Std Dev	0.1087885
Std Err Mean	0.0290749
Upper 95% Mean	0.6530787
Lower 95% Mean	0.5274535
N	14
N Missing	0

Fitted Normal Distribution

Parameter		Estimate	Std Error	Lower 95%	Upper 95%
Location	μ	0.5902661	0.0290749	0.5274535	0.6530787
Dispersion	σ	0.1087885	0.0217577	0.0788666	0.1752628

Measures	
-2*LogLikelihood	-23.38352
AICc	-18.29261
BIC	-18.10541

Goodness-of-Fit Test

	W	Prob<W
Shapiro-Wilk	0.9555965	0.6504

	A²	Simulated p-Value
Anderson-Darling	0.282512	0.5936

Note: Ho = The data is from the Normal distribution. Small p-values reject Ho.

Distributions Herd=low-performing, Indicator=CC-LTU kgCO2eq

Total

Compare Distributions

Show	Distribution		AICc ^	BIC	-2*LogLikelihood
☑	Normal		-13.23611	-13.0489	-18.32701

Summary Statistics

Mean	0.3657458
Std Dev	0.1303202
Std Err Mean	0.0348295
Upper 95% Mean	0.4409905
Lower 95% Mean	0.2905012
N	14
N Missing	0

Fitted Normal Distribution

Parameter		Estimate	Std Error	Lower 95%	Upper 95%
Location	μ	0.3657458	0.0348295	0.2905012	0.4409905
Dispersion	σ	0.1303202	0.026064	0.0944762	0.2099514

Measures	
-2*LogLikelihood	-18.32701
AICc	-13.23611
BIC	-13.0489

Goodness-of-Fit Test

	W	Prob<W
Shapiro-Wilk	0.920973	0.2271

	A²	Simulated p-Value
Anderson-Darling	0.4044932	0.3188

Note: Ho = The data is from the Normal distribution. Small p-values reject Ho.

Distributions Herd=low-performing, Indicator=POCP kgNMVOCeq

Total

Compare Distributions

Show	Distribution		AICc ^	BIC	-2*LogLikelihood
☑	Normal		-135.7412	-135.5539	-140.8321

Summary Statistics

Mean	0.011159
Std Dev	0.0016402
Std Err Mean	0.0004384
Upper 95% Mean	0.0121061
Lower 95% Mean	0.010212
N	14
N Missing	0

Fitted Normal Distribution

Parameter		Estimate	Std Error	Lower 95%	Upper 95%
Location	μ	0.011159	0.0004384	0.010212	0.0121061
Dispersion	σ	0.0016402	0.000328	0.0011891	0.0026424

Measures	
-2*LogLikelihood	-140.8321
AICc	-135.7412
BIC	-135.5539

Goodness-of-Fit Test

	W	Prob<W
Shapiro-Wilk	0.9668681	0.8322

	A²	Simulated p-Value
Anderson-Darling	0.1972956	0.8728

Note: Ho = The data is from the Normal distribution. Small p-values reject Ho.

Distributions Herd=mid-performing, Indicator=CC kgCO2eq

Total

Compare Distributions

Show	Distribution		AICc ^	BIC	-2*LogLikelihood
☑	Normal		-9.838725	-7.747051	-14.33872

Summary Statistics

Mean	1.9298081
Std Dev	0.1890106
Std Err Mean	0.0363751
Upper 95% Mean	2.0045783
Lower 95% Mean	1.855038
N	27
N Missing	0

Fitted Normal Distribution

Parameter		Estimate	Std Error	Lower 95%	Upper 95%
Location	μ	1.9298081	0.0363751	1.855038	2.0045783
Dispersion	σ	0.1890106	0.0264668	0.148849	0.2590262

Measures	
-2*LogLikelihood	-14.33872
AICc	-9.838725
BIC	-7.747051

Goodness-of-Fit Test

	W	Prob<W
Shapiro-Wilk	0.9722953	0.6633

	A²	Simulated p-Value
Anderson-Darling	0.2346675	0.7832

Note: Ho = The data is from the Normal distribution. Small p-values reject Ho.

Distributions Herd=mid-performing, Indicator=CC-biogenic kgCO2eq

Total

Compare Distributions

Show	Distribution		AICc ^	BIC	-2*LogLikelihood
☑	Normal		-25.81635	-23.72468	-30.31635

Summary Statistics

Mean	1.1090927
Std Dev	0.1406003
Std Err Mean	0.0270585
Upper 95% Mean	1.1647123
Lower 95% Mean	1.053473
N	27
N Missing	0

Fitted Normal Distribution

Parameter		Estimate	Std Error	Lower 95%	Upper 95%
Location	μ	1.1090927	0.0270585	1.053473	1.1647123
Dispersion	σ	0.1406003	0.019688	0.1107251	0.1926831

Measures	
-2*LogLikelihood	-30.31635
AICc	-25.81635
BIC	-23.72468

Goodness-of-Fit Test

	W	Prob<W
Shapiro-Wilk	0.9649696	0.4759

	A²	Simulated p-Value
Anderson-Darling	0.2813962	0.6224

Note: Ho = The data is from the Normal distribution. Small p-values reject Ho.

Distributions Herd=mid-performing, Indicator=CC-fossil kgCO2eq

Total

Compare Distributions

Show	Distribution		AICc ^	BIC	-2*LogLikelihood
☑	Normal		-29.91154	-27.81987	-34.41154

Summary Statistics

Mean	0.5312815
Std Dev	0.1303319
Std Err Mean	0.0250824
Upper 95% Mean	0.5828391
Lower 95% Mean	0.4797239
N	27
N Missing	0

Fitted Normal Distribution

Parameter		Estimate	Std Error	Lower 95%	Upper 95%
Location	μ	0.5312815	0.0250824	0.4797239	0.5828391
Dispersion	σ	0.1303319	0.0182501	0.1026385	0.178611

Measures	
-2*LogLikelihood	-34.41154
AICc	-29.91154
BIC	-27.81987

Goodness-of-Fit Test

	W	Prob<W
Shapiro-Wilk	0.8911203	0.0084*

	A²	Simulated p-Value
Anderson-Darling	0.8444637	0.0264*

Note: Ho = The data is from the Normal distribution. Small p-values reject Ho.

Distributions Herd=mid-performing, Indicator=CC-LTU kgCO2eq

Total

Compare Distributions

Show	Distribution		AICc ^	BIC	-2*LogLikelihood
☑	Normal		-43.88166	-41.78999	-48.38166

Summary Statistics

Mean	0.289434
Std Dev	0.1006228
Std Err Mean	0.0193649
Upper 95% Mean	0.329239
Lower 95% Mean	0.249629
N	27
N Missing	0

Fitted Normal Distribution

Parameter		Estimate	Std Error	Lower 95%	Upper 95%
Location	μ	0.289434	0.0193649	0.249629	0.329239
Dispersion	σ	0.1006228	0.01409	0.0792421	0.1378966

Measures	
-2*LogLikelihood	-48.38166
AICc	-43.88166
BIC	-41.78999

Goodness-of-Fit Test

	W	Prob<W
Shapiro-Wilk	0.9034982	0.0161*

	A²	Simulated p-Value
Anderson-Darling	0.9762854	0.0132*

Note: Ho = The data is from the Normal distribution. Small p-values reject Ho.

Distributions Herd=mid-performing, Indicator=POCP kgNMVOCeq

Total

Compare Distributions

Show	Distribution		AICc ^	BIC	-2*LogLikelihood
☑	Normal		-304.6002	-302.5085	-309.1002

Summary Statistics

Mean	0.0086457
Std Dev	0.0008051
Std Err Mean	0.0001549
Upper 95% Mean	0.0089642
Lower 95% Mean	0.0083272
N	27
N Missing	0

Fitted Normal Distribution

Parameter		Estimate	Std Error	Lower 95%	Upper 95%
Location	μ	0.0086457	0.0001549	0.0083272	0.0089642
Dispersion	σ	0.0008051	0.0001127	0.0006341	0.0011034

Measures	
-2*LogLikelihood	-309.1002
AICc	-304.6002
BIC	-302.5085

Goodness-of-Fit Test

	W	Prob<W
Shapiro-Wilk	0.9585054	0.3415

	A²	Simulated p-Value
Anderson-Darling	0.3574549	0.4264

Note: Ho = The data is from the Normal distribution. Small p-values reject Ho.
