# Supplementary material for: Climate Change and Photochemical Ozone Creation Potential Impact Indicators of Cow Milk: A Comparison of Different Scenarios for a Diet Assessment
Source: Animals (Basel). 2024 Jun 7;14(12):1725. doi: 10.3390/ani14121725 (PMC11201073; doi:10.3390/ani14121725)

Distributions Herd=high-performing, Indicator=CC kgCO2eq

Enteric fermentation_Difference_NRC_IPCC

Compare Distributions

Show	Distribution	AICc ^	BIC	-2*LogLikelihood
☑	Normal	-30.35485	-30.16764	-35.44576

Summary Statistics

Mean	-0.100449
Std Dev	0.0707117
Std Err Mean	0.0188985
Upper 95% Mean	-0.059622
Lower 95% Mean	-0.141277
N	14
N Missing	0

Fitted Normal Distribution

Parameter		Estimate	Std Error	Lower 95%	Upper 95%
Location	μ	-0.100449	0.0188985	-0.141277	-0.059622
Dispersion	σ	0.0707117	0.0141423	0.0512627	0.1139196

Measures	
-2*LogLikelihood	-35.44576
AICc	-30.35485
BIC	-30.16764

Goodness-of-Fit Test

	W	Prob<W
Shapiro-Wilk	0.8628275	0.0333*

	A²	Simulated p-Value
Anderson-Darling	0.8170529	0.0232*

Note: Ho = The data is from the Normal distribution. Small p-values reject Ho.

Test Mean

Hypothesized Value	0
Actual Estimate	-0.1004
DF	13
Std Dev	0.07071

	t Test	Signed-Rank
Test Statistic	-5.3152	-52.5000
Prob > |t|	0.0001*	0.0001*
Prob > t	0.9999	0.9999
Prob < t	<.0001*	<.0001*

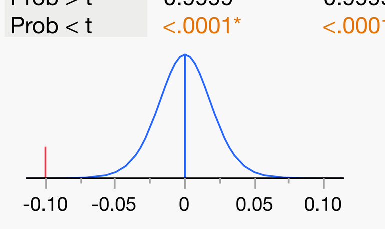

Distributions Herd=high-performing, Indicator=CC-biogenic kgCO2eq

Enteric fermentation_Difference_NRC_IPCC

Compare Distributions

Show	Distribution	AICc ^	BIC	-2*LogLikelihood
☑	Normal	-30.35485	-30.16764	-35.44576

Summary Statistics

Mean	-0.100449
Std Dev	0.0707117
Std Err Mean	0.0188985
Upper 95% Mean	-0.059622
Lower 95% Mean	-0.141277
N	14
N Missing	0

Fitted Normal Distribution

Parameter		Estimate	Std Error	Lower 95%	Upper 95%
Location	μ	-0.100449	0.0188985	-0.141277	-0.059622
Dispersion	σ	0.0707117	0.0141423	0.0512627	0.1139196

Measures	
-2*LogLikelihood	-35.44576
AICc	-30.35485
BIC	-30.16764

Goodness-of-Fit Test

	W	Prob<W
Shapiro-Wilk	0.8628275	0.0333*

	A²	Simulated p-Value
Anderson-Darling	0.8170529	0.0264*

Note: Ho = The data is from the Normal distribution. Small p-values reject Ho.

Test Mean

Hypothesized Value	0
Actual Estimate	-0.1004
DF	13
Std Dev	0.07071

	t Test	Signed-Rank
Test Statistic	-5.3152	-52.5000
Prob > |t|	0.0001*	0.0001*
Prob > t	0.9999	0.9999
Prob < t	<.0001*	<.0001*

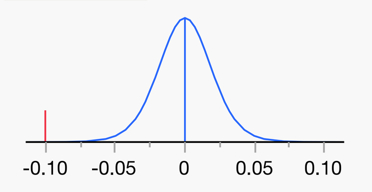

Distributions Herd=high-performing, Indicator=CC-fossil kgCO2eq

Enteric fermentation_Difference_NRC_IPCC

Summary Statistics

Mean	0
Std Dev	0
Std Err Mean	0
Upper 95% Mean	0
Lower 95% Mean	0
N	14
N Missing	0

Test Mean

Hypothesized Value	0
Actual Estimate	0
DF	13
Std Dev	0

	t Test	Signed-Rank
Test Statistic	.	0.0000
Prob > |t|	.	1.0000
Prob > t	.	0.5000
Prob < t	.	0.5000

Distributions Herd=high-performing, Indicator=CC-LTU kgCO2eq

Enteric fermentation_Difference_NRC_IPCC

Summary Statistics

Mean	0
Std Dev	0
Std Err Mean	0
Upper 95% Mean	0
Lower 95% Mean	0
N	14
N Missing	0

Test Mean

Hypothesized Value	0
Actual Estimate	0
DF	13
Std Dev	0

	t Test	Signed-Rank
Test Statistic	.	0.0000
Prob > |t|	.	1.0000
Prob > t	.	0.5000
Prob < t	.	0.5000

Distributions Herd=high-performing, Indicator=POCP kgNMVOCeq

Enteric fermentation_Difference_NRC_IPCC

Compare Distributions

Show	Distribution	AICc ^	BIC	-2*LogLikelihood
☑	Normal	-257.7591	-257.5719	-262.85

Summary Statistics

Mean	-2.984e-5
Std Dev	0.000021
Std Err Mean	5.614e-6
Upper 95% Mean	-1.771e-5
Lower 95% Mean	-0.000042
N	14
N Missing	0

Fitted Normal Distribution

Parameter		Estimate	Std Error	Lower 95%	Upper 95%
Location	μ	-2.984e-5	5.614e-6	-0.000042	-1.771e-5
Dispersion	σ	0.000021	4.2011e-6	1.5228e-5	3.3841e-5

Measures	
-2*LogLikelihood	-262.85
AICc	-257.7591
BIC	-257.5719

Goodness-of-Fit Test

	W	Prob<W
Shapiro-Wilk	0.8628275	0.0333*

	A²	Simulated p-Value
Anderson-Darling	0.8170529	0.0252*

Note: Ho = The data is from the Normal distribution. Small p-values reject Ho.

Test Mean

Hypothesized Value	0
Actual Estimate	-3e-5
DF	13
Std Dev	2.1e-5

	t Test	Signed-Rank
Test Statistic	-5.3152	-52.5000
Prob > |t|	0.0001*	0.0001*
Prob > t	0.9999	0.9999
Prob < t	<.0001*	<.0001*

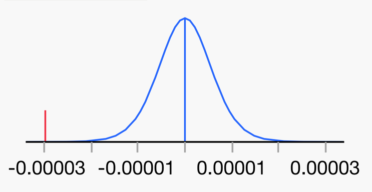

Distributions Herd=low-performing, Indicator=CC kgCO2eq

Enteric fermentation_Difference_NRC_IPCC

Compare Distributions

Show	Distribution	AICc ^	BIC	-2*LogLikelihood
☑	Normal	-17.78353	-17.59632	-22.87444

Summary Statistics

Mean	0.0147056
Std Dev	0.1107845
Std Err Mean	0.0296084
Upper 95% Mean	0.0786707
Lower 95% Mean	-0.049259
N	14
N Missing	0

Fitted Normal Distribution

Parameter		Estimate	Std Error	Lower 95%	Upper 95%
Location	μ	0.0147056	0.0296084	-0.049259	0.0786707
Dispersion	σ	0.1107845	0.0221569	0.0803137	0.1784785

Measures	
-2*LogLikelihood	-22.87444
AICc	-17.78353
BIC	-17.59632

Goodness-of-Fit Test

	W	Prob<W
Shapiro-Wilk	0.9474348	0.5216

	A²	Simulated p-Value
Anderson-Darling	0.3725699	0.3904

Note: Ho = The data is from the Normal distribution. Small p-values reject Ho.

Test Mean

Hypothesized Value	0
Actual Estimate	0.01471
DF	13
Std Dev	0.11078

	t Test	Signed-Rank
Test Statistic	0.4967	8.5000
Prob > |t|	0.6277	0.6257
Prob > t	0.3139	0.3129
Prob < t	0.6861	0.6871

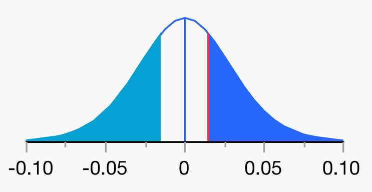

Distributions Herd=low-performing, Indicator=CC-biogenic kgCO2eq

Enteric fermentation_Difference_NRC_IPCC

Compare Distributions

Show	Distribution	AICc ^	BIC	-2*LogLikelihood
☑	Normal	-17.78353	-17.59632	-22.87444

Summary Statistics

Mean	0.0147056
Std Dev	0.1107845
Std Err Mean	0.0296084
Upper 95% Mean	0.0786707
Lower 95% Mean	-0.049259
N	14
N Missing	0

Fitted Normal Distribution

Parameter		Estimate	Std Error	Lower 95%	Upper 95%
Location	μ	0.0147056	0.0296084	-0.049259	0.0786707
Dispersion	σ	0.1107845	0.0221569	0.0803137	0.1784785

Measures	
-2*LogLikelihood	-22.87444
AICc	-17.78353
BIC	-17.59632

Goodness-of-Fit Test

	W	Prob<W
Shapiro-Wilk	0.9474348	0.5216

	A²	Simulated p-Value
Anderson-Darling	0.3725699	0.3968

Note: Ho = The data is from the Normal distribution. Small p-values reject Ho.

Test Mean

Hypothesized Value	0
Actual Estimate	0.01471
DF	13
Std Dev	0.11078

	t Test	Signed-Rank
Test Statistic	0.4967	8.5000
Prob > |t|	0.6277	0.6257
Prob > t	0.3139	0.3129
Prob < t	0.6861	0.6871

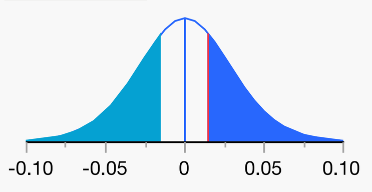

Distributions Herd=low-performing, Indicator=CC-fossil kgCO2eq

Enteric fermentation_Difference_NRC_IPCC

Summary Statistics

Mean	0
Std Dev	0
Std Err Mean	0
Upper 95% Mean	0
Lower 95% Mean	0
N	14
N Missing	0

Test Mean

Hypothesized Value	0
Actual Estimate	0
DF	13
Std Dev	0

	t Test	Signed-Rank
Test Statistic	.	0.0000
Prob > |t|	.	1.0000
Prob > t	.	0.5000
Prob < t	.	0.5000

Distributions Herd=low-performing, Indicator=CC-LTU kgCO2eq

Enteric fermentation_Difference_NRC_IPCC

Summary Statistics

Mean	0
Std Dev	0
Std Err Mean	0
Upper 95% Mean	0
Lower 95% Mean	0
N	14
N Missing	0

Test Mean

Hypothesized Value	0
Actual Estimate	0
DF	13
Std Dev	0

	t Test	Signed-Rank
Test Statistic	.	0.0000
Prob > |t|	.	1.0000
Prob > t	.	0.5000
Prob < t	.	0.5000

Distributions Herd=low-performing, Indicator=POCP kgNMVOCeq

Enteric fermentation_Difference_NRC_IPCC

Compare Distributions

Show	Distribution	AICc ^	BIC	-2*LogLikelihood
☑	Normal	-245.1878	-245.0006	-250.2787

Summary Statistics

Mean	4.3684e-6
Std Dev	0.0000329
Std Err Mean	8.7954e-6
Upper 95% Mean	2.337e-5
Lower 95% Mean	-1.463e-5
N	14
N Missing	0

Fitted Normal Distribution

Parameter		Estimate	Std Error	Lower 95%	Upper 95%
Location	μ	4.3684e-6	8.7954e-6	-1.463e-5	2.337e-5
Dispersion	σ	0.0000329	6.5819e-6	2.3858e-5	5.3019e-5

Measures	
-2*LogLikelihood	-250.2787
AICc	-245.1878
BIC	-245.0006

Goodness-of-Fit Test

	W	Prob<W
Shapiro-Wilk	0.9474347	0.5216

	A²	Simulated p-Value
Anderson-Darling	0.3725702	0.3904

Note: Ho = The data is from the Normal distribution. Small p-values reject Ho.

Test Mean

Hypothesized Value	0
Actual Estimate	4.37e-6
DF	13
Std Dev	3.29e-5

	t Test	Signed-Rank
Test Statistic	0.4967	8.5000
Prob > |t|	0.6277	0.6257
Prob > t	0.3139	0.3129
Prob < t	0.6861	0.6871

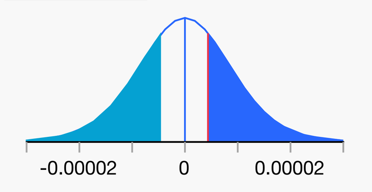

Distributions Herd=mid-performing, Indicator=CC kgCO2eq

Enteric fermentation_Difference_NRC_IPCC

Compare Distributions

Show	Distribution	AICc ^	BIC	-2*LogLikelihood
☑	Normal	-71.6533	-69.56163	-76.1533

Summary Statistics

Mean	-0.043537
Std Dev	0.0601649
Std Err Mean	0.0115787
Upper 95% Mean	-0.019736
Lower 95% Mean	-0.067337
N	27
N Missing	0

Fitted Normal Distribution

Parameter		Estimate	Std Error	Lower 95%	Upper 95%
Location	μ	-0.043537	0.0115787	-0.067337	-0.019736
Dispersion	σ	0.0601649	0.0084248	0.0473808	0.0824519

Measures	
-2*LogLikelihood	-76.1533
AICc	-71.6533
BIC	-69.56163

Goodness-of-Fit Test

	W	Prob<W
Shapiro-Wilk	0.8944813	0.0100*

	A²	Simulated p-Value
Anderson-Darling	0.862642	0.0240*

Note: Ho = The data is from the Normal distribution. Small p-values reject Ho.

Test Mean

Hypothesized Value	0
Actual Estimate	-0.0435
DF	26
Std Dev	0.06016

	t Test	Signed-Rank
Test Statistic	-3.7600	-142.000
Prob > |t|	0.0009*	0.0001*
Prob > t	0.9996	0.9999
Prob < t	0.0004*	<.0001*

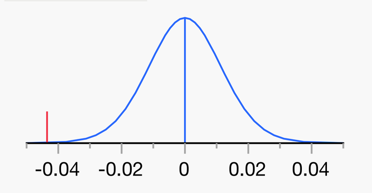

Distributions Herd=mid-performing, Indicator=CC-biogenic kgCO2eq

Enteric fermentation_Difference_NRC_IPCC

Compare Distributions

Show	Distribution	AICc ^	BIC	-2*LogLikelihood
☑	Normal	-71.6533	-69.56163	-76.1533

Summary Statistics

Mean	-0.043537
Std Dev	0.0601649
Std Err Mean	0.0115787
Upper 95% Mean	-0.019736
Lower 95% Mean	-0.067337
N	27
N Missing	0

Fitted Normal Distribution

Parameter		Estimate	Std Error	Lower 95%	Upper 95%
Location	μ	-0.043537	0.0115787	-0.067337	-0.019736
Dispersion	σ	0.0601649	0.0084248	0.0473808	0.0824519

Measures	
-2*LogLikelihood	-76.1533
AICc	-71.6533
BIC	-69.56163

Goodness-of-Fit Test

	W	Prob<W
Shapiro-Wilk	0.8944813	0.0100*

	A²	Simulated p-Value
Anderson-Darling	0.862642	0.0272*

Note: Ho = The data is from the Normal distribution. Small p-values reject Ho.

Test Mean

Hypothesized Value	0
Actual Estimate	-0.0435
DF	26
Std Dev	0.06016

	t Test	Signed-Rank
Test Statistic	-3.7600	-142.000
Prob > |t|	0.0009*	0.0001*
Prob > t	0.9996	0.9999
Prob < t	0.0004*	<.0001*

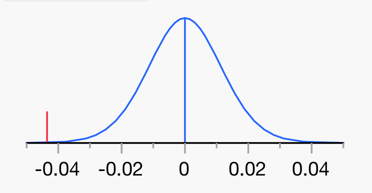

Distributions Herd=mid-performing, Indicator=CC-fossil kgCO2eq

Enteric fermentation_Difference_NRC_IPCC

Summary Statistics

Mean	0
Std Dev	0
Std Err Mean	0
Upper 95% Mean	0
Lower 95% Mean	0
N	27
N Missing	0

Test Mean

Hypothesized Value	0
Actual Estimate	0
DF	26
Std Dev	0

	t Test	Signed-Rank
Test Statistic	.	0.0000
Prob > |t|	.	1.0000
Prob > t	.	0.5000
Prob < t	.	0.5000

Distributions Herd=mid-performing, Indicator=CC-LTU kgCO2eq

Enteric fermentation_Difference_NRC_IPCC

Summary Statistics

Mean	0
Std Dev	0
Std Err Mean	0
Upper 95% Mean	0
Lower 95% Mean	0
N	27
N Missing	0

Test Mean

Hypothesized Value	0
Actual Estimate	0
DF	26
Std Dev	0

	t Test	Signed-Rank
Test Statistic	.	0.0000
Prob > |t|	.	1.0000
Prob > t	.	0.5000
Prob < t	.	0.5000

Distributions Herd=mid-performing, Indicator=POCP kgNMVOCeq

Enteric fermentation_Difference_NRC_IPCC

Compare Distributions

Show	Distribution	AICc ^	BIC	-2*LogLikelihood
☑	Normal	-510.2186	-508.127	-514.7186

Summary Statistics

Mean	-0.000013
Std Dev	1.7873e-5
Std Err Mean	3.4396e-6
Upper 95% Mean	-5.863e-6
Lower 95% Mean	-0.00002
N	27
N Missing	0

Fitted Normal Distribution

Parameter		Estimate	Std Error	Lower 95%	Upper 95%
Location	μ	-0.000013	3.4396e-6	-0.00002	-5.863e-6
Dispersion	σ	1.7873e-5	2.5027e-6	1.4075e-5	0.0000245

Measures	
-2*LogLikelihood	-514.7186
AICc	-510.2186
BIC	-508.127

Goodness-of-Fit Test

	W	Prob<W
Shapiro-Wilk	0.8944812	0.0100*

	A²	Simulated p-Value
Anderson-Darling	0.8626423	0.0244*

Note: Ho = The data is from the Normal distribution. Small p-values reject Ho.

Test Mean

Hypothesized Value	0
Actual Estimate	-1.3e-5
DF	26
Std Dev	1.79e-5

	t Test	Signed-Rank
Test Statistic	-3.7600	-142.000
Prob > |t|	0.0009*	0.0001*
Prob > t	0.9996	0.9999
Prob < t	0.0004*	<.0001*

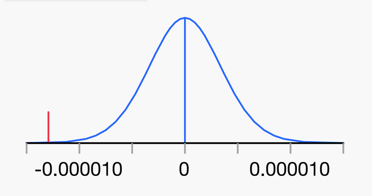

Supplement: Supplementary file 1 [file animals-14-01725-s001.zip › animals-3004812-supplementary/Table 4/Distribution and Test of Enteric fermentation_Difference_NRC_IPCC.pdf]
