# Supplementary material for: Climate Change and Photochemical Ozone Creation Potential Impact Indicators of Cow Milk: A Comparison of Different Scenarios for a Diet Assessment
Source: Animals (Basel). 2024 Jun 7;14(12):1725. doi: 10.3390/ani14121725 (PMC11201073; doi:10.3390/ani14121725)

Distributions Herd=high-performing, Indicator=CC kgCO2eq

Manure handling_Difference_NRC_IPCC

Compare Distributions

Show	Distribution		AICc ^	BIC	-2*LogLikelihood
☑	Normal		-47.47107	-47.28387	-52.56198

Summary Statistics

Mean	-0.041409
Std Dev	0.0383716
Std Err Mean	0.0102552
Upper 95% Mean	-0.019253
Lower 95% Mean	-0.063564
N	14
N Missing	0

Fitted Normal Distribution

Parameter		Estimate	Std Error	Lower 95%	Upper 95%
Location	μ	-0.041409	0.0102552	-0.063564	-0.019253
Dispersion	σ	0.0383716	0.0076743	0.0278176	0.0618183

Measures	
-2*LogLikelihood	-52.56198
AICc	-47.47107
BIC	-47.28387

Goodness-of-Fit Test

	W	Prob<W
Shapiro-Wilk	0.9163855	0.1950

	A²	Simulated p-Value
Anderson-Darling	0.4030103	0.3276

Note: Ho = The data is from the Normal distribution. Small p-values reject Ho.

Test Mean

Hypothesized Value	0
Actual Estimate	-0.0414
DF	13
Std Dev	0.03837

	t Test	Signed-Rank
Test Statistic	-4.0378	-46.5000
Prob > |t|	0.0014*	0.0017*
Prob > t	0.9993	0.9991
Prob < t	0.0007*	0.0009*

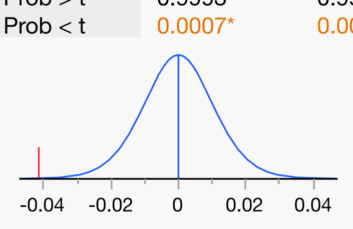

Distributions Herd=high-performing, Indicator=CC-biogenic kgCO2eq

Manure handling_Difference_NRC_IPCC

Compare Distributions

Show	Distribution		AICc ^	BIC	-2*LogLikelihood
☑	Normal		-47.47107	-47.28387	-52.56198

Summary Statistics

Mean	-0.041409
Std Dev	0.0383716
Std Err Mean	0.0102552
Upper 95% Mean	-0.019253
Lower 95% Mean	-0.063564
N	14
N Missing	0

Fitted Normal Distribution

Parameter		Estimate	Std Error	Lower 95%	Upper 95%
Location	μ	-0.041409	0.0102552	-0.063564	-0.019253
Dispersion	σ	0.0383716	0.0076743	0.0278176	0.0618183

Measures	
-2*LogLikelihood	-52.56198
AICc	-47.47107
BIC	-47.28387

Goodness-of-Fit Test

	W	Prob<W
Shapiro-Wilk	0.9163855	0.1950

	A²	Simulated p-Value
Anderson-Darling	0.4030102	0.3124

Note: Ho = The data is from the Normal distribution. Small p-values reject Ho.

Test Mean

Hypothesized Value	0
Actual Estimate	-0.0414
DF	13
Std Dev	0.03837

	t Test	Signed-Rank
Test Statistic	-4.0378	-46.5000
Prob > |t|	0.0014*	0.0017*
Prob > t	0.9993	0.9991
Prob < t	0.0007*	0.0009*

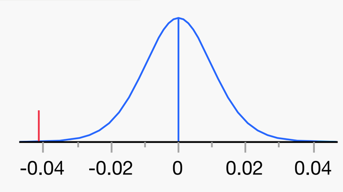

Distributions Herd=high-performing, Indicator=CC-fossil kgCO2eq

Manure handling_Difference_NRC_IPCC

Summary Statistics

Mean	0
Std Dev	0
Std Err Mean	0
Upper 95% Mean	0
Lower 95% Mean	0
N	14
N Missing	0

Test Mean

Hypothesized Value	0
Actual Estimate	0
DF	13
Std Dev	0

	t Test	Signed-Rank
Test Statistic	.	0.0000
Prob > |t|	.	1.0000
Prob > t	.	0.5000
Prob < t	.	0.5000

Distributions Herd=high-performing, Indicator=CC-LTU kgCO2eq

Manure handling_Difference_NRC_IPCC

Summary Statistics

Mean	0
Std Dev	0
Std Err Mean	0
Upper 95% Mean	0
Lower 95% Mean	0
N	14
N Missing	0

Test Mean

Hypothesized Value	0
Actual Estimate	0
DF	13
Std Dev	0

	t Test	Signed-Rank
Test Statistic	.	0.0000
Prob > |t|	.	1.0000
Prob > t	.	0.5000
Prob < t	.	0.5000

Distributions Herd=high-performing, Indicator=POCP kgNMVOCeq

Manure handling_Difference_NRC_IPCC

Compare Distributions

Show	Distribution		AICc ^	BIC	-2*LogLikelihood
☑	Normal		-274.8753	-274.6881	-279.9662

Summary Statistics

Mean	-1.23e-5
Std Dev	0.0000114
Std Err Mean	3.0464e-6
Upper 95% Mean	-5.719e-6
Lower 95% Mean	-1.888e-5
N	14
N Missing	0

Fitted Normal Distribution

Parameter		Estimate	Std Error	Lower 95%	Upper 95%
Location	μ	-1.23e-5	3.0464e-6	-1.888e-5	-5.719e-6
Dispersion	σ	0.0000114	2.2797e-6	8.2635e-6	1.8364e-5

Measures	
-2*LogLikelihood	-279.9662
AICc	-274.8753
BIC	-274.6881

Goodness-of-Fit Test

	W	Prob<W
Shapiro-Wilk	0.9163855	0.1950

	A²	Simulated p-Value
Anderson-Darling	0.4030103	0.3264

Note: Ho = The data is from the Normal distribution. Small p-values reject Ho.

Test Mean

Hypothesized Value	0
Actual Estimate	-1.2e-5
DF	13
Std Dev	1.14e-5

	t Test	Signed-Rank
Test Statistic	-4.0378	-46.5000
Prob > |t|	0.0014*	0.0017*
Prob > t	0.9993	0.9991
Prob < t	0.0007*	0.0009*

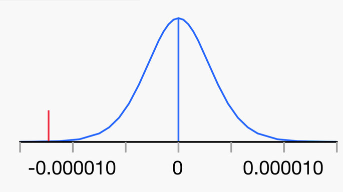

Distributions Herd=low-performing, Indicator=CC kgCO2eq

Manure handling_Difference_NRC_IPCC

Compare Distributions

Show	Distribution		AICc ^	BIC	-2*LogLikelihood
☑	Normal		-36.99685	-36.80965	-42.08776

Summary Statistics

Mean	0.0091703
Std Dev	0.055779
Std Err Mean	0.0149076
Upper 95% Mean	0.0413761
Lower 95% Mean	-0.023036
N	14
N Missing	0

Fitted Normal Distribution

Parameter		Estimate	Std Error	Lower 95%	Upper 95%
Location	μ	0.0091703	0.0149076	-0.023036	0.0413761
Dispersion	σ	0.055779	0.0111558	0.0404372	0.0898623

Measures	
-2*LogLikelihood	-42.08776
AICc	-36.99685
BIC	-36.80965

Goodness-of-Fit Test

	W	Prob<W
Shapiro-Wilk	0.9701467	0.8786

	A²	Simulated p-Value
Anderson-Darling	0.2438248	0.7452

Note: Ho = The data is from the Normal distribution. Small p-values reject Ho.

Test Mean

Hypothesized Value	0
Actual Estimate	0.00917
DF	13
Std Dev	0.05578

	t Test	Signed-Rank
Test Statistic	0.6151	8.5000
Prob > |t|	0.5491	0.6257
Prob > t	0.2745	0.3129
Prob < t	0.7255	0.6871

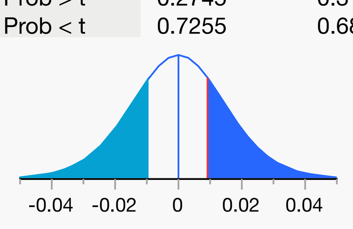

Distributions Herd=low-performing, Indicator=CC-biogenic kgCO2eq

Manure handling_Difference_NRC_IPCC

Compare Distributions

Show	Distribution		AICc ^	BIC	-2*LogLikelihood
☑	Normal		-36.99685	-36.80965	-42.08776

Summary Statistics

Mean	0.0091703
Std Dev	0.055779
Std Err Mean	0.0149076
Upper 95% Mean	0.0413761
Lower 95% Mean	-0.023036
N	14
N Missing	0

Fitted Normal Distribution

Parameter		Estimate	Std Error	Lower 95%	Upper 95%
Location	μ	0.0091703	0.0149076	-0.023036	0.0413761
Dispersion	σ	0.055779	0.0111558	0.0404372	0.0898623

Measures	
-2*LogLikelihood	-42.08776
AICc	-36.99685
BIC	-36.80965

Goodness-of-Fit Test

	W	Prob<W
Shapiro-Wilk	0.9701467	0.8786

	A²	Simulated p-Value
Anderson-Darling	0.2438249	0.7332

Note: Ho = The data is from the Normal distribution. Small p-values reject Ho.

Test Mean

Hypothesized Value	0
Actual Estimate	0.00917
DF	13
Std Dev	0.05578

	t Test	Signed-Rank
Test Statistic	0.6151	8.5000
Prob > |t|	0.5491	0.6257
Prob > t	0.2745	0.3129
Prob < t	0.7255	0.6871

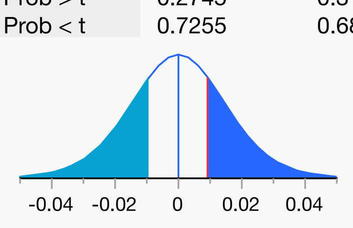

Distributions Herd=low-performing, Indicator=CC-fossil kgCO2eq

Manure handling_Difference_NRC_IPCC

Summary Statistics

Mean	0
Std Dev	0
Std Err Mean	0
Upper 95% Mean	0
Lower 95% Mean	0
N	14
N Missing	0

Test Mean

Hypothesized Value	0
Actual Estimate	0
DF	13
Std Dev	0

	t Test	Signed-Rank
Test Statistic	.	0.0000
Prob > |t|	.	1.0000
Prob > t	.	0.5000
Prob < t	.	0.5000

Distributions Herd=low-performing, Indicator=CC-LTU kgCO2eq

Manure handling_Difference_NRC_IPCC

Summary Statistics

Mean	0
Std Dev	0
Std Err Mean	0
Upper 95% Mean	0
Lower 95% Mean	0
N	14
N Missing	0

Test Mean

Hypothesized Value	0
Actual Estimate	0
DF	13
Std Dev	0

	t Test	Signed-Rank
Test Statistic	.	0.0000
Prob > |t|	.	1.0000
Prob > t	.	0.5000
Prob < t	.	0.5000

Distributions Herd=low-performing, Indicator=POCP kgNMVOCeq

Manure handling_Difference_NRC_IPCC

Compare Distributions

Show	Distribution		AICc ^	BIC	-2*LogLikelihood
☑	Normal		-264.4011	-264.2139	-269.492

Summary Statistics

Mean	2.7241e-6
Std Dev	1.657e-5
Std Err Mean	4.4284e-6
Upper 95% Mean	0.0000123
Lower 95% Mean	-6.843e-6
N	14
N Missing	0

Fitted Normal Distribution

Parameter		Estimate	Std Error	Lower 95%	Upper 95%
Location	μ	2.7241e-6	4.4284e-6	-6.843e-6	0.0000123
Dispersion	σ	1.657e-5	3.3139e-6	1.2012e-5	0.0000267

Measures	
-2*LogLikelihood	-269.492
AICc	-264.4011
BIC	-264.2139

Goodness-of-Fit Test

	W	Prob<W
Shapiro-Wilk	0.9701468	0.8786

	A²	Simulated p-Value
Anderson-Darling	0.2438247	0.7544

Note: Ho = The data is from the Normal distribution. Small p-values reject Ho.

Test Mean

Hypothesized Value	0
Actual Estimate	2.72e-6
DF	13
Std Dev	1.66e-5

	t Test	Signed-Rank
Test Statistic	0.6151	8.5000
Prob > |t|	0.5491	0.6257
Prob > t	0.2745	0.3129
Prob < t	0.7255	0.6871

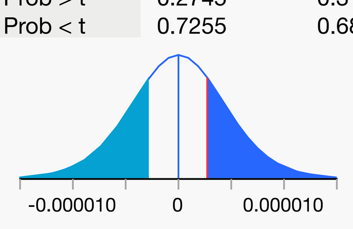

Distributions Herd=mid-performing, Indicator=CC kgCO2eq

Manure handling_Difference_NRC_IPCC

Compare Distributions

Show	Distribution		AICc ^	BIC	-2*LogLikelihood
☑	Normal		-88.56502	-86.47335	-93.06502

Summary Statistics

Mean	-0.01831
Std Dev	0.0439877
Std Err Mean	0.0084654
Upper 95% Mean	-0.000909
Lower 95% Mean	-0.035711
N	27
N Missing	0

Fitted Normal Distribution

Parameter		Estimate	Std Error	Lower 95%	Upper 95%
Location	μ	-0.01831	0.0084654	-0.035711	-0.000909
Dispersion	σ	0.0439877	0.0061595	0.034641	0.0602821

Measures	
-2*LogLikelihood	-93.06502
AICc	-88.56502
BIC	-86.47335

Goodness-of-Fit Test

	W	Prob<W
Shapiro-Wilk	0.9612109	0.3937

	A²	Simulated p-Value
Anderson-Darling	0.5180514	0.1812

Note: Ho = The data is from the Normal distribution. Small p-values reject Ho.

Test Mean

Hypothesized Value	0
Actual Estimate	-0.0183
DF	26
Std Dev	0.04399

	t Test	Signed-Rank
Test Statistic	-2.1629	-92.0000
Prob > |t|	0.0399*	0.0240*
Prob > t	0.9800	0.9880
Prob < t	0.0200*	0.0120*

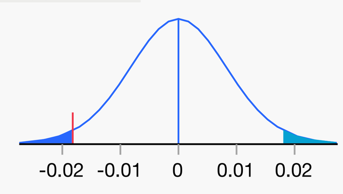

Distributions Herd=mid-performing, Indicator=CC-biogenic kgCO2eq

Manure handling_Difference_NRC_IPCC

Compare Distributions

Show	Distribution		AICc ^	BIC	-2*LogLikelihood
☑	Normal		-88.56502	-86.47335	-93.06502

Summary Statistics

Mean	-0.01831
Std Dev	0.0439877
Std Err Mean	0.0084654
Upper 95% Mean	-0.000909
Lower 95% Mean	-0.035711
N	27
N Missing	0

Fitted Normal Distribution

Parameter		Estimate	Std Error	Lower 95%	Upper 95%
Location	μ	-0.01831	0.0084654	-0.035711	-0.000909
Dispersion	σ	0.0439877	0.0061595	0.034641	0.0602821

Measures	
-2*LogLikelihood	-93.06502
AICc	-88.56502
BIC	-86.47335

Goodness-of-Fit Test

	W	Prob<W
Shapiro-Wilk	0.9612109	0.3937

	A²	Simulated p-Value
Anderson-Darling	0.5180515	0.1808

Note: Ho = The data is from the Normal distribution. Small p-values reject Ho.

Test Mean

Hypothesized Value	0
Actual Estimate	-0.0183
DF	26
Std Dev	0.04399

	t Test	Signed-Rank
Test Statistic	-2.1629	-92.0000
Prob > |t|	0.0399*	0.0240*
Prob > t	0.9800	0.9880
Prob < t	0.0200*	0.0120*

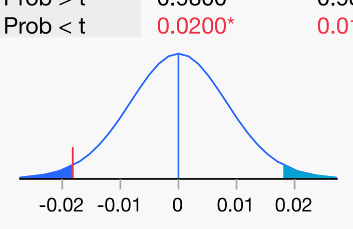

Distributions Herd=mid-performing, Indicator=CC-fossil kgCO2eq

Manure handling_Difference_NRC_IPCC

Summary Statistics

Mean	0
Std Dev	0
Std Err Mean	0
Upper 95% Mean	0
Lower 95% Mean	0
N	27
N Missing	0

Test Mean

Hypothesized Value	0
Actual Estimate	0
DF	26
Std Dev	0

	t Test	Signed-Rank
Test Statistic	.	0.0000
Prob > |t|	.	1.0000
Prob > t	.	0.5000
Prob < t	.	0.5000

Distributions Herd=mid-performing, Indicator=CC-LTU kgCO2eq

Manure handling_Difference_NRC_IPCC

Summary Statistics

Mean	0
Std Dev	0
Std Err Mean	0
Upper 95% Mean	0
Lower 95% Mean	0
N	27
N Missing	0

Test Mean

Hypothesized Value	0
Actual Estimate	0
DF	26
Std Dev	0

	t Test	Signed-Rank
Test Statistic	.	0.0000
Prob > |t|	.	1.0000
Prob > t	.	0.5000
Prob < t	.	0.5000

Distributions Herd=mid-performing, Indicator=POCP kgNMVOCeq

Manure handling_Difference_NRC_IPCC

Compare Distributions

Show	Distribution		AICc ^	BIC	-2*LogLikelihood
☑	Normal		-527.1304	-525.0387	-531.6304

Summary Statistics

Mean	-5.439e-6
Std Dev	1.3067e-5
Std Err Mean	2.5147e-6
Upper 95% Mean	-2.7e-7
Lower 95% Mean	-1.061e-5
N	27
N Missing	0

Fitted Normal Distribution

Parameter		Estimate	Std Error	Lower 95%	Upper 95%
Location	μ	-5.439e-6	2.5147e-6	-1.061e-5	-2.7e-7
Dispersion	σ	1.3067e-5	1.8297e-6	0.0000103	0.0000179

Measures	
-2*LogLikelihood	-531.6304
AICc	-527.1304
BIC	-525.0387

Goodness-of-Fit Test

	W	Prob<W
Shapiro-Wilk	0.9612109	0.3937

	A²	Simulated p-Value
Anderson-Darling	0.5180519	0.1712

Note: Ho = The data is from the Normal distribution. Small p-values reject Ho.

Test Mean

Hypothesized Value	0
Actual Estimate	-5.4e-6
DF	26
Std Dev	1.31e-5

	t Test	Signed-Rank
Test Statistic	-2.1629	-92.0000
Prob > |t|	0.0399*	0.0240*
Prob > t	0.9800	0.9880
Prob < t	0.0200*	0.0120*

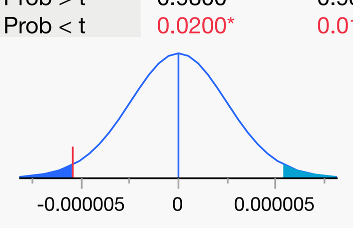

Supplement: Supplementary file 1 [file animals-14-01725-s001.zip › animals-3004812-supplementary/Table 4/Distribution and Test of Manure handling_Difference_NRC_IPCC.pdf]
