# Supplementary material for: Climate Change and Photochemical Ozone Creation Potential Impact Indicators of Cow Milk: A Comparison of Different Scenarios for a Diet Assessment
Source: Animals (Basel). 2024 Jun 7;14(12):1725. doi: 10.3390/ani14121725 (PMC11201073; doi:10.3390/ani14121725)

Distributions Herd=high-performing, Indicator=CC kgCO2eq

Total_Difference_NRC_IPCC

Compare Distributions

Show	Distribution		AICc ^	BIC	-2*LogLikelihood
☑	Normal		-18.97816	-18.79095	-24.06907

Summary Statistics

Mean	-0.141858
Std Dev	0.1061573
Std Err Mean	0.0283717
Upper 95% Mean	-0.080565
Lower 95% Mean	-0.203151
N	14
N Missing	0

Fitted Normal Distribution

Parameter		Estimate	Std Error	Lower 95%	Upper 95%
Location	μ	-0.141858	0.0283717	-0.203151	-0.080565
Dispersion	σ	0.1061573	0.0212315	0.0769591	0.1710238

Measures	
-2*LogLikelihood	-24.06907
AICc	-18.97816
BIC	-18.79095

Goodness-of-Fit Test

	W	Prob<W
Shapiro-Wilk	0.8861178	0.0711

	A^2	Simulated p-Value
Anderson-Darling	0.6382418	0.0844

Note: Ho = The data is from the Normal distribution. Small p-values reject Ho.

Test Mean

Hypothesized Value	0
Actual Estimate	-0.1419
DF	13
Std Dev	0.10616

	t Test	Signed-Rank
Test Statistic	-5.0000	-52.5000
Prob > |t|	0.0002*	0.0001*
Prob > t	0.9999	0.9999
Prob < t	0.0001*	<.0001*

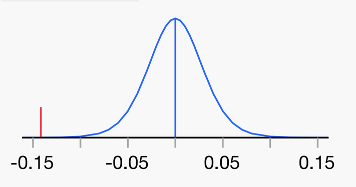

Distributions Herd=high-performing, Indicator=CC-biogenic kgCO2eq

Total_Difference_NRC_IPCC

Compare Distributions

Show	Distribution		AICc ^	BIC	-2*LogLikelihood
☑	Normal		-18.97816	-18.79095	-24.06906

Summary Statistics

Mean	-0.141858
Std Dev	0.1061573
Std Err Mean	0.0283717
Upper 95% Mean	-0.080565
Lower 95% Mean	-0.203151
N	14
N Missing	0

Fitted Normal Distribution

Parameter		Estimate	Std Error	Lower 95%	Upper 95%
Location	μ	-0.141858	0.0283717	-0.203151	-0.080565
Dispersion	σ	0.1061573	0.0212315	0.0769591	0.1710239

Measures	
-2*LogLikelihood	-24.06906
AICc	-18.97816
BIC	-18.79095

Goodness-of-Fit Test

	W	Prob<W
Shapiro-Wilk	0.8861178	0.0711

	A^2	Simulated p-Value
Anderson-Darling	0.6382418	0.0724

Note: Ho = The data is from the Normal distribution. Small p-values reject Ho.

Test Mean

Hypothesized Value	0
Actual Estimate	-0.1419
DF	13
Std Dev	0.10616

	t Test	Signed-Rank
Test Statistic	-5.0000	-52.5000
Prob > |t|	0.0002*	0.0001*
Prob > t	0.9999	0.9999
Prob < t	0.0001*	<.0001*

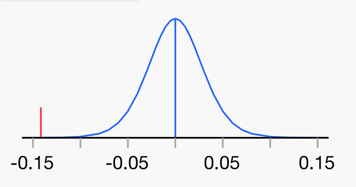

Distributions Herd=high-performing, Indicator=CC-fossil kgCO2eq

Total_Difference_NRC_IPCC

Compare Distributions

Show	Distribution		AICc ^	BIC	-2*LogLikelihood
☑	Normal		-483.9184	-483.7312	-489.0093

Summary Statistics

Mean	-5.083e-9
Std Dev	6.5236e-9
Std Err Mean	1.7435e-9
Upper 95% Mean	-1.316e-9
Lower 95% Mean	-8.849e-9
N	14
N Missing	0

Fitted Normal Distribution

Parameter		Estimate	Std Error	Lower 95%	Upper 95%
Location	μ	-5.083e-9	1.7435e-9	-8.849e-9	-1.316e-9
Dispersion	σ	6.5236e-9	1.3047e-9	4.7293e-9	1.051e-8

Measures	
-2*LogLikelihood	-489.0093
AICc	-483.9184
BIC	-483.7312

Goodness-of-Fit Test

	W	Prob<W
Shapiro-Wilk	0.7684642	0.0021*

	A^2	Simulated p-Value
Anderson-Darling	1.3385186	0.0008*

Note: Ho = The data is from the Normal distribution. Small p-values reject Ho.

Test Mean

Hypothesized Value	0
Actual Estimate	-5.1e-9
DF	13
Std Dev	6.52e-9

	t Test	Signed-Rank
Test Statistic	-2.9153	-52.5000
Prob > |t|	0.0120*	0.0001*
Prob > t	0.9940	0.9999
Prob < t	0.0060*	<.0001*

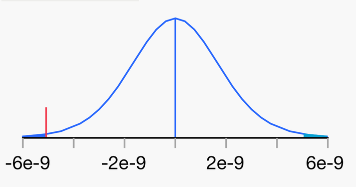

Distributions Herd=high-performing, Indicator=CC-LTU kgCO2eq

Total_Difference_NRC_IPCC

Summary Statistics

Mean	0
Std Dev	0
Std Err Mean	0
Upper 95% Mean	0
Lower 95% Mean	0
N	14
N Missing	0

Test Mean

Hypothesized Value	0
Actual Estimate	0
DF	13
Std Dev	0

	t Test	Signed-Rank
Test Statistic	.	0.0000
Prob > |t|	.	1.0000
Prob > t	.	0.5000
Prob < t	.	0.5000

Distributions Herd=high-performing, Indicator=POCP kgNMVOCeq

Total_Difference_NRC_IPCC

Compare Distributions

Show	Distribution		AICc ^	BIC	-2*LogLikelihood
☑	Normal		-246.3824	-246.1952	-251.4733

Summary Statistics

Mean	-4.214e-5
Std Dev	3.1535e-5
Std Err Mean	8.4281e-6
Upper 95% Mean	-0.000024
Lower 95% Mean	-6.035e-5
N	14
N Missing	0

Fitted Normal Distribution

Parameter		Estimate	Std Error	Lower 95%	Upper 95%
Location	μ	-4.214e-5	8.4281e-6	-6.035e-5	-0.000024
Dispersion	σ	3.1535e-5	6.307e-6	2.2861e-5	0.0000508

Measures	
-2*LogLikelihood	-251.4733
AICc	-246.3824
BIC	-246.1952

Goodness-of-Fit Test

	W	Prob<W
Shapiro-Wilk	0.8861178	0.0711

	A^2	Simulated p-Value
Anderson-Darling	0.6382418	0.0744

Note: Ho = The data is from the Normal distribution. Small p-values reject Ho.

Test Mean

Hypothesized Value	0
Actual Estimate	-4.2e-5
DF	13
Std Dev	3.15e-5

	t Test	Signed-Rank
Test Statistic	-5.0000	-52.5000
Prob > |t|	0.0002*	0.0001*
Prob > t	0.9999	0.9999
Prob < t	0.0001*	<.0001*

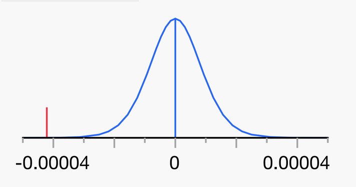

Distributions Herd=low-performing, Indicator=CC kgCO2eq

Total_Difference_NRC_IPCC

Compare Distributions

Show	Distribution		AICc ^	BIC	-2*LogLikelihood
☑	Normal		-6.784502	-6.597297	-11.87541

Summary Statistics

Mean	0.0238759
Std Dev	0.164089
Std Err Mean	0.0438546
Upper 95% Mean	0.1186181
Lower 95% Mean	-0.070866
N	14
N Missing	0

Fitted Normal Distribution

Parameter		Estimate	Std Error	Lower 95%	Upper 95%
Location	μ	0.0238759	0.0438546	-0.070866	0.1186181
Dispersion	σ	0.164089	0.0328178	0.118957	0.2643544

Measures	
-2*LogLikelihood	-11.87541
AICc	-6.784502
BIC	-6.597297

Goodness-of-Fit Test

	W	Prob<W
Shapiro-Wilk	0.9495903	0.5544

	A^2	Simulated p-Value
Anderson-Darling	0.3914057	0.3360

Note: Ho = The data is from the Normal distribution. Small p-values reject Ho.

Test Mean

Hypothesized Value	0
Actual Estimate	0.02388
DF	13
Std Dev	0.16409

	t Test	Signed-Rank
Test Statistic	0.5444	8.5000
Prob > |t|	0.5954	0.6257
Prob > t	0.2977	0.3129
Prob < t	0.7023	0.6871

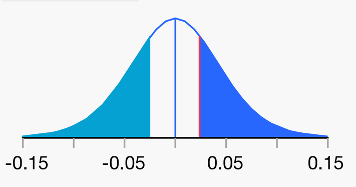

Distributions Herd=low-performing, Indicator=CC-biogenic kgCO2eq

Total_Difference_NRC_IPCC

Compare Distributions

Show	Distribution		AICc ^	BIC	-2*LogLikelihood
☑	Normal		-6.784503	-6.597298	-11.87541

Summary Statistics

Mean	0.0238759
Std Dev	0.164089
Std Err Mean	0.0438546
Upper 95% Mean	0.1186181
Lower 95% Mean	-0.070866
N	14
N Missing	0

Fitted Normal Distribution

Parameter		Estimate	Std Error	Lower 95%	Upper 95%
Location	μ	0.0238759	0.0438546	-0.070866	0.1186181
Dispersion	σ	0.164089	0.0328178	0.118957	0.2643544

Measures	
-2*LogLikelihood	-11.87541
AICc	-6.784503
BIC	-6.597298

Goodness-of-Fit Test

	W	Prob<W
Shapiro-Wilk	0.9495903	0.5544

	A^2	Simulated p-Value
Anderson-Darling	0.3914057	0.3568

Note: Ho = The data is from the Normal distribution. Small p-values reject Ho.

Test Mean

Hypothesized Value	0
Actual Estimate	0.02388
DF	13
Std Dev	0.16409

	t Test	Signed-Rank
Test Statistic	0.5444	8.5000
Prob > |t|	0.5954	0.6257
Prob > t	0.2977	0.3129
Prob < t	0.7023	0.6871

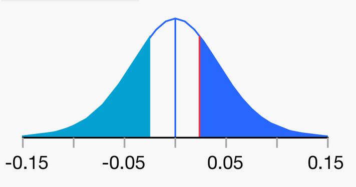

Distributions Herd=low-performing, Indicator=CC-fossil kgCO2eq

Total_Difference_NRC_IPCC

Compare Distributions

Show	Distribution		AICc ^	BIC	-2*LogLikelihood
☑	Normal		-458.9892	-458.802	-464.0801

Summary Statistics

Mean	-1.349e-8
Std Dev	1.5891e-8
Std Err Mean	4.247e-9
Upper 95% Mean	-4.318e-9
Lower 95% Mean	-2.267e-8
N	14
N Missing	0

Fitted Normal Distribution

Parameter		Estimate	Std Error	Lower 95%	Upper 95%
Location	μ	-1.349e-8	4.247e-9	-2.267e-8	-4.318e-9
Dispersion	σ	1.5891e-8	3.1782e-9	1.152e-8	2.5601e-8

Measures	
-2*LogLikelihood	-464.0801
AICc	-458.9892
BIC	-458.802

Goodness-of-Fit Test

	W	Prob<W
Shapiro-Wilk	0.7477248	0.0012*

	A^2	Simulated p-Value
Anderson-Darling	1.1415193	0.0024*

Note: Ho = The data is from the Normal distribution. Small p-values reject Ho.

Test Mean

Hypothesized Value	0
Actual Estimate	-1.3e-8
DF	13
Std Dev	1.59e-8

	t Test	Signed-Rank
Test Statistic	-3.1770	-52.5000
Prob > |t|	0.0073*	0.0001*
Prob > t	0.9964	0.9999
Prob < t	0.0036*	<.0001*

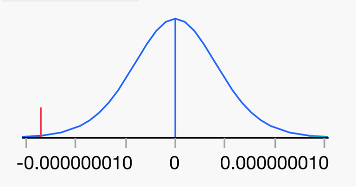

Distributions Herd=low-performing, Indicator=CC-LTU kgCO2eq

Total_Difference_NRC_IPCC

Summary Statistics

Mean	0
Std Dev	0
Std Err Mean	0
Upper 95% Mean	0
Lower 95% Mean	0
N	14
N Missing	0

Test Mean

Hypothesized Value	0
Actual Estimate	0
DF	13
Std Dev	0

	t Test	Signed-Rank
Test Statistic	.	0.0000
Prob > |t|	.	1.0000
Prob > t	.	0.5000
Prob < t	.	0.5000

Distributions Herd=low-performing, Indicator=POCP kgNMVOCeq

Total_Difference_NRC_IPCC

Compare Distributions

Show	Distribution		AICc ^	BIC	-2*LogLikelihood
☑	Normal		-234.1888	-234.0015	-239.2797

Summary Statistics

Mean	7.0925e-6
Std Dev	4.8744e-5
Std Err Mean	1.3027e-5
Upper 95% Mean	3.5237e-5
Lower 95% Mean	-0.000021
N	14
N Missing	0

Fitted Normal Distribution

Parameter		Estimate	Std Error	Lower 95%	Upper 95%
Location	μ	7.0925e-6	1.3027e-5	-0.000021	3.5237e-5
Dispersion	σ	4.8744e-5	9.7488e-6	3.5337e-5	7.8529e-5

Measures	
-2*LogLikelihood	-239.2797
AICc	-234.1888
BIC	-234.0015

Goodness-of-Fit Test

	W	Prob<W
Shapiro-Wilk	0.9495903	0.5544

	A^2	Simulated p-Value
Anderson-Darling	0.3914057	0.3616

Note: Ho = The data is from the Normal distribution. Small p-values reject Ho.

Test Mean

Hypothesized Value	0
Actual Estimate	7.09e-6
DF	13
Std Dev	4.87e-5

	t Test	Signed-Rank
Test Statistic	0.5444	8.5000
Prob > |t|	0.5954	0.6257
Prob > t	0.2977	0.3129
Prob < t	0.7023	0.6871

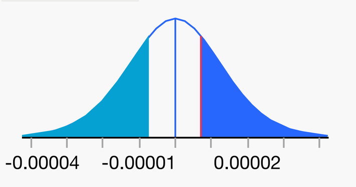

Distributions Herd=mid-performing, Indicator=CC kgCO2eq

Total_Difference_NRC_IPCC

Compare Distributions

Show	Distribution		AICc ^	BIC	-2*LogLikelihood
☑	Normal		-43.32999	-41.23832	-47.82999

Summary Statistics

Mean	-0.061846
Std Dev	0.101656
Std Err Mean	0.0195637
Upper 95% Mean	-0.021633
Lower 95% Mean	-0.10206
N	27
N Missing	0

Fitted Normal Distribution

Parameter		Estimate	Std Error	Lower 95%	Upper 95%
Location	μ	-0.061846	0.0195637	-0.10206	-0.021633
Dispersion	σ	0.101656	0.0142347	0.0800558	0.1393126

Measures	
-2*LogLikelihood	-47.82999
AICc	-43.32999
BIC	-41.23832

Goodness-of-Fit Test

	W	Prob<W
Shapiro-Wilk	0.931737	0.0762

	A^2	Simulated p-Value
Anderson-Darling	0.6395498	0.0924

Note: Ho = The data is from the Normal distribution. Small p-values reject Ho.

Test Mean

Hypothesized Value	0
Actual Estimate	-0.0618
DF	26
Std Dev	0.10166

	t Test	Signed-Rank
Test Statistic	-3.1613	-132.000
Prob > |t|	0.0040*	0.0006*
Prob > t	0.9980	0.9997
Prob < t	0.0020*	0.0003*

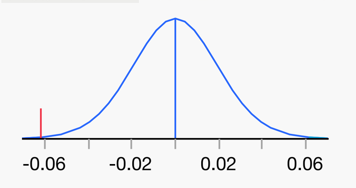

Distributions Herd=mid-performing, Indicator=CC-biogenic kgCO2eq

Total_Difference_NRC_IPCC

Compare Distributions

Show	Distribution		AICc ^	BIC	-2*LogLikelihood
☑	Normal		-43.32999	-41.23832	-47.82999

Summary Statistics

Mean	-0.061846
Std Dev	0.101656
Std Err Mean	0.0195637
Upper 95% Mean	-0.021633
Lower 95% Mean	-0.10206
N	27
N Missing	0

Fitted Normal Distribution

Parameter		Estimate	Std Error	Lower 95%	Upper 95%
Location	μ	-0.061846	0.0195637	-0.10206	-0.021633
Dispersion	σ	0.101656	0.0142347	0.0800558	0.1393126

Measures	
-2*LogLikelihood	-47.82999
AICc	-43.32999
BIC	-41.23832

Goodness-of-Fit Test

	W	Prob<W
Shapiro-Wilk	0.931737	0.0762

	A^2	Simulated p-Value
Anderson-Darling	0.6395499	0.0844

Note: Ho = The data is from the Normal distribution. Small p-values reject Ho.

Test Mean

Hypothesized Value	0
Actual Estimate	-0.0618
DF	26
Std Dev	0.10166

	t Test	Signed-Rank
Test Statistic	-3.1613	-132.000
Prob > |t|	0.0040*	0.0006*
Prob > t	0.9980	0.9997
Prob < t	0.0020*	0.0003*

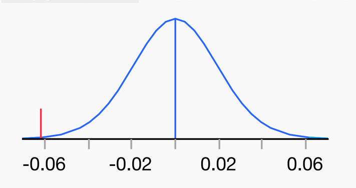

Distributions Herd=mid-performing, Indicator=CC-fossil kgCO2eq

Total_Difference_NRC_IPCC

Compare Distributions

Show	Distribution		AICc ^	BIC	-2*LogLikelihood
☑	Normal		-936.5848	-934.4931	-941.0848

Summary Statistics

Mean	-7.922e-9
Std Dev	6.6549e-9
Std Err Mean	1.2807e-9
Upper 95% Mean	-5.29e-9
Lower 95% Mean	-1.055e-8
N	27
N Missing	0

Fitted Normal Distribution

Parameter		Estimate	Std Error	Lower 95%	Upper 95%
Location	μ	-7.922e-9	1.2807e-9	-1.055e-8	-5.29e-9
Dispersion	σ	6.6549e-9	9.319e-10	5.2408e-9	9.1201e-9

Measures	
-2*LogLikelihood	-941.0848
AICc	-936.5848
BIC	-934.4931

Goodness-of-Fit Test

	W	Prob<W
Shapiro-Wilk	0.8169248	0.0003*

	A^2	Simulated p-Value
Anderson-Darling	2.1840441	<.0001*

Note: Ho = The data is from the Normal distribution. Small p-values reject Ho.

Test Mean

Hypothesized Value	0
Actual Estimate	-7.9e-9
DF	26
Std Dev	6.65e-9

	t Test	Signed-Rank
Test Statistic	-6.1857	-189.000
Prob > |t|	<.0001*	<.0001*
Prob > t	1.0000	1.0000
Prob < t	<.0001*	<.0001*

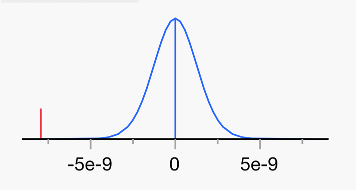

Distributions Herd=mid-performing, Indicator=CC-LTU kgCO2eq

Total_Difference_NRC_IPCC

Summary Statistics

Mean	0
Std Dev	0
Std Err Mean	0
Upper 95% Mean	0
Lower 95% Mean	0
N	27
N Missing	0

Test Mean

Hypothesized Value	0
Actual Estimate	0
DF	26
Std Dev	0

	t Test	Signed-Rank
Test Statistic	.	0.0000
Prob > |t|	.	1.0000
Prob > t	.	0.5000
Prob < t	.	0.5000

Distributions Herd=mid-performing, Indicator=POCP kgNMVOCeq

Total_Difference_NRC_IPCC

Compare Distributions

Show	Distribution		AICc ^	BIC	-2*LogLikelihood
☑	Normal		-481.8953	-479.8037	-486.3953

Summary Statistics

Mean	-1.837e-5
Std Dev	0.0000302
Std Err Mean	5.8116e-6
Upper 95% Mean	-6.426e-6
Lower 95% Mean	-3.032e-5
N	27
N Missing	0

Fitted Normal Distribution

Parameter		Estimate	Std Error	Lower 95%	Upper 95%
Location	μ	-1.837e-5	5.8116e-6	-3.032e-5	-6.426e-6
Dispersion	σ	0.0000302	4.2285e-6	2.3781e-5	4.1384e-5

Measures	
-2*LogLikelihood	-486.3953
AICc	-481.8953
BIC	-479.8037

Goodness-of-Fit Test

	W	Prob<W
Shapiro-Wilk	0.931737	0.0762

	A^2	Simulated p-Value
Anderson-Darling	0.63955	0.0908

Note: Ho = The data is from the Normal distribution. Small p-values reject Ho.

Test Mean

Hypothesized Value	0
Actual Estimate	-1.8e-5
DF	26
Std Dev	0.00003

	t Test	Signed-Rank
Test Statistic	-3.1613	-132.000
Prob > |t|	0.0040*	0.0006*
Prob > t	0.9980	0.9997
Prob < t	0.0020*	0.0003*

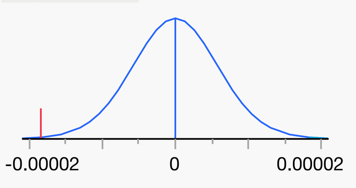

Supplement: Supplementary file 1 [file animals-14-01725-s001.zip › animals-3004812-supplementary/Table 4/Distribution and Test of Total_Difference_NRC_IPCC.pdf]
