# Supplementary material for: Climate Change and Photochemical Ozone Creation Potential Impact Indicators of Cow Milk: A Comparison of Different Scenarios for a Diet Assessment
Source: Animals (Basel). 2024 Jun 7;14(12):1725. doi: 10.3390/ani14121725 (PMC11201073; doi:10.3390/ani14121725)

Distributions Herd=high-performing, Indicator=CC kgCO2eq

Manure handling_Difference_CNCPS_IPCC

Compare Distributions

Show	Distribution		AICc ^	BIC	-2*LogLikelihood
☑	Normal		-49.75424	-49.56703	-54.84515

Summary Statistics

Mean	-0.080827
Std Dev	0.0353669
Std Err Mean	0.0094522
Upper 95% Mean	-0.060407
Lower 95% Mean	-0.101247
N	14
N Missing	0

Fitted Normal Distribution

Parameter		Estimate	Std Error	Lower 95%	Upper 95%
Location	μ	-0.080827	0.0094522	-0.101247	-0.060407
Dispersion	σ	0.0353669	0.0070734	0.0256394	0.0569775

Measures	
-2*LogLikelihood	-54.84515
AICc	-49.75424
BIC	-49.56703

Goodness-of-Fit Test

	W	Prob<W
Shapiro-Wilk	0.9850247	0.9942

	A²	Simulated p-Value
Anderson-Darling	0.1677101	0.9388

Note: Ho = The data is from the Normal distribution. Small p-values reject Ho.

Test Mean

Hypothesized Value	0
Actual Estimate	-0.0808
DF	13
Std Dev	0.03537

	t Test	Signed-Rank
Test Statistic	-8.5511	-52.5000
Prob > |t|	<.0001*	0.0001*
Prob > t	1.0000	0.9999
Prob < t	<.0001*	<.0001*

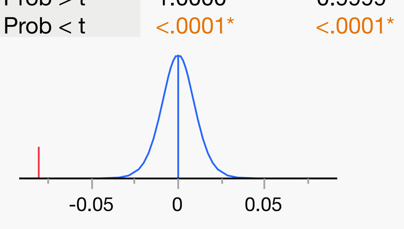

Distributions Herd=high-performing, Indicator=CC-biogenic kgCO2eq

Manure handling_Difference_CNCPS_IPCC

Compare Distributions

Show	Distribution		AICc ^	BIC	-2*LogLikelihood
☑	Normal		-49.75424	-49.56703	-54.84515

Summary Statistics

Mean	-0.080827
Std Dev	0.0353669
Std Err Mean	0.0094522
Upper 95% Mean	-0.060407
Lower 95% Mean	-0.101247
N	14
N Missing	0

Fitted Normal Distribution

Parameter		Estimate	Std Error	Lower 95%	Upper 95%
Location	μ	-0.080827	0.0094522	-0.101247	-0.060407
Dispersion	σ	0.0353669	0.0070734	0.0256394	0.0569775

Measures	
-2*LogLikelihood	-54.84515
AICc	-49.75424
BIC	-49.56703

Goodness-of-Fit Test

	W	Prob<W
Shapiro-Wilk	0.9850247	0.9942

	A²	Simulated p-Value
Anderson-Darling	0.16771	0.9416

Note: Ho = The data is from the Normal distribution. Small p-values reject Ho.

Test Mean

Hypothesized Value	0
Actual Estimate	-0.0808
DF	13
Std Dev	0.03537

	t Test	Signed-Rank
Test Statistic	-8.5511	-52.5000
Prob > |t|	<.0001*	0.0001*
Prob > t	1.0000	0.9999
Prob < t	<.0001*	<.0001*

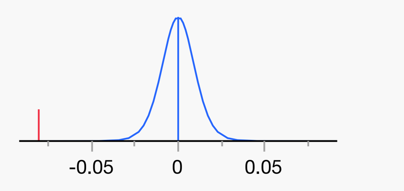

Distributions Herd=high-performing, Indicator=CC-fossil kgCO2eq

Manure handling_Difference_CNCPS_IPCC

Summary Statistics

Mean	0
Std Dev	0
Std Err Mean	0
Upper 95% Mean	0
Lower 95% Mean	0
N	14
N Missing	0

Test Mean

Hypothesized Value	0
Actual Estimate	0
DF	13
Std Dev	0

	t Test	Signed-Rank
Test Statistic	.	0.0000
Prob > |t|	.	1.0000
Prob > t	.	0.5000
Prob < t	.	0.5000

Distributions Herd=high-performing, Indicator=CC-LTU kgCO2eq

Manure handling_Difference_CNCPS_IPCC

Summary Statistics

Mean	0
Std Dev	0
Std Err Mean	0
Upper 95% Mean	0
Lower 95% Mean	0
N	14
N Missing	0

Test Mean

Hypothesized Value	0
Actual Estimate	0
DF	13
Std Dev	0

	t Test	Signed-Rank
Test Statistic	.	0.0000
Prob > |t|	.	1.0000
Prob > t	.	0.5000
Prob < t	.	0.5000

Distributions Herd=high-performing, Indicator=POCP kgNMVOCeq

Manure handling_Difference_CNCPS_IPCC

Compare Distributions

Show	Distribution		AICc ^	BIC	-2*LogLikelihood
☑	Normal		-277.1585	-276.9713	-282.2494

Summary Statistics

Mean	-0.000024
Std Dev	0.0000105
Std Err Mean	2.8079e-6
Upper 95% Mean	-0.000018
Lower 95% Mean	-0.00003
N	14
N Missing	0

Fitted Normal Distribution

Parameter		Estimate	Std Error	Lower 95%	Upper 95%
Location	μ	-0.000024	2.8079e-6	-0.00003	-0.000018
Dispersion	σ	0.0000105	2.1012e-6	7.6164e-6	1.6926e-5

Measures	
-2*LogLikelihood	-282.2494
AICc	-277.1585
BIC	-276.9713

Goodness-of-Fit Test

	W	Prob<W
Shapiro-Wilk	0.9850246	0.9942

	A²	Simulated p-Value
Anderson-Darling	0.1677101	0.9428

Note: Ho = The data is from the Normal distribution. Small p-values reject Ho.

Test Mean

Hypothesized Value	0
Actual Estimate	-2.4e-5
DF	13
Std Dev	0.00001

	t Test	Signed-Rank
Test Statistic	-8.5511	-52.5000
Prob > |t|	<.0001*	0.0001*
Prob > t	1.0000	0.9999
Prob < t	<.0001*	<.0001*

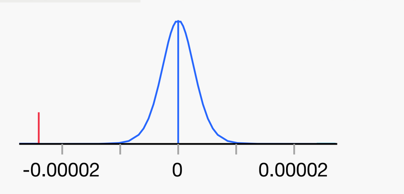

Distributions Herd=low-performing, Indicator=CC kgCO2eq

Manure handling_Difference_CNCPS_IPCC

Compare Distributions

Show	Distribution		AICc ^	BIC	-2*LogLikelihood
☑	Normal		-40.00494	-39.81773	-45.09585

Summary Statistics

Mean	-0.038654
Std Dev	0.0500972
Std Err Mean	0.013389
Upper 95% Mean	-0.009728
Lower 95% Mean	-0.067579
N	14
N Missing	0

Fitted Normal Distribution

Parameter		Estimate	Std Error	Lower 95%	Upper 95%
Location	μ	-0.038654	0.013389	-0.067579	-0.009728
Dispersion	σ	0.0500972	0.0100194	0.0363182	0.0807087

Measures	
-2*LogLikelihood	-45.09585
AICc	-40.00494
BIC	-39.81773

Goodness-of-Fit Test

	W	Prob<W
Shapiro-Wilk	0.9583127	0.6952

	A²	Simulated p-Value
Anderson-Darling	0.2434308	0.7448

Note: Ho = The data is from the Normal distribution. Small p-values reject Ho.

Test Mean

Hypothesized Value	0
Actual Estimate	-0.0387
DF	13
Std Dev	0.0501

	t Test	Signed-Rank
Test Statistic	-2.8870	-36.5000
Prob > |t|	0.0127*	0.0203*
Prob > t	0.9936	0.9899
Prob < t	0.0064*	0.0101*

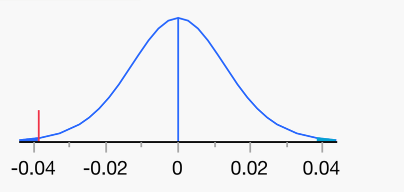

Distributions Herd=low-performing, Indicator=CC-biogenic kgCO2eq

Manure handling_Difference_CNCPS_IPCC

Compare Distributions

Show	Distribution		AICc ^	BIC	-2*LogLikelihood
☑	Normal		-40.00494	-39.81773	-45.09585

Summary Statistics

Mean	-0.038654
Std Dev	0.0500972
Std Err Mean	0.013389
Upper 95% Mean	-0.009728
Lower 95% Mean	-0.067579
N	14
N Missing	0

Fitted Normal Distribution

Parameter		Estimate	Std Error	Lower 95%	Upper 95%
Location	μ	-0.038654	0.013389	-0.067579	-0.009728
Dispersion	σ	0.0500972	0.0100194	0.0363182	0.0807087

Measures	
-2*LogLikelihood	-45.09585
AICc	-40.00494
BIC	-39.81773

Goodness-of-Fit Test

	W	Prob<W
Shapiro-Wilk	0.9583128	0.6952

	A²	Simulated p-Value
Anderson-Darling	0.2434308	0.7404

Note: Ho = The data is from the Normal distribution. Small p-values reject Ho.

Test Mean

Hypothesized Value	0
Actual Estimate	-0.0387
DF	13
Std Dev	0.0501

	t Test	Signed-Rank
Test Statistic	-2.8870	-36.5000
Prob > |t|	0.0127*	0.0203*
Prob > t	0.9936	0.9899
Prob < t	0.0064*	0.0101*

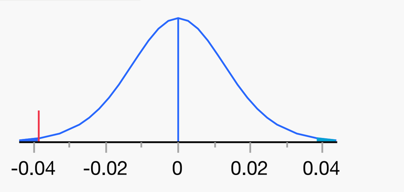

Distributions Herd=low-performing, Indicator=CC-fossil kgCO2eq

Manure handling_Difference_CNCPS_IPCC

Summary Statistics

Mean	0
Std Dev	0
Std Err Mean	0
Upper 95% Mean	0
Lower 95% Mean	0
N	14
N Missing	0

Test Mean

Hypothesized Value	0
Actual Estimate	0
DF	13
Std Dev	0

	t Test	Signed-Rank
Test Statistic	.	0.0000
Prob > |t|	.	1.0000
Prob > t	.	0.5000
Prob < t	.	0.5000

Distributions Herd=low-performing, Indicator=CC-LTU kgCO2eq

Manure handling_Difference_CNCPS_IPCC

Summary Statistics

Mean	0
Std Dev	0
Std Err Mean	0
Upper 95% Mean	0
Lower 95% Mean	0
N	14
N Missing	0

Test Mean

Hypothesized Value	0
Actual Estimate	0
DF	13
Std Dev	0

	t Test	Signed-Rank
Test Statistic	.	0.0000
Prob > |t|	.	1.0000
Prob > t	.	0.5000
Prob < t	.	0.5000

Distributions Herd=low-performing, Indicator=POCP kgNMVOCeq

Manure handling_Difference_CNCPS_IPCC

Compare Distributions

Show	Distribution		AICc ^	BIC	-2*LogLikelihood
☑	Normal		-267.4092	-267.222	-272.5001

Summary Statistics

Mean	-1.148e-5
Std Dev	1.4882e-5
Std Err Mean	3.9773e-6
Upper 95% Mean	-2.89e-6
Lower 95% Mean	-0.00002
N	14
N Missing	0

Fitted Normal Distribution

Parameter		Estimate	Std Error	Lower 95%	Upper 95%
Location	μ	-1.148e-5	3.9773e-6	-0.00002	-2.89e-6
Dispersion	σ	1.4882e-5	2.9764e-6	1.0789e-5	2.3975e-5

Measures	
-2*LogLikelihood	-272.5001
AICc	-267.4092
BIC	-267.222

Goodness-of-Fit Test

	W	Prob<W
Shapiro-Wilk	0.9583128	0.6952

	A²	Simulated p-Value
Anderson-Darling	0.2434307	0.7528

Note: Ho = The data is from the Normal distribution. Small p-values reject Ho.

Test Mean

Hypothesized Value	0
Actual Estimate	-1.1e-5
DF	13
Std Dev	1.49e-5

	t Test	Signed-Rank
Test Statistic	-2.8870	-36.5000
Prob > |t|	0.0127*	0.0203*
Prob > t	0.9936	0.9899
Prob < t	0.0064*	0.0101*

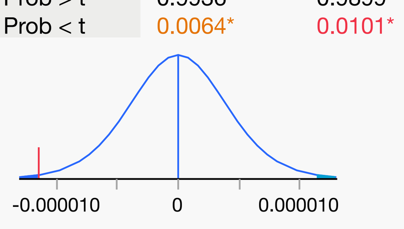

Distributions Herd=mid-performing, Indicator=CC kgCO2eq

Manure handling_Difference_CNCPS_IPCC

Compare Distributions

Show	Distribution		AICc ^	BIC	-2*LogLikelihood
☑	Normal		-85.70836	-83.61668	-90.20836

Summary Statistics

Mean	-0.075782
Std Dev	0.0463773
Std Err Mean	0.0089253
Upper 95% Mean	-0.057436
Lower 95% Mean	-0.094128
N	27
N Missing	0

Fitted Normal Distribution

Parameter		Estimate	Std Error	Lower 95%	Upper 95%
Location	μ	-0.075782	0.0089253	-0.094128	-0.057436
Dispersion	σ	0.0463773	0.0064941	0.0365229	0.0635569

Measures	
-2*LogLikelihood	-90.20836
AICc	-85.70836
BIC	-83.61668

Goodness-of-Fit Test

	W	Prob<W
Shapiro-Wilk	0.9426446	0.1416

	A²	Simulated p-Value
Anderson-Darling	0.5830811	0.1284

Note: Ho = The data is from the Normal distribution. Small p-values reject Ho.

Test Mean

Hypothesized Value	0
Actual Estimate	-0.0758
DF	26
Std Dev	0.04638

	t Test	Signed-Rank
Test Statistic	-8.4907	-188.000
Prob > |t|	<.0001*	<.0001*
Prob > t	1.0000	1.0000
Prob < t	<.0001*	<.0001*

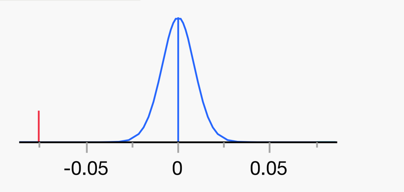

Distributions Herd=mid-performing, Indicator=CC-biogenic kgCO2eq

Manure handling_Difference_CNCPS_IPCC

Compare Distributions

Show	Distribution		AICc ^	BIC	-2*LogLikelihood
☑	Normal		-85.70836	-83.61668	-90.20836

Summary Statistics

Mean	-0.075782
Std Dev	0.0463773
Std Err Mean	0.0089253
Upper 95% Mean	-0.057436
Lower 95% Mean	-0.094128
N	27
N Missing	0

Fitted Normal Distribution

Parameter		Estimate	Std Error	Lower 95%	Upper 95%
Location	μ	-0.075782	0.0089253	-0.094128	-0.057436
Dispersion	σ	0.0463773	0.0064941	0.0365229	0.0635569

Measures	
-2*LogLikelihood	-90.20836
AICc	-85.70836
BIC	-83.61668

Goodness-of-Fit Test

	W	Prob<W
Shapiro-Wilk	0.9426446	0.1416

	A²	Simulated p-Value
Anderson-Darling	0.5830811	0.1172

Note: Ho = The data is from the Normal distribution. Small p-values reject Ho.

Test Mean

Hypothesized Value	0
Actual Estimate	-0.0758
DF	26
Std Dev	0.04638

	t Test	Signed-Rank
Test Statistic	-8.4907	-188.000
Prob > |t|	<.0001*	<.0001*
Prob > t	1.0000	1.0000
Prob < t	<.0001*	<.0001*

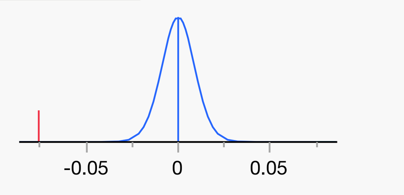

Distributions Herd=mid-performing, Indicator=CC-fossil kgCO2eq

Manure handling_Difference_CNCPS_IPCC

Summary Statistics

Mean	0
Std Dev	0
Std Err Mean	0
Upper 95% Mean	0
Lower 95% Mean	0
N	27
N Missing	0

Test Mean

Hypothesized Value	0
Actual Estimate	0
DF	26
Std Dev	0

	t Test	Signed-Rank
Test Statistic	.	0.0000
Prob > |t|	.	1.0000
Prob > t	.	0.5000
Prob < t	.	0.5000

Distributions Herd=mid-performing, Indicator=CC-LTU kgCO2eq

Manure handling_Difference_CNCPS_IPCC

Summary Statistics

Mean	0
Std Dev	0
Std Err Mean	0
Upper 95% Mean	0
Lower 95% Mean	0
N	27
N Missing	0

Test Mean

Hypothesized Value	0
Actual Estimate	0
DF	26
Std Dev	0

	t Test	Signed-Rank
Test Statistic	.	0.0000
Prob > |t|	.	1.0000
Prob > t	.	0.5000
Prob < t	.	0.5000

Distributions Herd=mid-performing, Indicator=POCP kgNMVOCeq

Manure handling_Difference_CNCPS_IPCC

Compare Distributions

Show	Distribution		AICc ^	BIC	-2*LogLikelihood
☑	Normal		-524.2737	-522.182	-528.7737

Summary Statistics

Mean	-2.251e-5
Std Dev	1.3777e-5
Std Err Mean	2.6513e-6
Upper 95% Mean	-0.000017
Lower 95% Mean	-0.000028
N	27
N Missing	0

Fitted Normal Distribution

Parameter		Estimate	Std Error	Lower 95%	Upper 95%
Location	μ	-2.251e-5	2.6513e-6	-0.000028	-0.000017
Dispersion	σ	1.3777e-5	1.9291e-6	1.0849e-5	1.888e-5

Measures	
-2*LogLikelihood	-528.7737
AICc	-524.2737
BIC	-522.182

Goodness-of-Fit Test

	W	Prob<W
Shapiro-Wilk	0.9426445	0.1416

	A²	Simulated p-Value
Anderson-Darling	0.5830815	0.1260

Note: Ho = The data is from the Normal distribution. Small p-values reject Ho.

Test Mean

Hypothesized Value	0
Actual Estimate	-2.3e-5
DF	26
Std Dev	1.38e-5

	t Test	Signed-Rank
Test Statistic	-8.4907	-188.000
Prob > |t|	<.0001*	<.0001*
Prob > t	1.0000	1.0000
Prob < t	<.0001*	<.0001*

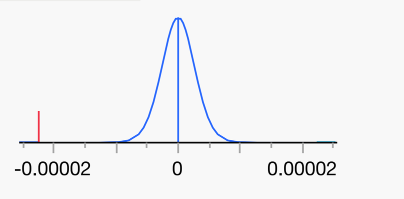

Supplement: Supplementary file 1 [file Distribution and Test of Manure handling_Difference_CNCPS_IPCC.pdf › animals-3004812-supplementary/Tabel 5/Distribution and Test of Manure handling_Difference_CNCPS_IPCC.pdf]
