# Supplementary material for: Climate Change and Photochemical Ozone Creation Potential Impact Indicators of Cow Milk: A Comparison of Different Scenarios for a Diet Assessment
Source: Animals (Basel). 2024 Jun 7;14(12):1725. doi: 10.3390/ani14121725 (PMC11201073; doi:10.3390/ani14121725)
Supplement: Supplementary file 1 [file Anova of Energy.pdf › animals-3004812-supplementary/Table 3/Anova of Energy.pdf]

Oneway Analysis of Energy By Herd Indicator=CC kgCO2eq

Oneway Anova

Summary of Fit

Rsquare	0.408645
Adj Rsquare	0.3859
Root Mean Square Error	0.025902
Mean of Response	0.08341
Observations (or Sum Wgts)	55

Analysis of Variance

Source	DF	Sum of Squares	Mean Square	F Ratio	Prob > F
Herd	2	0.02410848	0.012054	17.9668	<.0001*
Error	52	0.03488767	0.000671		
C. Total	54	0.05899615			

Means for Oneway Anova

Level	Number	Mean	Std Error	Lower 95%	Upper 95%
high-performing	14	0.058199	0.00692	0.04431	0.07209
low-performing	14	0.115947	0.00692	0.10206	0.12984
mid-performing	27	0.079612	0.00498	0.06961	0.08962

Std Error uses a pooled estimate of error variance

Nonparametric Comparisons For All Pairs Using Steel-Dwass Method

q*	Alpha
2.34370	0.05

Level	- Level	Score Mean Difference	Std Err Dif	Z	p-Value	Hodges-Lehmann	Lower CL	Upper CL	Difference Plot
low-performing	high-performing	12.3571	3.109126	3.97447	0.0002*	0.058144	0.029043	0.084936	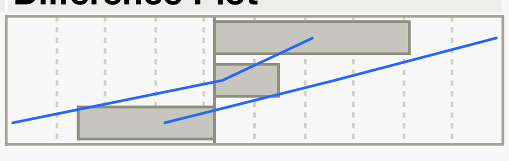
mid-performing	high-performing	10.2500	3.945227	2.59808	0.0254*	0.019302	0.001871	0.036082	
mid-performing	low-performing	-13.3955	3.945227	-3.39537	0.0020*	-0.041290	-0.061410	-0.015482	

Excluded Rows 3

Oneway Analysis of Energy By Herd Indicator=CC-biogenic kgCO2eq

Oneway Anova

Summary of Fit

Rsquare	0.394419
Adj Rsquare	0.371127
Root Mean Square Error	0.000042
Mean of Response	0.00011
Observations (or Sum Wgts)	55

Analysis of Variance

Source	DF	Sum of Squares	Mean Square	F Ratio	Prob > F
Herd	2	5.9602e-8	2.9801e-8	16.9340	<.0001*
Error	52	9.15116e-8	1.7598e-9		
C. Total	54	1.51114e-7			

Means for Oneway Anova

Level	Number	Mean	Std Error	Lower 95%	Upper 95%
high-performing	14	0.000071	1.12e-5	4.89e-5	9.39e-5
low-performing	14	0.000162	1.12e-5	0.00014	0.00018
mid-performing	27	0.000103	8.07e-6	8.71e-5	0.00012

Std Error uses a pooled estimate of error variance

Nonparametric Comparisons For All Pairs Using Steel-Dwass Method

q*	Alpha
2.34370	0.05

Level	- Level	Score Mean Difference	Std Err Dif	Z	p-Value	Hodges-Lehmann	Lower CL	Upper CL	Difference Plot
low-performing	high-performing	13.0714	3.109126	4.20421	<.0001*	0.000094	0.000046	0.000135	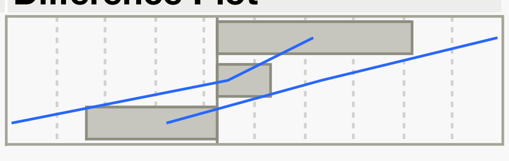
mid-performing	high-performing	10.6839	3.945227	2.70805	0.0186*	0.000025	4.4902e-6	0.000050	
mid-performing	low-performing	-13.8294	3.945227	-3.50534	0.0013*	-0.000064	-0.000099	-0.000025	

Excluded Rows 3

Oneway Analysis of Energy By Herd Indicator=CC-fossil kgCO2eq

Oneway Anova

Summary of Fit

Rsquare	0.408579
Adj Rsquare	0.385832
Root Mean Square Error	0.025701
Mean of Response	0.082869
Observations (or Sum Wgts)	55

Analysis of Variance

Source	DF	Sum of Squares	Mean Square	F Ratio	Prob > F
Herd	2	0.02372899	0.011864	17.9619	<.0001*
Error	52	0.03434792	0.000661		
C. Total	54	0.05807691			

Means for Oneway Anova

Level	Number	Mean	Std Error	Lower 95%	Upper 95%
high-performing	14	0.057851	0.00687	0.04407	0.07163
low-performing	14	0.115145	0.00687	0.10136	0.12893
mid-performing	27	0.079105	0.00495	0.06918	0.08903

Std Error uses a pooled estimate of error variance

Nonparametric Comparisons For All Pairs Using Steel-Dwass Method

q*	Alpha
2.34370	0.05

Level	- Level	Score Mean Difference	Std Err Dif	Z	p-Value	Hodges-Lehmann	Lower CL	Upper CL	Difference Plot
low-performing	high-performing	12.3571	3.109126	3.97447	0.0002*	0.057865	0.028798	0.084325	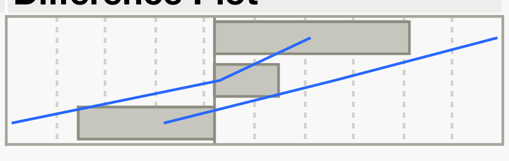
mid-performing	high-performing	10.1415	3.945227	2.57058	0.0274*	0.019272	0.001699	0.035892	
mid-performing	low-performing	-13.2870	3.945227	-3.36788	0.0022*	-0.040931	-0.060845	-0.015291	

Excluded Rows 3

Oneway Analysis of Energy By Herd Indicator=CC-LTU kgCO2eq

Oneway Anova

Summary of Fit

Rsquare	0.389609
Adj Rsquare	0.366133
Root Mean Square Error	0.000171
Mean of Response	0.000432
Observations (or Sum Wgts)	55

Analysis of Variance

Source	DF	Sum of Squares	Mean Square	F Ratio	Prob > F
Herd	2	9.66653e-7	4.8333e-7	16.5957	<.0001*
Error	52	1.51443e-6	2.9124e-8		
C. Total	54	2.48108e-6			

Means for Oneway Anova

Level	Number	Mean	Std Error	Lower 95%	Upper 95%
high-performing	14	0.000276	4.56e-5	0.00018	0.00037
low-performing	14	0.000640	4.56e-5	0.00055	0.00073
mid-performing	27	0.000404	3.28e-5	0.00034	0.00047

Std Error uses a pooled estimate of error variance

Nonparametric Comparisons For All Pairs Using Steel-Dwass Method

q*	Alpha
2.34370	0.05

Level	- Level	Score Mean Difference	Std Err Dif	Z	p-Value	Hodges-Lehmann	Lower CL	Upper CL	Difference Plot
low-performing	high-performing	13.0714	3.109126	4.20421	<.0001*	0.000368	0.000184	0.000545	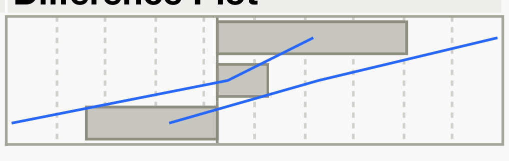
mid-performing	high-performing	10.7923	3.945227	2.73554	0.0171*	0.000098	0.000017	0.000196	
mid-performing	low-performing	-13.9378	3.945227	-3.53283	0.0012*	-0.000259	-0.000404	-0.000095	

Excluded Rows 3

Oneway Analysis of Energy By Herd Indicator=POCP kgNMVOCeq

Oneway Anova

Summary of Fit

Rsquare	0.392565
Adj Rsquare	0.369202
Root Mean Square Error	0.000396
Mean of Response	0.001021
Observations (or Sum Wgts)	55

Analysis of Variance

Source	DF	Sum of Squares	Mean Square	F Ratio	Prob > F
Herd	2	0.00000526	2.6295e-6	16.8029	<.0001*
Error	52	0.00000814	1.5649e-7		
C. Total	54	0.00001340			

Means for Oneway Anova

Level	Number	Mean	Std Error	Lower 95%	Upper 95%
high-performing	14	0.000658	0.00011	0.00045	0.00087
low-performing	14	0.001507	0.00011	0.00129	0.00172
mid-performing	27	0.000957	7.61e-5	0.00080	0.00111

Std Error uses a pooled estimate of error variance

Nonparametric Comparisons For All Pairs Using Steel-Dwass Method

q*	Alpha
2.34370	0.05

Level	- Level	Score Mean Difference	Std Err Dif	Z	p-Value	Hodges-Lehmann	Lower CL	Upper CL	Difference Plot
low-performing	high-performing	13.0714	3.109126	4.20421	<.0001*	0.000867	0.000436	0.001263	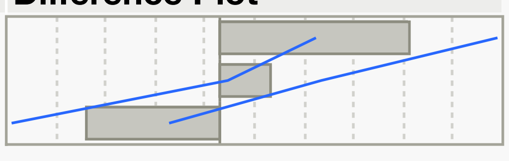
mid-performing	high-performing	10.7923	3.945227	2.73554	0.0171*	0.000237	0.000045	0.000460	
mid-performing	low-performing	-13.9378	3.945227	-3.53283	0.0012*	-0.000595	-0.000938	-0.000229	

Excluded Rows 3
