# Supplementary material for: Climate Change and Photochemical Ozone Creation Potential Impact Indicators of Cow Milk: A Comparison of Different Scenarios for a Diet Assessment
Source: Animals (Basel). 2024 Jun 7;14(12):1725. doi: 10.3390/ani14121725 (PMC11201073; doi:10.3390/ani14121725)
Supplement: Supplementary file 1 [file Anova of Feeds purchase.pdf › animals-3004812-supplementary/Table 3/Anova of Feeds purchase.pdf]

Oneway Analysis of Feeds purchase By Herd Indicator=CC kgCO2eq

Oneway Anova

Summary of Fit

Rsquare	0.075453
Adj Rsquare	0.039894
Root Mean Square Error	0.188377
Mean of Response	0.579992
Observations (or Sum Wgts)	55

Analysis of Variance

Source	DF	Sum of Squares	Mean Square	F Ratio	Prob > F
Herd	2	0.1505947	0.075297	2.1219	0.1301
Error	52	1.8452690	0.035486		
C. Total	54	1.9958637			

Means for Oneway Anova

Level	Number	Mean	Std Error	Lower 95%	Upper 95%
high-performing	14	0.496929	0.05035	0.39590	0.59796
low-performing	14	0.639799	0.05035	0.53877	0.74083
mid-performing	27	0.592050	0.03625	0.51930	0.66480

Std Error uses a pooled estimate of error variance

Means Comparisons

Comparisons for all pairs using Tukey-Kramer HSD

Confidence Quantile

q*	Alpha
2.41260	0.05

HSD Threshold Matrix

Abs(Dif)-HSD

	low-performing	mid-performing	high-performing
low-performing	-0.17178	-0.10193	-0.02891
mid-performing	-0.10193	-0.12369	-0.05456
high-performing	-0.02891	-0.05456	-0.17178

Positive values show pairs of means that are significantly different.

Connecting Letters Report

Level		Mean
low-performing	A	0.63979942
mid-performing	A	0.59204953
high-performing	A	0.49692906

Levels not connected by same letter are significantly different.

Ordered Differences Report

Level	- Level	Difference	Std Err Dif	Lower CL	Upper CL	p-Value
low-performing	high-performing	0.1428704	0.0711999	-0.028906	0.3146471	0.1207
mid-performing	high-performing	0.0951205	0.0620403	-0.054558	0.2447989	0.2840
low-performing	mid-performing	0.0477499	0.0620403	-0.101929	0.1974284	0.7231

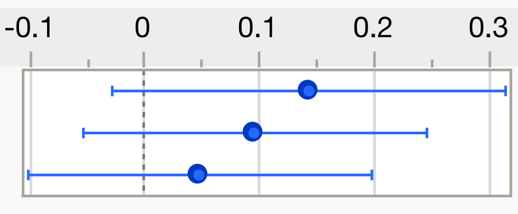

Excluded Rows 3

Oneway Analysis of Feeds purchase By Herd Indicator=CC-biogenic kgCO2eq

Oneway Anova

Summary of Fit

Rsquare	0.08169
Adj Rsquare	0.04637
Root Mean Square Error	0.01973
Mean of Response	0.013657
Observations (or Sum Wgts)	55

Analysis of Variance

Source	DF	Sum of Squares	Mean Square	F Ratio	Prob > F
Herd	2	0.00180073	0.000900	2.3129	0.1091
Error	52	0.02024280	0.000389		
C. Total	54	0.02204353			

Means for Oneway Anova

Level	Number	Mean	Std Error	Lower 95%	Upper 95%
high-performing	14	0.009631	0.00527	-0.001	0.02021
low-performing	14	0.023421	0.00527	0.0128	0.03400
mid-performing	27	0.010681	0.00380	0.0031	0.01830

Std Error uses a pooled estimate of error variance

Nonparametric Comparisons For All Pairs Using Steel-Dwass Method

q*	Alpha
2.34370	0.05

Level	- Level	Score Mean Difference	Std Err Dif	Z	p-Value	Hodges-Lehmann	Lower CL	Upper CL	Difference Plot
mid-performing	high-performing	1.89815	3.945227	0.481125	0.8802	0.001178	-0.006286	0.0074204	
low-performing	high-performing	1.35714	3.109126	0.436503	0.9003	0.001631	-0.006333	0.0176226	
mid-performing	low-performing	-1.68122	3.945227	-0.426139	0.9048	-0.000959	-0.013343	0.0061260	

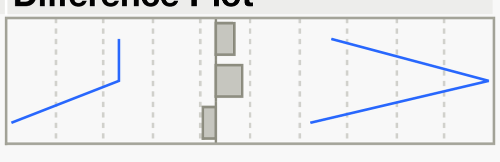

Excluded Rows 3

Oneway Analysis of Feeds purchase By Herd Indicator=CC-fossil kgCO2eq

Oneway Anova

Summary of Fit

Rsquare	0.029485
Adj Rsquare	-0.00784
Root Mean Square Error	0.124637
Mean of Response	0.27093
Observations (or Sum Wgts)	55

Analysis of Variance

Source	DF	Sum of Squares	Mean Square	F Ratio	Prob > F
Herd	2	0.02454097	0.012270	0.7899	0.4593
Error	52	0.80779212	0.015534		
C. Total	54	0.83233309			

Means for Oneway Anova

Level	Number	Mean	Std Error	Lower 95%	Upper 95%
high-performing	14	0.248270	0.03331	0.18143	0.31511
low-performing	14	0.252195	0.03331	0.18535	0.31904
mid-performing	27	0.292393	0.02399	0.24426	0.34053

Std Error uses a pooled estimate of error variance

Nonparametric Comparisons For All Pairs Using Steel-Dwass Method

q*	Alpha
2.34370	0.05

Level	- Level	Score Mean Difference	Std Err Dif	Z	p-Value	Hodges-Lehmann	Lower CL	Upper CL	Difference Plot
mid-performing	low-performing	3.95899	3.945227	1.00349	0.5746	0.028522	-0.057510	0.1268040	
mid-performing	high-performing	3.09127	3.945227	0.78355	0.7132	0.024405	-0.061365	0.1263781	
low-performing	high-performing	-0.07143	3.109126	-0.02297	0.9997	-0.001699	-0.120023	0.1082421	

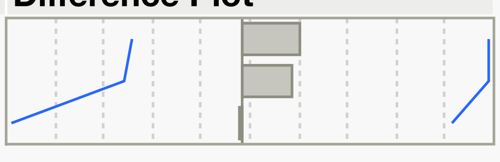

Excluded Rows 3

Oneway Analysis of Feeds purchase By Herd Indicator=CC-LTU kgCO2eq

Oneway Anova

Summary of Fit

Rsquare	0.153977
Adj Rsquare	0.121438
Root Mean Square Error	0.108707
Mean of Response	0.295405
Observations (or Sum Wgts)	55

Analysis of Variance

Source	DF	Sum of Squares	Mean Square	F Ratio	Prob > F
Herd	2	0.11183906	0.055920	4.7320	0.0129*
Error	52	0.61449695	0.011817		
C. Total	54	0.72633601			

Means for Oneway Anova

Level	Number	Mean	Std Error	Lower 95%	Upper 95%
high-performing	14	0.239028	0.02905	0.18073	0.29733
low-performing	14	0.364183	0.02905	0.30588	0.42248
mid-performing	27	0.288975	0.02092	0.24699	0.33096

Std Error uses a pooled estimate of error variance

Nonparametric Comparisons For All Pairs Using Steel-Dwass Method

q*	Alpha
2.34370	0.05

Level	- Level	Score Mean Difference	Std Err Dif	Z	p-Value	Hodges-Lehmann	Lower CL	Upper CL	Difference Plot
low-performing	high-performing	8.64286	3.109126	2.77983	0.0150*	0.106456	0.021020	0.2320285	
mid-performing	high-performing	8.08069	3.945227	2.04822	0.1009	0.055208	-0.016289	0.1179048	
mid-performing	low-performing	-7.10450	3.945227	-1.80078	0.1693	-0.060721	-0.154053	0.0238175	

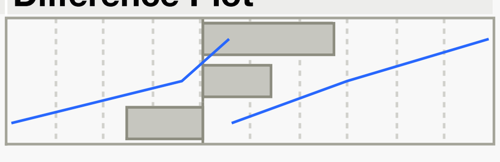

Excluded Rows 3

Oneway Analysis of Feeds purchase By Herd Indicator=POCP kgNMVOCeq

Oneway Anova

Summary of Fit

Rsquare	0.027704
Adj Rsquare	-0.00969
Root Mean Square Error	0.000367
Mean of Response	0.000803
Observations (or Sum Wgts)	55

Analysis of Variance

Source	DF	Sum of Squares	Mean Square	F Ratio	Prob > F
Herd	2	1.99143e-7	9.9572e-8	0.7408	0.4817
Error	52	6.98915e-6	1.3441e-7		
C. Total	54	7.1883e-6			

Means for Oneway Anova

Level	Number	Mean	Std Error	Lower 95%	Upper 95%
high-performing	14	0.000727	0.0001	0.00053	0.00092
low-performing	14	0.000763	0.0001	0.00057	0.00096
mid-performing	27	0.000863	0.00007	0.00072	0.00100

Std Error uses a pooled estimate of error variance

Nonparametric Comparisons For All Pairs Using Steel-Dwass Method

q*	Alpha
2.34370	0.05

Level	- Level	Score Mean Difference	Std Err Dif	Z	p-Value	Hodges-Lehmann	Lower CL	Upper CL	Difference Plot
mid-performing	high-performing	2.765873	3.945227	0.7010682	0.7628	0.0000644	-0.000180	0.0003397	
mid-performing	low-performing	1.681217	3.945227	0.4261395	0.9048	0.0000469	-0.000215	0.0003024	
low-performing	high-performing	1.071429	3.109126	0.3446076	0.9366	0.0000256	-0.000279	0.0003089	

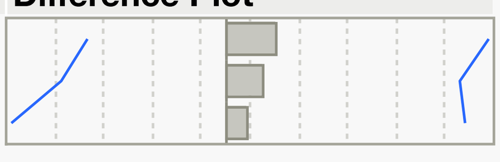

Excluded Rows 3
