# Supplementary material for: Climate Change and Photochemical Ozone Creation Potential Impact Indicators of Cow Milk: A Comparison of Different Scenarios for a Diet Assessment
Source: Animals (Basel). 2024 Jun 7;14(12):1725. doi: 10.3390/ani14121725 (PMC11201073; doi:10.3390/ani14121725)
Supplement: Supplementary file 1 [file Anova of In farm water use.pdf › animals-3004812-supplementary/Table 3/Anova of In farm water use.pdf]

Oneway Analysis of In farm water use By Herd Indicator=CC kgCO2eq

Oneway Anova

Summary of Fit

Rsquare	0.621141
Adj Rsquare	0.606569
Root Mean Square Error	0.000252
Mean of Response	0.001703
Observations (or Sum Wgts)	55

Analysis of Variance

Source	DF	Sum of Squares	Mean Square	F Ratio	Prob > F
Herd	2	5.39441e-6	2.6972e-6	42.6270	<.0001*
Error	52	3.29028e-6	6.3275e-8		
C. Total	54	8.68469e-6			

Means for Oneway Anova

Level	Number	Mean	Std Error	Lower 95%	Upper 95%
high-performing	14	0.001301	6.72e-5	0.00117	0.00144
low-performing	14	0.002174	6.72e-5	0.00204	0.00231
mid-performing	27	0.001667	4.84e-5	0.00157	0.00176

Std Error uses a pooled estimate of error variance

Nonparametric Comparisons For All Pairs Using Steel-Dwass Method

q*	Alpha
2.34370	0.05

Level	- Level	Score Mean Difference	Std Err Dif	Z	p-Value	Hodges-Lehmann	Lower CL	Upper CL	Difference Plot
mid-performing	high-performing	14.2632	3.945227	3.61531	0.0009*	0.000291	0.000120	0.000570	
low-performing	high-performing	13.6429	3.109126	4.38800	<.0001*	0.000816	0.000603	0.001202	
mid-performing	low-performing	-17.8426	3.945227	-4.52258	<.0001*	-0.000524	-0.000704	-0.000314	

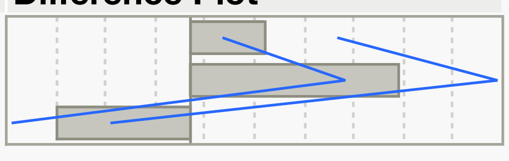

Excluded Rows 3

Oneway Analysis of In farm water use By Herd Indicator=CC-biogenic kgCO2eq

Oneway Anova

Summary of Fit

Rsquare	0.621141
Adj Rsquare	0.606569
Root Mean Square Error	8.505e-7
Mean of Response	5.758e-6
Observations (or Sum Wgts)	55

Analysis of Variance

Source	DF	Sum of Squares	Mean Square	F Ratio	Prob > F
Herd	2	6.1664e-11	3.083e-11	42.6270	<.0001*
Error	52	3.7611e-11	7.233e-13		
C. Total	54	9.9276e-11			

Means for Oneway Anova

Level	Number	Mean	Std Error	Lower 95%	Upper 95%
high-performing	14	0.0000044	2.273e-7	3.9432e-6	4.8554e-6
low-performing	14	7.3488e-6	2.273e-7	6.8927e-6	7.8049e-6
mid-performing	27	5.6375e-6	1.6367e-7	5.3091e-6	5.9659e-6

Std Error uses a pooled estimate of error variance

Nonparametric Comparisons For All Pairs Using Steel-Dwass Method

q*	Alpha
2.34370	0.05

Level	- Level	Score Mean Difference	Std Err Dif	Z	p-Value	Hodges-Lehmann	Lower CL	Upper CL	Difference Plot
mid-performing	high-performing	14.2632	3.945227	3.61531	0.0009*	9.8543e-7	4.0607e-7	1.9256e-6	
low-performing	high-performing	13.6429	3.109126	4.38800	<.0001*	2.7597e-6	2.0377e-6	4.0656e-6	
mid-performing	low-performing	-17.8426	3.945227	-4.52258	<.0001*	-1.772e-6	-2.379e-6	-1.062e-6	

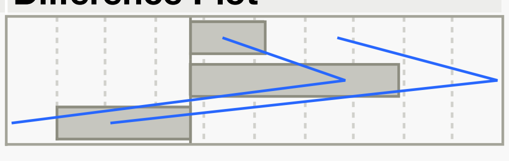

Excluded Rows 3

Oneway Analysis of In farm water use By Herd Indicator=CC-fossil kgCO2eq

Oneway Anova

Summary of Fit

Rsquare	0.621141
Adj Rsquare	0.606569
Root Mean Square Error	0.000251
Mean of Response	0.001696
Observations (or Sum Wgts)	55

Analysis of Variance

Source	DF	Sum of Squares	Mean Square	F Ratio	Prob > F
Herd	2	5.34972e-6	2.6749e-6	42.6270	<.0001*
Error	52	3.26301e-6	6.275e-8		
C. Total	54	8.61273e-6			

Means for Oneway Anova

Level	Number	Mean	Std Error	Lower 95%	Upper 95%
high-performing	14	0.001296	6.69e-5	0.00116	0.00143
low-performing	14	0.002165	6.69e-5	0.00203	0.00230
mid-performing	27	0.001660	4.82e-5	0.00156	0.00176

Std Error uses a pooled estimate of error variance

Nonparametric Comparisons For All Pairs Using Steel-Dwass Method

q*	Alpha
2.34370	0.05

Level	- Level	Score Mean Difference	Std Err Dif	Z	p-Value	Hodges-Lehmann	Lower CL	Upper CL	Difference Plot
mid-performing	high-performing	14.2632	3.945227	3.61531	0.0009*	0.000290	0.000120	0.000567	
low-performing	high-performing	13.6429	3.109126	4.38800	<.0001*	0.000813	0.000600	0.001197	
mid-performing	low-performing	-17.8426	3.945227	-4.52258	<.0001*	-0.000522	-0.000701	-0.000313	

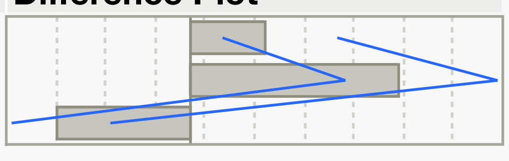

Excluded Rows 3

Oneway Analysis of In farm water use By Herd Indicator=CC-LTU kgCO2eq

Oneway Anova

Summary of Fit

Rsquare	0.621141
Adj Rsquare	0.606569
Root Mean Square Error	1.938e-7
Mean of Response	1.312e-6
Observations (or Sum Wgts)	55

Analysis of Variance

Source	DF	Sum of Squares	Mean Square	F Ratio	Prob > F
Herd	2	3.2036e-12	1.602e-12	42.6270	<.0001*
Error	52	1.954e-12	3.758e-14		
C. Total	54	5.1576e-12			

Means for Oneway Anova

Level	Number	Mean	Std Error	Lower 95%	Upper 95%
high-performing	14	1.0027e-6	5.1808e-8	8.9879e-7	1.1067e-6
low-performing	14	1.675e-6	5.1808e-8	1.5711e-6	1.779e-6
mid-performing	27	1.285e-6	3.7306e-8	1.2101e-6	1.3598e-6

Std Error uses a pooled estimate of error variance

Nonparametric Comparisons For All Pairs Using Steel-Dwass Method

q*	Alpha
2.34370	0.05

Level	- Level	Score Mean Difference	Std Err Dif	Z	p-Value	Hodges-Lehmann	Lower CL	Upper CL	Difference Plot
mid-performing	high-performing	14.2632	3.945227	3.61531	0.0009*	2.2461e-7	9.2557e-8	4.389e-7	
low-performing	high-performing	13.6429	3.109126	4.38800	<.0001*	6.2901e-7	4.6446e-7	9.2667e-7	
mid-performing	low-performing	-17.8426	3.945227	-4.52258	<.0001*	-4.04e-7	-5.423e-7	-2.421e-7	

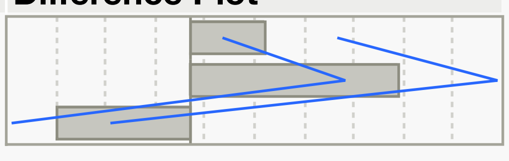

Excluded Rows 3

Oneway Analysis of In farm water use By Herd Indicator=POCP kgNMVOCeq

Oneway Anova

Summary of Fit

Rsquare	0.621141
Adj Rsquare	0.606569
Root Mean Square Error	6.876e-7
Mean of Response	4.655e-6
Observations (or Sum Wgts)	55

Analysis of Variance

Source	DF	Sum of Squares	Mean Square	F Ratio	Prob > F
Herd	2	4.0306e-11	2.015e-11	42.6270	<.0001*
Error	52	2.4584e-11	4.728e-13		
C. Total	54	6.489e-11			

Means for Oneway Anova

Level	Number	Mean	Std Error	Lower 95%	Upper 95%
high-performing	14	3.5568e-6	1.8377e-7	3.188e-6	3.9255e-6
low-performing	14	5.9414e-6	1.8377e-7	5.5726e-6	6.3101e-6
mid-performing	27	4.5578e-6	1.3233e-7	4.2923e-6	4.8233e-6

Std Error uses a pooled estimate of error variance

Nonparametric Comparisons For All Pairs Using Steel-Dwass Method

q*	Alpha
2.34370	0.05

Level	- Level	Score Mean Difference	Std Err Dif	Z	p-Value	Hodges-Lehmann	Lower CL	Upper CL	Difference Plot
mid-performing	high-performing	14.2632	3.945227	3.61531	0.0009*	7.967e-7	3.283e-7	1.5568e-6	
low-performing	high-performing	13.6429	3.109126	4.38800	<.0001*	2.2311e-6	1.6475e-6	3.2869e-6	
mid-performing	low-performing	-17.8426	3.945227	-4.52258	<.0001*	-1.433e-6	-1.924e-6	-8.588e-7	

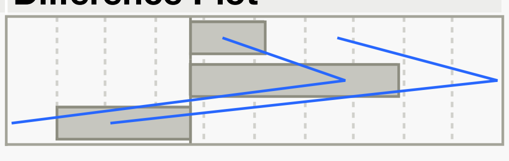

Excluded Rows 3
