# Supplementary material for: Climate Change and Photochemical Ozone Creation Potential Impact Indicators of Cow Milk: A Comparison of Different Scenarios for a Diet Assessment
Source: Animals (Basel). 2024 Jun 7;14(12):1725. doi: 10.3390/ani14121725 (PMC11201073; doi:10.3390/ani14121725)
Supplement: Supplementary file 1 [file Distribution of Barn management.pdf › animals-3004812-supplementary/Table 3/Distribution of Barn management.pdf]

Distributions Herd=high-performing, Indicator=CC kgCO2eq

Barn management

Summary Statistics

Mean	0
Std Dev	0
Std Err Mean	0
Upper 95% Mean	0
Lower 95% Mean	0
N	14
N Missing	0

Distributions Herd=high-performing, Indicator=CC-biogenic kgCO2eq

Barn management

Summary Statistics

Mean	0
Std Dev	0
Std Err Mean	0
Upper 95% Mean	0
Lower 95% Mean	0
N	14
N Missing	0

Distributions Herd=high-performing, Indicator=CC-fossil kgCO2eq

Barn management

Summary Statistics

Mean	0
Std Dev	0
Std Err Mean	0
Upper 95% Mean	0
Lower 95% Mean	0
N	14
N Missing	0

Distributions Herd=high-performing, Indicator=CC-LTU kgCO2eq

Barn management

Summary Statistics

Mean	0
Std Dev	0
Std Err Mean	0
Upper 95% Mean	0
Lower 95% Mean	0
N	14
N Missing	0

Distributions Herd=high-performing, Indicator=POCP kgNMVOCeq

Barn management

Compare Distributions

Show	Distribution		AICc ^	BIC	-2*LogLikelihood
☑	Normal		-144.6497	-144.4625	-149.7406

Summary Statistics

Mean	0.0051772
Std Dev	0.0011932
Std Err Mean	0.0003189
Upper 95% Mean	0.0058661
Lower 95% Mean	0.0044883
N	14
N Missing	0

Fitted Normal Distribution

Parameter		Estimate	Std Error	Lower 95%	Upper 95%
Location	μ	0.0051772	0.0003189	0.0044883	0.0058661
Dispersion	σ	0.0011932	0.0002386	0.000865	0.0019223

Measures	
-2*LogLikelihood	-149.7406
AICc	-144.6497
BIC	-144.4625

Goodness-of-Fit Test

	W	Prob<W
Shapiro-Wilk	0.9101979	0.1586

	A²	Simulated p-Value
Anderson-Darling	0.5447223	0.1372

Note: Ho = The data is from the Normal distribution. Small p-values reject Ho.

Distributions Herd=low-performing, Indicator=CC kgCO2eq

Barn management

Summary Statistics

Mean	0
Std Dev	0
Std Err Mean	0
Upper 95% Mean	0
Lower 95% Mean	0
N	14
N Missing	0

Distributions Herd=low-performing, Indicator=CC-biogenic kgCO2eq

Barn management

Summary Statistics

Mean	0
Std Dev	0
Std Err Mean	0
Upper 95% Mean	0
Lower 95% Mean	0
N	14
N Missing	0

Distributions Herd=low-performing, Indicator=CC-fossil kgCO2eq

Barn management

Summary Statistics

Mean	0
Std Dev	0
Std Err Mean	0
Upper 95% Mean	0
Lower 95% Mean	0
N	14
N Missing	0

Distributions Herd=low-performing, Indicator=CC-LTU kgCO2eq

Barn management

Summary Statistics

Mean	0
Std Dev	0
Std Err Mean	0
Upper 95% Mean	0
Lower 95% Mean	0
N	14
N Missing	0

Distributions Herd=low-performing, Indicator=POCP kgNMVOCeq

Barn management

Compare Distributions

Show	Distribution		AICc ^	BIC	-2*LogLikelihood
☑	Normal		-136.3353	-136.1481	-141.4262

Summary Statistics

Mean	0.008415
Std Dev	0.0016058
Std Err Mean	0.0004292
Upper 95% Mean	0.0093421
Lower 95% Mean	0.0074879
N	14
N Missing	0

Fitted Normal Distribution

Parameter		Estimate	Std Error	Lower 95%	Upper 95%
Location	μ	0.008415	0.0004292	0.0074879	0.0093421
Dispersion	σ	0.0016058	0.0003212	0.0011641	0.0025869

Measures	
-2*LogLikelihood	-141.4262
AICc	-136.3353
BIC	-136.1481

Goodness-of-Fit Test

	W	Prob<W
Shapiro-Wilk	0.9574909	0.6816

	A²	Simulated p-Value
Anderson-Darling	0.2245886	0.7848

Note: Ho = The data is from the Normal distribution. Small p-values reject Ho.

Distributions Herd=mid-performing, Indicator=CC kgCO2eq

Barn management

Summary Statistics

Mean	0
Std Dev	0
Std Err Mean	0
Upper 95% Mean	0
Lower 95% Mean	0
N	27
N Missing	0

Distributions Herd=mid-performing, Indicator=CC-biogenic kgCO2eq

Barn management

Summary Statistics

Mean	0
Std Dev	0
Std Err Mean	0
Upper 95% Mean	0
Lower 95% Mean	0
N	27
N Missing	0

Distributions Herd=mid-performing, Indicator=CC-fossil kgCO2eq

Barn management

Summary Statistics

Mean	0
Std Dev	0
Std Err Mean	0
Upper 95% Mean	0
Lower 95% Mean	0
N	27
N Missing	0

Distributions Herd=mid-performing, Indicator=CC-LTU kgCO2eq

Barn management

Summary Statistics

Mean	0
Std Dev	0
Std Err Mean	0
Upper 95% Mean	0
Lower 95% Mean	0
N	27
N Missing	0

Distributions Herd=mid-performing, Indicator=POCP kgNMVOCeq

Barn management

Compare Distributions

Show	Distribution		AICc ^	BIC	-2*LogLikelihood
☑	Normal		-323.5801	-321.4884	-328.0801

Summary Statistics

Mean	0.006434
Std Dev	0.0005665
Std Err Mean	0.000109
Upper 95% Mean	0.0066581
Lower 95% Mean	0.0062099
N	27
N Missing	0

Fitted Normal Distribution

Parameter		Estimate	Std Error	Lower 95%	Upper 95%
Location	μ	0.006434	0.000109	0.0062099	0.0066581
Dispersion	σ	0.0005665	7.933e-5	0.0004462	0.0007764

Measures	
-2*LogLikelihood	-328.0801
AICc	-323.5801
BIC	-321.4884

Goodness-of-Fit Test

	W	Prob<W
Shapiro-Wilk	0.9488537	0.2011

	A²	Simulated p-Value
Anderson-Darling	0.3437407	0.4644

Note: Ho = The data is from the Normal distribution. Small p-values reject Ho.
